# Physical Aging, Composition, and Carbon-Additive Identity as Separable Rheological Controls in Colloidal-Cellulose Nanofibril–Natural-Rubber-Latex Hydrogels

**DOI:** 10.3390/gels12100877

**Published:** 2026-09-28

**Authors:** Branko Alič, Urška Šebenik, Matjaž Krajnc

**Affiliations:** Faculty of Chemistry and Chemical Technology, University of Ljubljana, Večna pot 113, 1000 Ljubljana, Slovenia; branko.alic@fkkt.uni-lj.si (B.A.); urska.sebenik@fkkt.uni-lj.si (U.Š.)

**Keywords:** colloidal gel, physical aging, cellulose nanofibrils, natural-rubber latex, carbon additives, hydrogel rheology

## Abstract

Hydrogels prepared from TEMPO-oxidized cellulose nanofibrils (TCNFs), natural rubber latex (NRL), and carbon additives change their rheological properties during storage, so formulation comparisons made at uncontrolled aging states can be misleading. Here, TCNF–NRL hydrogels containing graphene conductive additive (GCA), graphene nanoplatelets (GNP), multi-walled carbon nanotubes (MWCNT), carboxylated multi-walled carbon nanotubes (MWCNT-COOH), or graphite were examined through four experimental sets that separate the effects of physical aging, total solids content, TCNF/carbon-additive ratio, carbon-additive loading, and additive identity. Oscillatory and steady-shear measurements were described with Cross and generalized Maxwell fits, and oscillatory-steady comparisons were quantified with a Cox–Merz deviation metric. Aging increased the storage modulus and the Cross low-shear viscosity by factors of 1.3 to 1.9 over about two weeks while the spectra remained elastic-dominated. At the late-storage comparison state, composition changed mainly the magnitude of the moduli and of the flow resistance: rankings by storage modulus and by low-shear viscosity agreed with Spearman rank coefficients of 0.89 to 1.00 across the four sets. At fixed base composition, the carbon additive changed the low-shear viscosity over a seven-fold range, from graphite and GCA at the low end to MWCNT at the high end, and the Cox–Merz deviation, expressed as the mean ratio of complex to steady-shear viscosity, ranged from 3.7 (GNP) to 7.3 (MWCNT). The late-storage states used for these comparisons are operational rather than equilibrated, and their ages differ between formulations, from 11 to 35 days, so the additive comparison was repeated at matched ages and the grouping was confirmed. No electrical or microstructural measurements were performed, so additive effects are reported as rheological observations, not as evidence of conductive-network formation.

## 1. Introduction

TEMPO-oxidized cellulose nanofibrils (TCNFs) provide a chemically defined, colloidally dispersed fibrillar scaffold for hydrogels. Fibril aspect ratio, surface carboxylate content, and aqueous dispersion state govern the formation of mechanically percolated networks [1,2,3]. Because these networks combine elastic storage, shear-thinning flow, and recovery after deformation, TCNF has become a central structural component in hydrogel design rather than a passive thickener [4,5,6]. In multicomponent systems, however, the rheological response is not determined by TCNF concentration alone. Latex particles, carbon additives, ionic environment, and preparation history all shift the modulus, the damping, and the flow resistance, so formulations must be compared at a controlled material state before any mechanistic interpretation is attempted.

Small-amplitude oscillatory shear and steady shear provide complementary, but not interchangeable, descriptions of structured soft materials. In the linear viscoelastic regime, the storage and loss moduli report the balance between elastic storage and viscous dissipation, while steady shear probes the network under continuous deformation [7,8,9]. Cox–Merz comparisons are useful as a diagnostic of deformation-mode sensitivity, but the rule cannot be assumed valid for hydrogels, colloidal gels, or filled polymer networks [10,11,12]. Rheology alone identifies elastic dominance, damping, shear thinning, and deformation-mode dependence. It does not establish microstructure or electrical transport pathways.

A further complication is physical aging. TCNF-rich and colloidal gel systems evolve over hours to days as local contacts reorganize and stress-bearing pathways consolidate [4,13,14,15]. If aging is not separated from composition, an apparent formulation effect may simply reflect a difference in structural maturity at the time of measurement. A defensible comparison among TCNF–NRL-carbon-additive formulations therefore requires two levels of control: time-resolved measurements on fixed formulations to quantify aging, and composition or additive comparisons performed at a defined late-storage state.

Natural rubber latex (NRL) is a practical aqueous matrix for this purpose because it supplies an elastic polymer phase while remaining compatible with dispersed fibrillar and particulate additives. Studies of nanocellulose-reinforced NRL composites show that cellulose fibrils strongly affect rheology, reinforcement, and network connectivity [16,17,18,19]. In TCNF–NRL hydrogels the fibrillar phase is a network-forming component that interacts with latex particles and carbon additives, not merely a viscosity modifier. The present work is a study of how formulation variables control the rheology of an aqueous TCNF–NRL–carbon system whose fibril, latex, and particulate components are all colloidally dispersed. It is not a carbon-filled elastomer study and not a conductive-hydrogel screening study. The word hydrogel is used here for a water-continuous, solid-like soft material, and colloidal gel where the colloidal nature of the network-forming units is the relevant aspect, as set out in Section 4.1. Additive identity is treated throughout as a formulation-level variable, that is the additive as supplied, and not as any single physicochemical parameter, because the five additives differ simultaneously in aspect ratio, particle size, specific surface area, surface chemistry, and morphology (Section 4.1).

Carbon additives introduce an additional design axis through differences in aspect ratio, surface chemistry, aggregate morphology, and dispersion stability. Carbon nanotubes, graphene-derived additives, and graphite alter low-shear viscosity, modulus, damping, and stress transfer in polymer and latex composites, but such rheological effects do not prove electrical percolation [20,21,22]. Aqueous dispersion studies further show that surfactant chemistry, co-coagulation sequence, and interfacial stabilization influence both mechanical and transport behavior [23,24,25,26,27]. In the absence of electrical measurements, carbon additives are treated here as rheological modifiers of a TCNF–NRL hydrogel network.

Previous work on conductive hydrogels, nanocellulose gels, and carbon-filled rubber composites establishes the relevance of mechanics, filler dispersion, and additive morphology, yet many studies emphasize final material performance without separating aging, base formulation, and additive identity experimentally [28,29,30,31,32]. For TCNF-structured latex hydrogels it therefore remains unclear whether a measured change in rheology arises from time-dependent network consolidation, from quantitative scaling with solids content and TCNF/carbon-additive ratio, or from the identity of the carbon additive.

The gum-based nanocomposite hydrogels with which materials of this kind are most often compared are covalently crosslinked networks. Gum acacia or gum ghatti is grafted with poly(acrylic acid) or a related monomer, and graphene oxide, oxidized nanotubes or ferrite particles are dispersed in the network that polymerization forms [33,34]. In those systems the filler modifies a chemically bonded network whose connectivity is fixed at synthesis, and its effect is read from rheology together with diffraction, electron microscopy, infrared spectroscopy, swelling, and thermal analysis. The TCNF–NRL system studied here has no polymerization step and no covalent crosslinks. Its connectivity arises from physical association between pre-formed fibrils, latex particles, and carbon particulates, which is why it keeps evolving for weeks after preparation and why aging has to be treated as a variable of the design rather than as a detail of the preparation. The present work also stops at rheology, so the structural evidence that accompanies the grafted-gum studies has no counterpart here, and Section 2.4 discusses the physical association among the components on the basis of the literature and the present data, while Section 2.5 labels these mechanistic readings as hypotheses for that reason.

The present study addresses this gap. We hypothesized that the macroscopic rheological response of TCNF–NRL–carbon hydrogels is governed by three separable variables: physical aging, TCNF/carbon-additive composition, and carbon-additive identity. Aging was expected to strengthen an already elastic-dominated network, composition to tune modulus, and flow resistance mainly through quantitative scaling, and additive identity to produce distinct responses under otherwise fixed composition. Four experimental sets were designed to test this hypothesis (Table 1). Oscillatory frequency sweeps, damping analysis, steady-shear flow curves, Cross fits, generalized Maxwell fits, Cole–Cole plots, and quantified Cox–Merz comparisons were used within the experimentally accessible windows. The novelty of the study is not the use of carbon additives in a latex hydrogel, which is established, but the experimental separation of physical aging, formulation composition, and additive identity in one system, so that each can be attributed a magnitude. The objective is a rheological design map for TCNF–NRL hydrogels with carbon additives: which formulation variables control stiffness, damping, and flow resistance, and by how much. The intended use of such a map is the formulation of printable and extrudable precursors, where the design constraints are the yield-like response that lets a deposited bead hold its shape and the stability of that response during storage before use. Whether such a precursor is also electrically useful is a separate question that these measurements do not address. Electrical functionality, microscopic organization, and molecular relaxation mechanisms are outside the scope of the data and are not claimed.

## 2. Results and Discussion

The results are organized around the three variables of the design: physical aging (Set 1, Section 2.1), formulation composition (Sets 1-3, Section 2.2), and carbon-additive identity (Set 4, Section 2.3). This order matters because the hydrogels evolve during storage. Composition and additive comparisons are made at the late-storage states defined in Table 1 and are interpreted only after the aging behavior is established. Section 2.4 discusses the physical association among the components, and Section 2.5 states the limitations of the dataset and separates demonstrated results from hypotheses. Every curve shown in the figures is a single measurement on one loading of one preparation, not an average over replicates, for the reasons set out in Section 4.3.

Aging times of the late-storage states differ among formulations (11 to 35 days, Table 1) because storage measurements were scheduled per batch. The consequences of this inequality for the composition and additive comparisons are quantified in Section 2.2, Section 2.3 and Section 2.5.

### 2.1. Physical Aging

Figure 1 shows *G′* and *G″* at 1 Hz as functions of aging time for the five Set 1 formulations. *G′* exceeds *G″* at every measured age, including the earliest (1 h), and both moduli increase during storage at every solids content. The materials are therefore elastic-dominated gels from the first measurement onward, and aging strengthens the existing gel rather than creating one. No *G′*/*G″* crossover occurs at any age or frequency. Within the measured window this excludes an observable fluid-to-gel transition, although it does not date the gelation itself, which occurred before the first measurement and would require a time-resolved protocol immediately after preparation to observe.

The damping factor at 1 Hz (Figure 2) stays well below unity for all formulations and is lower at the final measured age than at 1 h. The viscous contribution therefore declines relative to elastic storage during aging, gradually and without abrupt transitions at the sampled ages.

Frequency sweeps at selected ages (Figure 3) show the same picture across the full analysis window: the spectra shift upward with age while their shape and the *G′* > *G″* ordering are retained [7,35]. In panel d (35 wt%) the 7-day and 11-day spectra nearly coincide, which reflects the smaller residual aging drift at high solids (below). The generalized Maxwell model (GMM) curves describe the aged spectra well but deviate from the 1 h spectra at low frequency. The likely reason is that the material still evolves during the 1 h sweep itself, whereas the model assumes a time-invariant spectrum. Later aged states are therefore the appropriate basis for composition comparisons. Cole–Cole representations of the same data (Figure 4) translate toward higher moduli with age while retaining similar curvature, consistent with a broad relaxation distribution whose magnitude, more than its shape, changes with age [36].

The apparent sensitivity of the modulus to solids content also changes with age: at 1 h the modulus–solids relationship is steeper than at the late-storage states. Solids content and aging are therefore coupled design variables, and formulation comparisons made before a defined late-storage state can overstate composition effects. This is an empirical observation, not evidence of a specific percolation or load-transfer mechanism.

Steady-shear flow curves (Figure 5) show pronounced shear thinning at all ages and solids contents, with the viscosity level rising during storage. The Cross fits (lines in Figure 5) capture the measured curves with *R*^2^ > 0.993 and provide *η*_0_ as a comparison quantity. Between 1 h and the final measured age, *η*_0_ increases by factors of 1.86, 1.84, 1.76, 1.34, and 1.38 for 20, 25, 30, 35, and 40 wt% solids, respectively (Supplementary Table S1). The relative aging effect is largest at low solids. The *η*_0_-versus-time trend is close to linear for four of the five formulations, with a coefficient of determination between 0.888 and 0.943 against 0.728 to 0.975 for a power-law description of the same data, so no universal aging exponent is assigned (Supplementary Table S10). Neither description is used outside the measured window. As a stabilization measure, the relative change of *G′* (1 Hz) per day over the final measured interval is 0.4 to 5.5% per day (Supplementary Table S4). The materials are therefore not fully equilibrated at the last measured age. “Late-storage state” in this work is an operational comparison point, not an equilibrium.

The corresponding shear-stress curves (Figure 6) contain weak non-monotonic features at intermediate shear rates that move to higher stress with age. Because the flow protocol imposes a monotonically increasing shear rate, a non-monotonic *σ*($\dot{\gamma }$) record means that the stress fell while the imposed rate continued to rise. A flow curve with a locally negative slope is mechanically unstable, which is characteristic of a material yielding heterogeneously rather than of a smooth constitutive flow curve. Wall slip is partly suppressed by the cross-hatched plates but not excluded, and transient structural breakdown is the most plausible reading of the data. These features are referred to as yield-like throughout, and not as a yield stress, because no stress ramp, creep, step-stress, hysteresis, or recovery experiment was performed. The stress level at which they occur is 10 to 100 Pa depending on formulation and age. Local velocimetry or rheo-optical measurements would be required to identify the mechanism definitively [11,12,37].

Mechanistically, the aging trends are consistent with the following hypothesis. TCNF fibrils form a percolated scaffold whose junctions consolidate during quiescent storage. Latex particles and carbon particles are trapped in and attached to this scaffold, and slow rearrangement of fibril–fibril and fibril–particle contacts transfers an increasing share of stress to the elastic network, raising *G′* and *η*_0_ while leaving the shape of the spectrum unchanged. The alternative explanations, irreversible aggregation, water redistribution, or slow latex film formation, cannot be excluded by rheology alone. Recovery and rejuvenation experiments would discriminate between reversible physical aging and irreversible structural change and were not part of this study (Section 2.4 and Section 2.5).

### 2.2. Composition Effects

Figure 7 compares the frequency dependence of *G′* and *G″* at the late-storage states for the three-composition series: total solids (Set 1), TCNF/GCA ratio at fixed solids (Set 2), and GCA loading at fixed total solids and fixed NRL/TCNF mass ratio (Set 3). In all three series *G′* exceeds *G″* at every frequency with no crossover, and the spectra remain approximately parallel on logarithmic axes.

The three series differ in the size of the effect. Increasing total solids from 20 to 40 wt% raises *G′* (1 Hz) by an order of magnitude. Shifting the TCNF/GCA ratio from 0.03/0.12 to 0.15/0.00 at constant total solids raises *G′* (1 Hz) by a factor of about 18 (103 to 1880 Pa), which identifies TCNF, not GCA, as the component that dominates the rheological response: replacing GCA mass by TCNF mass strengthens the network monotonically. That the fibrils dominate the response is a rheological observation. That they do so by forming the load-bearing network is an interpretation, which no structural measurement in this work tests. Changing the GCA loading from 0 to 0.25 at fixed total solids and fixed NRL/TCNF mass ratio (Set 3) produces only small shifts of *G′* that partly overlap between neighboring loadings. This series is not at constant NRL and TCNF concentration. Because the total solids are held at 25 wt%, adding GCA dilutes the other two components, and across the series the NRL concentration falls from 23.4 to 18.9 wt% and the TCNF concentration from 1.64 to 1.33 wt%, both by 19%. The observed decline of *G′* is of the size that this dilution alone would produce: scaling the matched-age moduli of Set 2 against TCNF concentration gives a power law with exponent 1.79 and *R*^2^ of 0.998, and applying that exponent to the Set 3 concentrations reproduces the measured moduli to within 6% on average and 19% at worst, with no positive term for GCA. The design therefore cannot isolate a contribution of GCA from the accompanying dilution. The effect of GCA loading on the modulus is close to the resolution of the comparison, particularly since the late-storage ages of the Set 3 formulations differ (14 to 35 days, Table 1) and the residual drift of about 0.4 to 1.1% per day over a several-day age difference is comparable to the differences between adjacent loadings.

The damping factor (Figure 8) remains far below unity for all compositions. Higher solids or TCNF content lowers tan *δ* moderately, indicating a further shift toward elastic storage. The frequency position of the shallow tan *δ* minimum changes little across the formulations, so composition modifies mainly the strength of the network rather than the balance of the resolved relaxation processes. Cole–Cole trajectories (Figure 9) shift toward higher moduli with composition while keeping their compressed, non-semicircular curvature, which indicates a broad distribution of relaxation behavior rather than a single dominant mode [36]. Neither representation reveals a new spectral feature in any composition series. The dominant compositional effect is a change of scale, not of spectral shape, within the analysis window of 0.01 to 6.31 Hz. Statements about relaxation behavior outside this window are not supported by the data.

Steady-shear flow curves (Figure 10) show strong shear thinning for all compositions. At high shear rates the curves of the different compositions converge, so composition effects are strongest under weak deformation, where the intact network dominates the flow resistance. Four readings of that convergence are possible, and the present data do not separate them: progressive alignment of the structure, its genuine breakdown, wall slip that grows with rate, and fracture or expulsion of the sample at the plate edge. Two of them can be bounded. The torque at the highest rates is three orders of magnitude above the resolution of the instrument, so instrumental limitation is not among the candidates, and the normal force stayed within ±1 N with no expulsion observed except in one aged sample (Section 2.3), which bounds but does not exclude edge fracture. Distinguishing alignment from breakdown, and either from residual slip, requires structural or flow-field measurements [3,4]. The Cross *η*_0_ spans about 9 × 10^3^ to 2 × 10^5^ Pa·s across Sets 1–2 and correlates with the oscillatory ranking: rankings of the formulations by *G′* (1 Hz) and by *η*_0_ agree perfectly in Sets 1 and 2 (Spearman ρ = 1.00, exact two-sided permutation *p* = 0.017 and 0.003) and to ρ = 0.89 in Set 3 (*p* = 0.033), where the *η*_0_ values of all six loadings lie between 49,000 and 68,000 Pa·s, a total spread comparable to the width of the individual 95% confidence intervals (Supplementary Table S1). The quantitative conclusion for Set 3 is therefore negative: raising the GCA loading to 0.25 at fixed total solids does not change the low-shear viscosity resolvably. Because that series dilutes NRL and TCNF as GCA is added (Section 2.2), the result bounds the combined change and is not evidence that GCA itself is rheologically inert. Composition acts on the flow resistance chiefly through total solids and the TCNF share.

The Cox–Merz comparison (Figure 11) shows that |η*(*ω*)| exceeds *η*($\dot{\gamma }$) at matched arguments for every formulation. The deviation metric (Section 4.4) makes the trend quantitative: the mean ratio |η*|/*η* grows monotonically with solids content from 2.3 (20 wt%) to 7.4 (40 wt%) in Set 1 and with the TCNF share from 3.0 to 7.2 in Set 2, while it is nearly constant, 3.9 to 5.0, across the GCA loadings of Set 3 (Supplementary Table S3). Across all four sets the deviation tracks the strength of the network rather than the composition variable that produced it: over the 19 distinct preparations the mean ratio correlates with the low-shear parameter at a Spearman coefficient of 0.74 and with *G′* (1 Hz) at 0.77 (Supplementary Figure S6). Two alternative readings can be set aside. The accessible window is not the cause, because the overlapping range is the same for every formulation, 2.80 decades with 14 measured steady-shear and 15 measured oscillatory points (Supplementary Table S8). Nor is the trend a threshold effect of the yield-like features, since the ratio rises continuously over a threefold range rather than stepping once those features appear. The hydrogels therefore cannot be treated as simple viscoelastic liquids. Stronger networks differ more strongly between oscillatory and steady deformation, which is the expected behavior of structured materials whose network is progressively broken down under continuous shear but remains intact under small-amplitude oscillation [10,12]. The microscopic origin of the deviation is not identified by these measurements.

A plausible colloidal picture for the composition series, stated as hypothesis, is that total solids and TCNF content control the density of the fibrillar scaffold and its fibril–fibril junction number, while GCA at fixed TCNF occupies the network without adding load-bearing connectivity, consistent with the absence of a resolvable effect on *η*_0_ and with the weak effect on *G′*, although the Set 3 series cannot separate either from the dilution of NRL and TCNF that accompany added GCA (Section 2.2). Testing this picture would require microscopy or scattering of the network structure, which was not performed (see also Section 2.4).

### 2.3. Carbon-Additive Identity Effects

Set 4 compares five carbon additives at fixed base composition (NRL:TCNF:CA = 1.0:0.07:0.15, 25 wt% solids). The late-storage ages of the five formulations differ (13 to 35 days, Table 1). The GCA sample was measured to 35 days, the others to 13 to 15 days. Because the residual aging drift at these ages is of the order of 0.2 to 3% per day for four of the five formulations (11% per day for MWCNT, which was still aging measurably at 15 days), moderate age differences shift the moduli by far less than the differences between additives discussed below, with one exception noted explicitly.

Figure 12 shows *G′* and *G″* at the late-storage state. All five systems are elastic dominated with no crossover. The additive changes the modulus level substantially: *G′* (1 Hz) is 2510 Pa for MWCNT, 1490 Pa for GNP and MWCNT-COOH, 552 Pa for GCA, and 446 Pa for graphite. The ordering is preserved across the frequency window. The damping factor at 1 Hz (Figure 13) spans 0.075 (GNP) to 0.101 (MWCNT and MWCNT-COOH): the nanotube systems combine the highest moduli with the highest relative dissipation. Cole–Cole trajectories (Figure 14) are separated in position and curvature, so the additive changes not only the modulus scale but also the balance between elastic and viscous response.

Steady-shear data (Figure 15) show the same grouping under flow. With increasing shear rate the curves converge, so the additive-dependent differences are strongest at low shear and diminish under stronger deformation, consistent with progressive breakdown or alignment of the stress-bearing structures, which the present data do not identify directly. The Cross *η*_0_ spans a seven-fold range, from 47,000 Pa·s (graphite) and 58,000 Pa·s (GCA) to 162,000 (MWCNT-COOH), 170,000 (GNP), and 338,000 Pa·s (MWCNT). The 95% confidence intervals of *η*_0_ are ±18 to ±38% (Supplementary Table S1), well below the spread between the low and high groups. Releasing the *n* ≤ 1 bound of the Cross fits lowers *η*_0_ by a median of 7% and narrows this range to 5.8-fold, leaving the grouping unchanged (Supplementary Table S6). Rankings by *G′* (1 Hz) and by *η*_0_ agree to ρ = 0.97, with an exact two-sided permutation p of 0.033 (Supplementary Table S9). With five formulations the smallest attainable *p* is 0.017, so this is a descriptive statement about two measured rankings and not evidence of a mechanism. The only rank instability concerns the closely spaced pair GCA–graphite, whose order also depends on the comparison age (the GCA value refers to 35 days, whereas at 6 days its *η*_0_ of 44,000 Pa·s lay below the 14-day graphite value). Because the late-storage measurements of the five additives fall at different storage times, from 13 to 35 d (Table 1), the comparison was repeated at ages common to all five. Interpolating each additive to 6 d and to 13 d, both inside the measured window of every formulation, the separation between the high-response and low-response groups is 2.8-fold and 3.0-fold in *G′* (1 Hz) and 3.4-fold and 3.3-fold in *η*_0_, and the ordering is unchanged in four of five positions. Interpolating on a linear rather than a logarithmic scale changes no interpolated value by more than 4.6% and leaves the ordering identical. The trajectories and the two matched ages are shown in Supplementary Figure S3 and the interpolated values with their bracketing measurements in Supplementary Table S13. Robust statements are therefore about the groups: nanotube and nanoplatelet additives produce a several-fold stronger network response than GCA and graphite, while the order within the low group is not resolved.

The Cox–Merz deviation depends on the additive as well (Figure 16, Supplementary Table S3): the mean |η*|/*η* ratio is 7.3 for MWCNT, 5.1 for MWCNT-COOH, 4.2 for graphite, 4.0 for GCA, and 3.7 for GNP. The strongest network (MWCNT) is also the most sensitive to the deformation mode. Deformation-mode sensitivity is thus an additive-dependent design property alongside stiffness and flow resistance.

Which physical differences between the additives cause this grouping cannot be decided from rheology alone, because the five additives differ simultaneously in aspect ratio, particle size, specific surface area, surface chemistry, and dispersion behavior (Section 4.1). The observed grouping is consistent with the hypothesis that high-aspect-ratio nanotubes and large-area nanoplatelets couple to the TCNF–NRL network as bridging or entangled elements, raising modulus and low-shear viscosity, while compact GCA particles and graphite flakes act mainly as dispersed filler. The apparent effect of carboxylation (MWCNT-COOH versus MWCNT) depends on the comparison age. At the late-storage states reported above, which fall at different storage times for the two systems, the *η*_0_ of MWCNT-COOH is about half that of MWCNT. At matched storage times the two are much closer, with *η*_0_ ratios of 0.98 at 6 days and 1.60 at 13 days, so the contrast reflects a difference in how fast the two systems stiffen rather than a difference in the aged state. These data therefore do not show that carboxylation lowers the modulus or the low-shear viscosity. These interpretations remain hypotheses [20,21,22,30] and are discussed further in Section 2.4. Literature on carbon-filled latex and rubber systems shows that dispersion protocol, matrix polarity, and interfacial chemistry strongly influence filler-network formation [24,26,31], which supports treating the present ranking as specific to the preparation protocol of Section 4.2.

### 2.4. Physical Association Among the Components

The preparation uses a prevulcanized latex, whose crosslinks lie inside the rubber particles and were formed before mixing, and it includes no deliberate reaction between components and no added crosslinker (Section 4.1 and Section 4.2). The gel response is therefore interpreted in terms of physical association among the dispersed components. This section considers fibril–fibril, fibril–latex, and carbon–fibril or carbon–latex association in the light of related literature and of the constraints set by the present data. Carbon–carbon contacts, which could form a filler network of their own [21], and latex–latex contacts (Section 2.1) are not excluded. The mechanisms are transferred from related materials and remain hypotheses for the present system (Section 2.5).

Fibril–fibril association. The fibrils carry 1.4 mmol g^−1^ of carboxylate groups (Section 4.1), and dispersions of TEMPO-oxidized fibrils are stabilized by electrostatic repulsion between the ionized groups [1,2]. In related systems of such fibrils with graphene oxide, calcium addition markedly increased elasticity, which was interpreted as ionic bridging of the carboxylate groups [5,38]. No multivalent salt was added here. The nominal sodium counterion content is about 12 to 61 mmol per kg of aqueous phase, with 3 mmol kg^−1^ of sodium azide in addition (Section 4.1). If these counterions were fully dissociated and no other electrolyte were present, the Debye screening length would be about 1.7 to 3.2 nm. The estimate is illustrative only, because counterion association, the unanalyzed latex serum and the unmeasured pH shift it in either direction. Aggregation of such fibrils on salt addition is driven mainly by specific association of counterions with the carboxylate groups rather than by screening [39]. With sodium as the only declared counterion, entanglement and other short-range contacts between fibrils are more plausible network junctions than ionic bridges, although the data do not distinguish them. The data do show a strong dependence on fibril content: *G′* (1 Hz) in Set 2 scales with TCNF concentration with an exponent of 1.79, and the same scaling reproduces Set 3 within 6% on average without an explicit GCA term (Section 2.2). Dispersions of TEMPO-oxidized fibrils alone keep stiffening for weeks after preparation [40], so consolidation of fibril contacts is one plausible contributor to the aging in Section 2.1.

Fibril–latex association. A mixed protein–phospholipid surface layer has been reported for freshly tapped natural rubber latex particles [41], and latex concentrates owe their colloidal stability to the negative charge of the particle surface [42]. The surface of the prevulcanized latex used here was not characterized. If both components remain negatively charged, their double-layer interaction is repulsive on average, and association is more plausibly geometric. The fibrils, about 50 nm wide and 0.5 to 80 µm long according to the supplier (Section 4.1), are long compared with latex particles, typically 0.3 to 2 µm in diameter [42], so longer fibrils can constrain the particles within a fibrillar network. Three observations are consistent with this reading without proving it. In Set 2, at constant NRL concentration and total solids, replacing GCA by TCNF raises *G′* (1 Hz) 18-fold, from 103 to 1880 Pa (Section 2.2). The formulation without TCNF was unstable during preparation and the lowest-TCNF formulation was close to the feasibility boundary (Section 4.2), so TCNF was needed for a stable preparation. The GNP sample coagulated and was expelled at the highest shear rates (Figure 15), so this stability is limited under strong shear, although the component responsible was not identified.

Carbon-fibril and carbon-latex association. Nanocellulose disperses carbon nanotubes in water without chemical functionalization of the tubes or added surfactant [43,44]. Adhesion measurements on model graphene and nanocellulose surfaces show an association, interpreted as counterion fluctuations polarizing the carbon surface, and the nanocellulose charge was proposed to stabilize the resulting complexes [44]. Here the carbon additive was sonicated with the TCNF dispersion before the latex was added (Section 4.2), so the carbon surfaces met the fibrils first. In the formulation without TCNF the carbon lacked this dispersing partner. In natural rubber latex, the dispersion treatment of nanotubes changes their interaction with the polymer and their rheological effect [22], and nanotube networks in polymers depend on tube geometry and dispersion [20,21]. Because the five additives differ in several properties at once (Section 2.3), the data cannot decide which property controls their coupling to the network. At matched ages of 6 and 13 days, the nanotube and nanoplatelet group exceeds GCA and graphite by factors of 2.8 and 3.0 in *G′* (1 Hz) and 3.4 and 3.3 in *η*_0_ (Section 2.3). The two nanotube grades, MWCNT-COOH and MWCNT, give *η*_0_ ratios of 0.98 and 1.60 at the same ages, so the carboxylated grade shows no consistent reduction. The grouping is therefore consistent with a contribution of additive geometry, for example bridging by nanotubes or nanoplatelets within the fibril network, but does not separate geometry from surface area, surface chemistry, or dispersion state.

Consequences for the rheology. Networks of physical contacts are expected to respond differently to continuous shear and to small-amplitude oscillation [10,12], which is consistent with the Cox–Merz deviation growing with network strength (Section 2.2) and with MWCNT, the additive with the highest modulus, also giving the largest deviation (Section 2.3). Aging that raises the moduli at an approximately constant spectral shape (Section 2.1) is likewise compatible with progressive reorganization of physical contacts, as in other colloidal gels [13,14,15]. Measurements of pH and electrokinetic properties, variation of electrolyte concentration and valence, rejuvenation and recovery tests, and structural or interfacial methods would test these readings. None were part of this study (Section 2.5).

### 2.5. Scope and Limitations

The following limitations bound the conclusions.

Measurement scope. No electrical, microscopic, scattering, or spectroscopic measurements were performed. All statements about network structure, filler connectivity, particle dispersion, and conductive pathways are hypotheses, and the additive named GCA is not claimed to confer electrical conductivity. The words reinforcement and strength are likewise not used for the present results. What is measured is an increase in rheological moduli and in flow resistance, which is not a measurement of strength, toughness or load-bearing capacity, and no such property is claimed.

Aging state. The late-storage comparison states are operational, not equilibrated. Residual drift of *G′* (1 Hz) is 0.2 to 5% per day at the final measured ages (11% per day for MWCNT), and comparison ages differ among formulations (Table 1). The material also ages during the measurement itself, but not enough to matter: a frequency sweep takes about 20 min (Section 4.3), which at the largest observed drift rate corresponds to a change of about 0.15% in *G′* (1 Hz) between the first and the last point of a sweep, well below the differences the sweeps are used to resolve. Grouped conclusions are robust to the inequality of comparison ages. Orderings within closely spaced pairs (GCA versus graphite, neighboring GCA loadings in Set 3) are not.

Aging mechanism. The data do not distinguish reversible physical aging from irreversible structural evolution, water redistribution, or slow latex-particle processes. Recovery and rejuvenation protocols would be required.

Flow response. Thixotropy and structural recovery after shear were not measured. The non-monotonic stress features indicate heterogeneous yielding, but the mechanism (structural breakdown, shear localization, residual wall slip) is not identified. Gap and plate-surface variation were not performed, so wall slip is suppressed but not excluded. The conclusions this affects are those that depend on the shape of the flow curve at high rates, that is the convergence of the curves and the non-monotonic stress features. The conclusions it does not affect are the comparisons at low shear and in oscillation, where the accumulated strain is small and the ranking is reproduced by two independent observables, the storage modulus and the low-shear parameter, measured under different deformation modes.

Dispersion history. All additives received the same sonication energy. Equal energy does not guarantee equal dispersion quality across additives of different morphology, so part of the additive ranking may reflect protocol-specific dispersion states rather than intrinsic additive properties.

Replication and uncertainty. The reported curves are single measurements, one batch per formulation and one loading per formulation and storage time, with reproducibility checked only at the start of the campaign and only on one to two samples (Section 4.3). Reported uncertainties are the 95% confidence intervals of the model fits, which capture measurement scatter within a curve but neither loading-to-loading nor batch-to-batch variation.

## 3. Conclusions

This study provides a rheological design map for TCNF-structured natural rubber latex hydrogels containing carbon additives, based on four experimental sets that separate physical aging, composition, and additive identity.

Physical aging strengthens an already formed elastic-dominated network: *G′* exceeds *G″* from 1 h onward, no crossover appears, and the Cross low-shear viscosity rises by factors of 1.3 to 1.9 over roughly two weeks, approximately linearly in time. The materials are not fully equilibrated at the last measured age, so all formulation comparisons refer to defined late-storage states.

Composition tunes the magnitude of the response without changing its character within the measured window. Total solids and the TCNF share are the effective levers: both raise modulus and low-shear viscosity monotonically, with rank correlations of 1.00 between oscillatory and steady-shear orderings. Raising the GCA loading at fixed total solids leaves the response almost unchanged, and its effect on the low-shear viscosity is not resolvable outside the fit uncertainty. That series also dilutes NRL and TCNF, so the result bounds the combined change rather than showing that GCA itself is inert.

Additive identity is the strongest differentiator at fixed composition. Nanotube and nanoplatelet additives (MWCNT, MWCNT-COOH, GNP) produce a several-fold higher modulus and low-shear viscosity than GCA and graphite, with a seven-fold total *η*_0_ span, and the Cox–Merz deviation ratio, 3.7 to 7.3, identifies deformation-mode sensitivity as an additional additive-dependent design property. Within the low-response group the ordering is not resolved.

Persistent, quantified Cox–Merz deviations show that oscillatory and steady-shear measurements are not interchangeable for these hydrogels. Cross and GMM fits serve as comparison tools. The GMM parameters are explicitly not identifiable individually, and no conclusion rests on them. The map is intended for the formulation of printable and extrudable precursors, where the operative constraints are the yield-like response that lets a deposited bead hold its shape and the stability of that response during storage before use. Electrical conductivity, microstructure, thixotropic recovery, and yield criteria require dedicated measurements before any application claim can be made.

The conclusions are not equally secure, and it is worth saying which are which. The most robust rest on many curves and on large effects: elastic dominance at every age and composition with no crossover in the measured window, monotonic stiffening during storage, total solids content and the TCNF share as the effective composition levers, the separation of the five additives into a high-response and a low-response group, which survives matching the comparison ages, and the persistence of a Cox–Merz deviation in every formulation. Four conclusions need more caution. The ordering within the low-response group is not resolved. The effect of GCA loading cannot be separated by the design of Set 3 from the dilution of NRL and TCNF that accompanies it. The comparison between MWCNT and MWCNT-COOH depends on the age at which it is made. Every mechanistic reading of the network remains unsupported by any structural measurement. Section 2.4 relates these readings to the physical interactions known for fibrils, latex particles and carbon surfaces and to the constraints set by the present data, but does not turn them into findings.

Each of those cautions names a measurement that would settle it. Cryogenic electron microscopy or small-angle scattering would test whether the fibrils form the load-bearing scaffold the rheology suggests. Local velocimetry or rheo-optical observation would identify the mechanism behind the yield-like features and separate wall slip from bulk yielding. A rejuvenation and recovery protocol would distinguish reversible physical aging from irreversible structural change. Electrical measurement would settle whether the rheological grouping of the additives has any counterpart in conductivity, which is the question the additive names invite and which this work does not answer. Preparing independent batches of a subset of the formulations would supply the batch-level uncertainty that the present dataset cannot provide.

## 4. Materials and Methods

### 4.1. Materials

Prevulcanized natural rubber latex (NRL, 61 wt% aqueous dispersion; Gumiform) was used as the polymer matrix. TEMPO-oxidized cellulose nanofibrils (TCNFs) were supplied by Cellulose Lab Inc. (Fredericton, NB, Canada) as a TEMPO-modified, freeze-dried sodium carboxylate cellulose nanofibril powder and were re-dispersed in deionized water to prepare the 6 wt% stock dispersion used in this study. The supplier describes the material as a web-like cellulose nanofibril network with lateral dimensions of 20–60 nm and lengths up to several micrometers. For the corresponding anionic powder grade, the fibrils carry carboxyl and hydroxyl surface groups, with an average width of approximately 50 nm and lengths in the range 0.5–80 µm. The repeat-unit composition is represented as [(C_6_H_10_O_5_)*_x_*(C_6_H_9_O_4_COONa)*_y_*]. In our previous characterization of the same TCNF material, the carboxylate and aldehyde contents were 1.4 mmol g^−1^ and 0.58 mmol g^−1^ of dry solids, respectively [4]. These values serve as the batch-relevant charge and oxidation descriptors. Deionized water containing 0.02 wt% sodium azide (NaN_3_) was used as solvent and biocide during preparation and storage. The solids content of the NRL dispersion was verified against the nominal value at the start of the campaign. Sodium azide is not included in the declared total solids content of the hydrogels, and its contribution would be at most 0.016 percentage points at the lowest solids content studied. As an electrolyte the azide contributes about 3 mmol per kg of aqueous phase, against about 12 to 61 mmol per kg of sodium counterions of the TCNF carboxylate groups across the formulations studied, so its sodium amounts to between about one twentieth and one quarter of that supplied by the fibril counterions. Its concentration was identical in every formulation, and its effect on colloidal stability was not measured.

The carbon additives were a graphene additive marketed as “graphene conductive additive” (GCA), graphene nanoplatelets (GNPs), multi-walled carbon nanotubes (MWCNTs), carboxylated multi-walled carbon nanotubes (MWCNT-COOH), and graphite flakes. The abbreviation GCA follows the supplier’s product name. No electrical measurements were performed in this work, and the name does not imply measured electrical functionality. GCA, GNP, MWCNT, and MWCNT-COOH were obtained from Cheap Tubes, graphite flakes from Alfa Aesar. Supplier-reported specifications: GCA, fully synthetic graphene nanoparticles, 98% carbon, 50-100 nm particle size, 10–30 graphene layers; GNP, chemically exfoliated natural-graphite nanoplatelets, 97% purity, approximately 1 µm lateral size, 8–30 nm thickness, specific surface area above 200 m^2^ g^−1^; MWCNT, outer diameter < 20 nm, inner diameter 4 nm, length 1–12 µm, purity > 99 wt%, ash 0 wt%, specific surface area approximately 250 m^2^ g^−1^; MWCNT-COOH, outer diameter < 20 nm, inner diameter 4 nm, length 1–15 µm, purity > 99 wt%, ash 0 wt%, COOH as the primary surface functionality. All additives were used as received. Supplier lot numbers were not recorded at the time of purchase and cannot be reconstructed, so the materials are identified here by supplier, grade and the specifications above, and the samples themselves by composition. The five additives differ simultaneously in aspect ratio, particle size, specific surface area, surface chemistry, and morphology, so “additive identity” in this work denotes the additive as supplied, not any single physicochemical variable (Section 2.5).

Throughout the manuscript, formulation labels give the total solids content followed by the dry-mass ratio of NRL, TCNF, and carbon additive. Thus 20%_1.0NRL-0.05TCNF-0.1GCA denotes 20 wt% total solids, calculated from the combined dry masses, with NRL:TCNF:CA = 1.0:0.05:0.10, where CA is the carbon additive of the sample.

The materials studied here are water-continuous physical gels in which all structural components, fibrils, latex particles, and carbon particles are colloidally dispersed, and in which the aqueous phase is the majority phase at the water contents studied (60–80 wt%). We use “hydrogel” for these materials because they are water-continuous, solid-like soft materials, and “colloidal gel” when the colloidal nature of the network-forming units is the relevant aspect. The systems differ from covalently crosslinked hydrogels in that the network arises from physical association of dispersed components, and from a plain latex dispersion in that they are elastic dominated at rest at all measured compositions (Section 2).

### 4.2. Hydrogel Preparation and Formulation Design

All preparation steps were performed under continuous ice-bath cooling. The 6 wt% TCNF stock dispersion was diluted with deionized water and mixed for 10 min at 400 rpm using an overhead stirrer with a PTFE-coated foldable-paddle impeller. The carbon additive was introduced gradually while the stirring rate was increased to 500 rpm. The TCNF/additive dispersion was homogenized by probe sonication (Hielscher UP200Ht with an S26d7 sonotrode of 7-mm tip diameter) for 15 min at 30 W with a 2-s-on/2-s-off pulse sequence, corresponding to 7.5 min of net sonication time per batch of approximately 50 mL, while stirring at 600 rpm. The 61 wt% prevulcanized NRL dispersion was then added slowly during continued mixing at 600 rpm for approximately 10 min, followed by final mixing for 30 min at 700 rpm. The same sonication and mixing protocol was applied to all formulations. Possible differences in the dispersion state achieved by the same energy input for different additives are addressed as a limitation in Section 2.5.

Formulations were organized into four sets (Table 1). Set 1 follows time evolution at a fixed component ratio and five total solids contents; Sets 2 and 3 vary the TCNF/GCA balance and the GCA loading at the late-storage comparison state; Set 4 compares the five carbon additives at fixed-base composition.

For Set 1, hydrogels were prepared at NRL:TCNF:GCA = 1.0:0.05:0.10 and total solids of 20, 25, 30, 35, and 40 wt%. For Set 2, hydrogels were prepared at 25 wt% total solids with NRL fixed at 1.0 and complementary TCNF/GCA ratios of 0.00/0.15, 0.03/0.12, 0.05/0.10, 0.07/0.08, 0.10/0.05, 0.12/0.03, and 0.15/0.00. The no-TCNF formulation was unstable during preparation and could not be measured. The lowest-TCNF formulation was close to the feasibility boundary. These observations delimit formulation feasibility, and quantitative interpretation is restricted to measured datasets. For Set 3, hydrogels were prepared at 25 wt% total solids with NRL:TCNF = 1.0:0.07 and GCA loadings of 0, 0.08, 0.10, 0.15, 0.20, and 0.25. For Set 4, hydrogels were prepared at 25 wt% total solids with NRL:TCNF:CA = 1.0:0.07:0.15, where CA was GCA, GNP, MWCNT, MWCNT-COOH, or graphite flakes. The mass of each component per 100 g of hydrogel, together with the masses of the as-received latex dispersion and of the TCNF stock that supply them, is given in Supplementary Table S11. Three formulations occur in two sets each and were prepared once and measured once, so the 22 entries of Table 1 correspond to 19 distinct preparations. Prepared hydrogels were stored in glass vials closed with gasket-sealed screw caps at ambient laboratory temperature. Sample mass was not monitored during storage, so evaporation losses are inferred from the sealing rather than measured, and neither pH nor electrical conductivity of the dispersions was measured.

### 4.3. Rheological Measurements

Rheological measurements were performed on an Anton Paar Physica MCR 301 rotational rheometer with a cross-hatched parallel-plate geometry (PP50/P2, 50 mm diameter) at a plate gap of 1.0 mm. The cross-hatched surfaces were chosen to suppress wall slip. Slip cannot be excluded entirely, and no systematic gap or surface variation was performed (Section 2.5). A glass cover minimized water evaporation. All measurements were conducted at 20 °C. The specified minimum torque of the instrument is 0.1 µN·m in oscillation and 0.02 µN·m with direct strain control, its torque resolution is 0.001 µN·m, and its maximum torque is 200 mN·m. Torque values at the lowest shear rates remained above the specified minimum, and the normal force stayed within ±1 N. Samples were taken from the storage vial with a spatula and applied to the lower plate without remixing, so that the structure developed during storage was preserved. After the gap was set and the edge was trimmed, the sample rested for approximately 2 min before the first test. On each loading the frequency sweep was run first and took approximately 20 min, and the flow curve followed after a further 1 to 2 min, so that the oscillatory and steady-shear data reported for a given formulation and storage time were measured on the same loading. One batch was prepared per formulation and one loading was measured per formulation and storage time, so the reported curves are single measurements and not averages of replicates. Reproducibility was assessed at the start of the campaign, on one to two samples only, by measuring three separate loadings taken from the same batch.

The linear viscoelastic (LVE) range was determined by strain amplitude sweeps at 1 Hz, from 0.001 to 1000% strain at five points per decade. The constant strain amplitude of 0.1% used in all frequency sweeps lies within the LVE range so determined, at least seven times below the narrowest limit measured (Figure S2 and Table S5). Frequency sweeps were carried out from 100 to 0.01 Hz at 21 logarithmically spaced points (five per decade) in descending order. Data above 6.31 Hz were excluded from analysis and from the figures because the raw moduli become non-monotonic there, which indicates instrument inertia effects. The analysis window is therefore 0.01 to 6.31 Hz. The storage modulus *G′*, the loss modulus *G″*, and the damping factor tan *δ* = *G″*/*G′* were recorded.

Steady flow behavior was determined with a logarithmic controlled shear-rate ramp from 1 × 10^−5^ to 1000 s^−1^ at five points per decade, that is 41 points per curve, with the measuring time per point decreasing logarithmically from 120 s to 1 s and no steady-state criterion applied. The measured shear rates spanned approximately 4 × 10^−6^ to 1000 s^−1^. At the lowest set rates the control loop does not reach the set value within the allotted measuring time, so the measured rather than the set shear rate is reported, and its values are not identical between samples. During data processing, points with shear rates below 1 × 10^−5^ s^−1^ and the first three points of each curve were discarded. The rule was fixed and applied identically to every curve rather than decided per curve. Its basis is that those points carry less than 0.4% of accumulated strain in total (Supplementary Table S7), so the material has barely been deformed, and that they contain every instance in the dataset of a measured shear rate that is zero or negative, which is the signature of the control loop not yet having engaged. The retained data cover shear rates from approximately 5 × 10^−5^ to 1000 s^−1^. Apparent viscosity *η* and shear stress *σ* were recorded as functions of the measured shear rate. Because the measuring time per point is fixed rather than set by a steady-state criterion, the strain accumulated at the lowest rates is small. In the first retained point it is 0.2 to 0.3%, and the cumulative strain reaches 2%, which is of the order of the linear-viscoelastic limit of these materials, only at a median shear rate (Supplementary Table S7) of 1.6 × 10^−4^ s^−1^. The low-shear plateau of these curves is therefore an apparent, protocol-dependent viscosity, and the low-shear Cross parameter is interpreted in that sense throughout.

### 4.4. Data Processing and Rheological Modeling

Model fits are used as phenomenological descriptions within the measured windows, not for extrapolation or mechanism assignment.

Steady-shear viscosity data were represented by the Cross model:

(1) $$\eta (\dot{\gamma })=\eta_{\infty }+\frac{\eta_{0}-\eta_{\infty }}{1+(\lambda \dot{\gamma }{)}^{n}}\;$$

where *η* is the apparent viscosity, $\dot{\gamma }$ the shear rate, *η*_0_ and *η*_∞_ the zero-shear and infinite-shear viscosity limits of the model, *λ* a characteristic time constant associated with the onset of shear thinning, and *n* the shear-thinning exponent. Because the flow protocol does not reach a steady state at the lowest rates (Section 4.3), the fitted *η*_0_ is reported throughout as an apparent low-shear viscosity. Fits minimized squared residuals of log_10_ *η* with the constraint *n* ≤ 1. For 97 of 109 fitted curves the optimal exponent reached this bound, so the fitted description effectively has three free parameters for most samples. Fitted parameters, their 95% confidence intervals, and the bound status are listed in Supplementary Table S1, and Supplementary Table S14 collects those together with *G′*, *G″,* and tan *δ* at 1 Hz for every formulation and storage time in a single table. The confidence interval of *η*_0_ is typically ±15% (maximum ±39%), and *R*^2^ of log_10_ *η* exceeds 0.993 for all fits. A high coefficient of determination does not exclude systematic deviation, so the residuals of all 109 fits are shown in Supplementary Figure S4. They are structured rather than random: the median residual reaches +0.10 in log_10_ *η* near 0.04 s^−1^ and −0.11 near 2.5 s^−1^, which is +26% and −23% in viscosity. Below 1 × 10^−3^ s^−1^, the region from which the low-shear parameter is taken, the median residual stays within 5%. The Cross function is therefore used as a comparison device and not as a constitutive description, and no conclusion rests on its shape in the shear-thinning region.

Complex viscosity was calculated as

(2) $$\left|\eta^{*}(\omega )\right|=\frac{\sqrt{G^{\prime }(\omega {)}^{2}+G^{\prime \prime }(\omega {)}^{2}}}{\omega }\;\;$$

with *ω* = 2πf from the exported frequency *f*. For Cox–Merz comparisons, steady-shear viscosity *η*($\dot{\gamma }$) and |η*(*ω*)| were compared at $\dot{\gamma }$ = *ω*. The deviation was quantified as *Δ*(*ω*) = log_10_[|η*(*ω*)|/*η*($\dot{\gamma }$ = *ω*)], interpolated on a common logarithmic grid over the overlapping range, and summarized per formulation by its mean absolute value and by the mean viscosity ratio 10mean(*Δ*) (Supplementary Table S3). The overlapping range is the same for every formulation, 2.80 decades, and contains 14 measured steady-shear points and 15 measured oscillatory points (Supplementary Table S8). The two measurements of a given formulation and storage time were made on the same loading, one after the other (Section 4.3), so the comparison is between two protocols applied to one specimen. The Cox–Merz representation is used as a diagnostic only and does not imply rule validity [10].

Oscillatory data were represented by a generalized Maxwell (Maxwell–Wiechert) model (GMM):

(3) $$G^{\prime }(\omega )=G_{e}+\sum_{i=1}^{N}\frac{g_{i}\;(\omega \tau_{i}{)}^{2}}{1+(\omega \tau_{i}{)}^{2}}\;\;$$

(4) $$G^{\prime \prime }(\omega )=\sum_{i=1}^{N}\frac{g_{i}\;\omega \tau_{i}}{1+(\omega \tau_{i}{)}^{2}}\;\;$$

where *G_e_* is the equilibrium elastic modulus and g*_i_*, *τ_i_* are the modulus and relaxation time of the i-th mode. Relaxation times were fixed a priori at *τ_i_* = 0.001, 0.01, 0.1, 1, 10, and 100 s, and *G_e_* with the modal amplitudes was fitted simultaneously to *G′* and *G″* by minimizing the dimensionless objective below. The residuals of *G′* and *G″* are made relative rather than absolute so that neither modulus dominates the fit, which matters here because *G′* exceeds *G″* by close to an order of magnitude throughout the window.

(5) $$\Phi =\sum_{i=1}^{M}\left[{\left(\frac{G_{\mathrm{cal},i}^{\prime }}{G_{i}^{\prime }}-1\right)}^{2}+{\left(\frac{G_{\mathrm{cal},i}^{\prime \prime }}{G_{i}^{\prime \prime }}-1\right)}^{2}\right]\;\;$$

The objective is over the *M* frequency points of the analysis window. The fixed logarithmic grid is a reproducibility and comparison device for this ill-posed inverse problem, not evidence for six discrete physical-relaxation modes [7,38]. Its robustness was tested explicitly: refitting with the grid shifted by factors of 3 and 1/3 changes *G_e_* by a median of 57% and 9%, respectively, while the fitted curves remain visually indistinguishable, and the longest relaxation time exceeds the longest experimentally probed time (about 16 s at 0.01 Hz), so individual GMM parameters are not identifiable from these data. Consequently, no conclusion in this work rests on the value of any individual GMM parameter. The fits serve only as smooth representations for comparing spectra. Per-modulus goodness of fit, residual plots, and the grid-sensitivity analysis are given in Supplementary Table S2 and Supplementary Figure S1. Supplementary Figure S5 shows the residuals of the storage modulus on a relative and on an absolute scale for every formulation at its late-storage state. They are systematic rather than random: the fit underpredicts the storage modulus by a median of 9% at 0.01 Hz, the deviation changes sign near 0.09 Hz, and the pattern is nearly the same in every formulation, which is a further reason not to read the individual modes as physical.

## Figures and Tables

**Figure 1:**
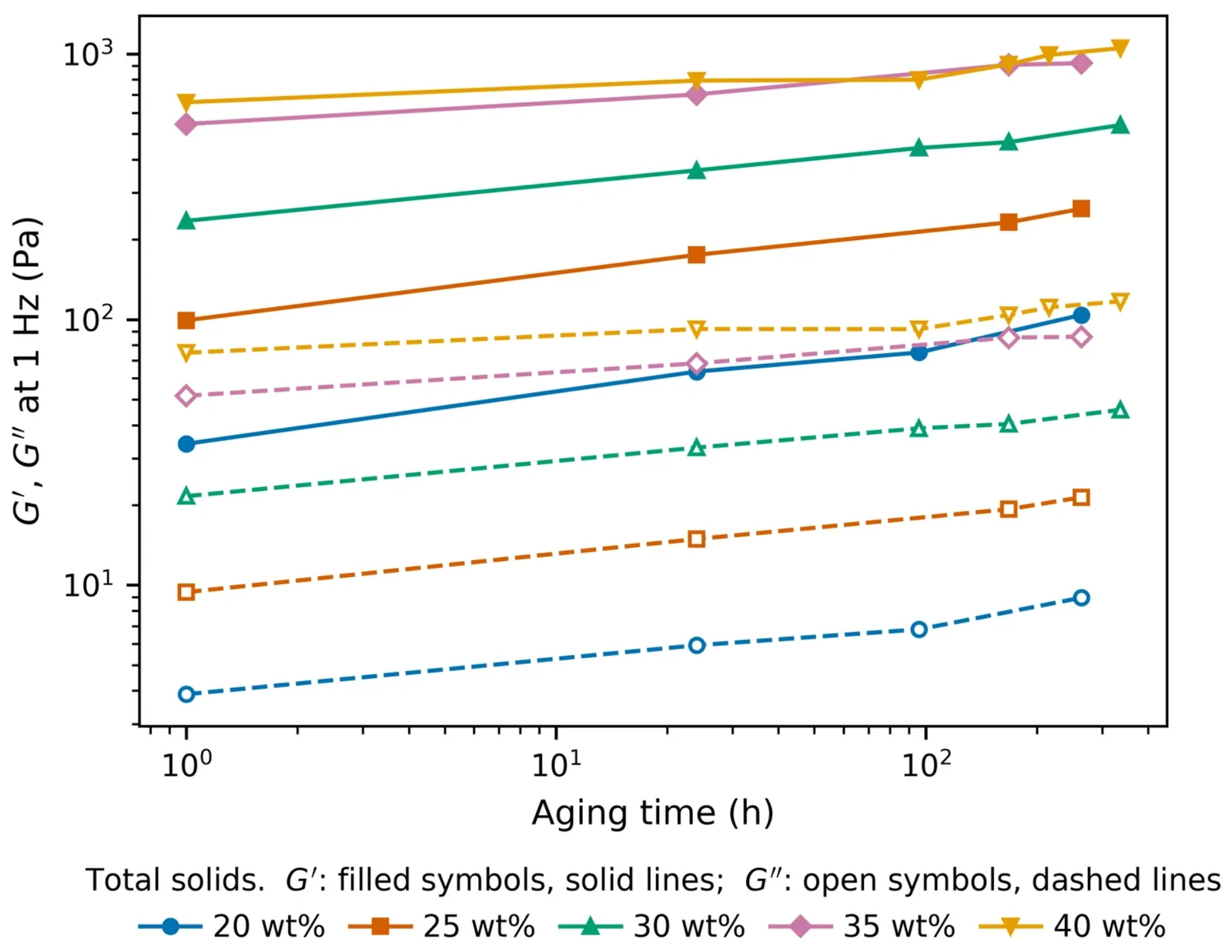
Storage modulus *G′* (filled symbols, solid lines) and loss modulus *G″* (open symbols, dashed lines) at 1 Hz as functions of aging time for the Set 1 formulations (20-40 wt% total solids). Symbols denote experimental data.

**Figure 2:**
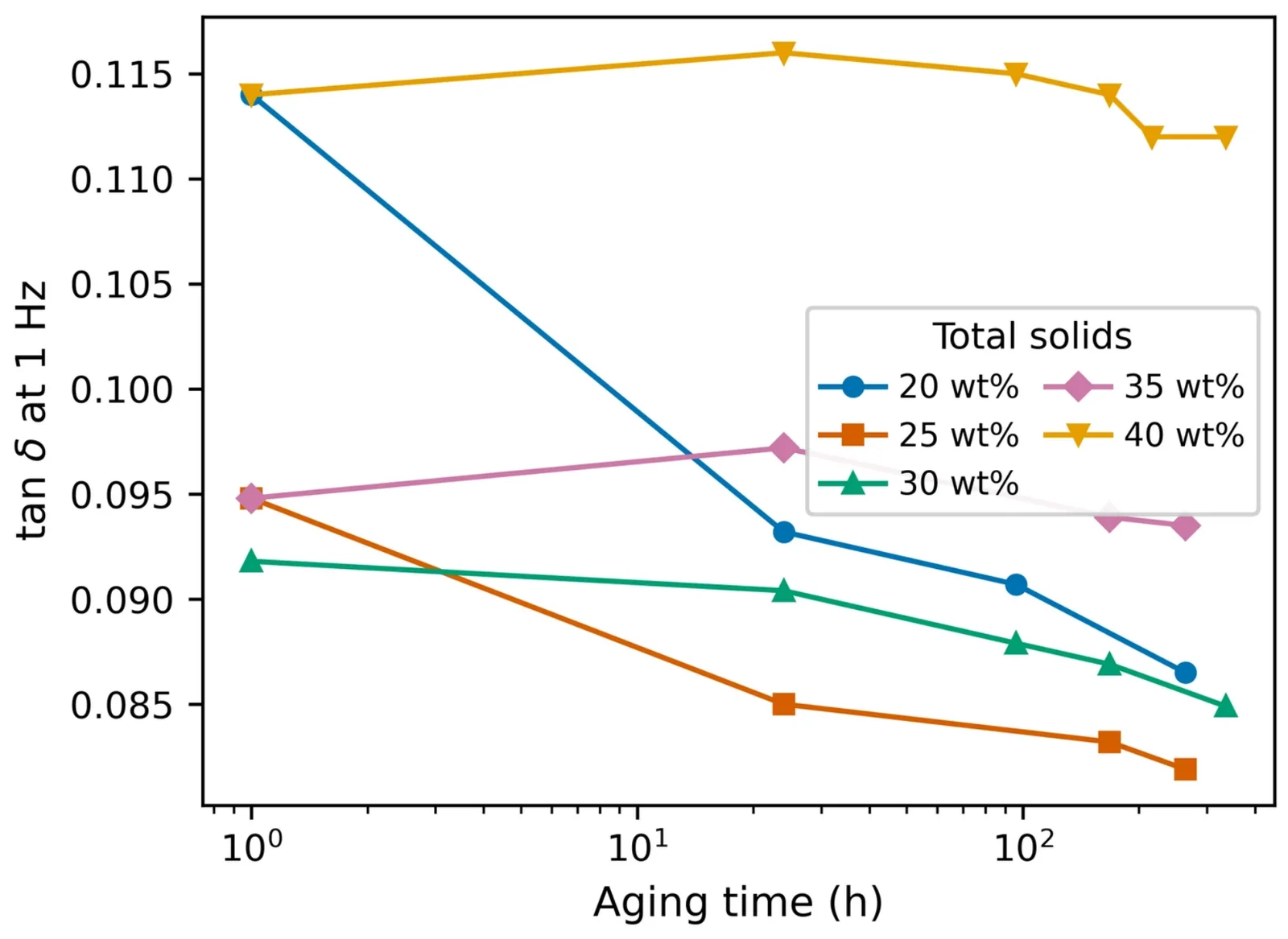
Damping factor tan *δ* at 1 Hz as a function of aging time for the Set 1 formulations.

**Figure 3:**
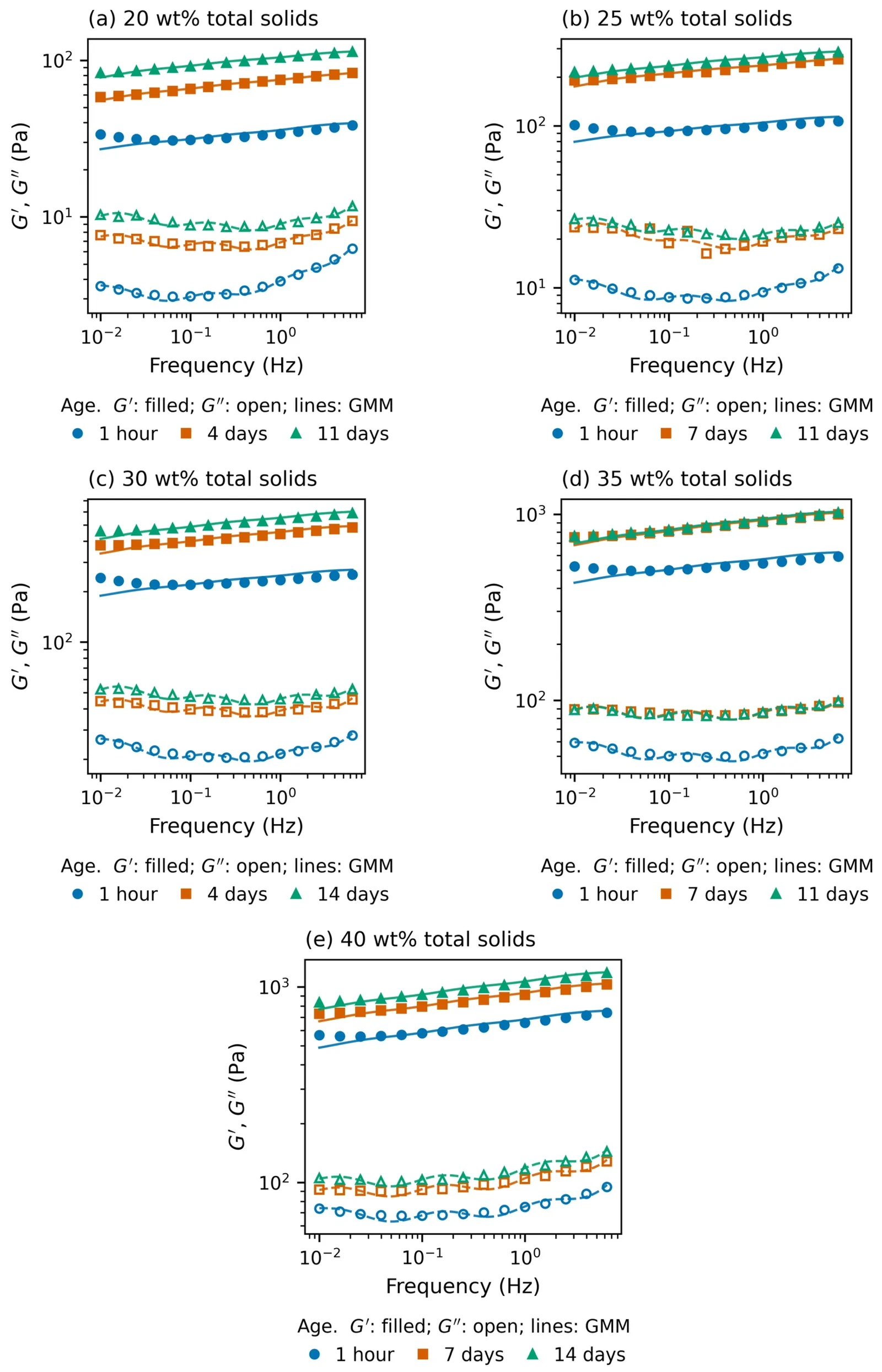
Frequency dependence of *G′* (filled symbols) and *G″* (open symbols) for Set 1 hydrogels at three aging times per panel: (**a**) 20, (**b**) 25, (**c**) 30, (**d**) 35, (**e**) 40 wt% total solids. Lines are generalized Maxwell (GMM) descriptions of *G′* (solid) and *G″* (dashed). In panel d the 7-day and 11-day spectra nearly coincide because aging has largely saturated at this solids content.

**Figure 4:**
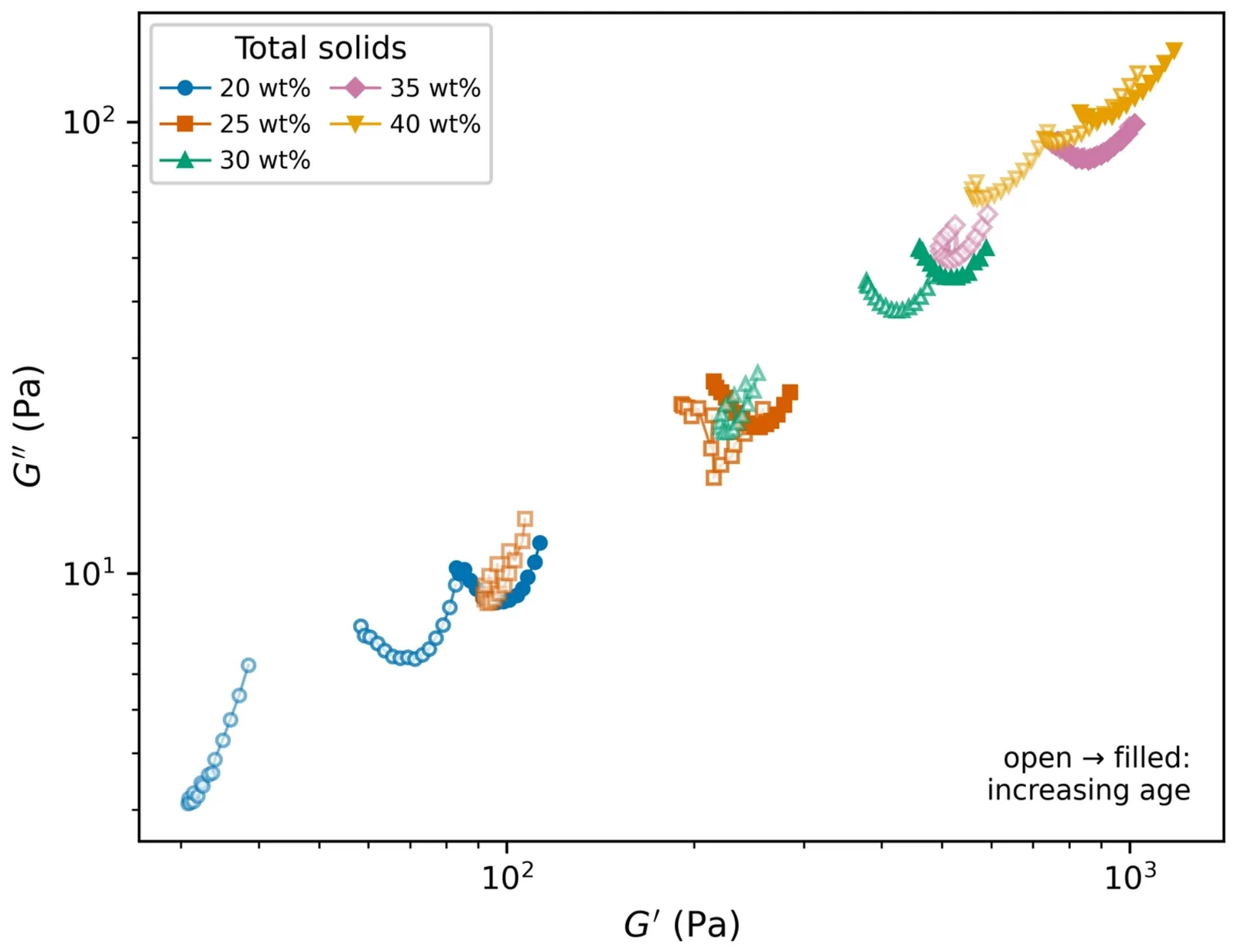
Cole–Cole representation (*G″* versus *G′*) of the Set 1 oscillatory data at three aging times per formulation. Symbol fill goes from open (early age) to filled (late age).

**Figure 5:**
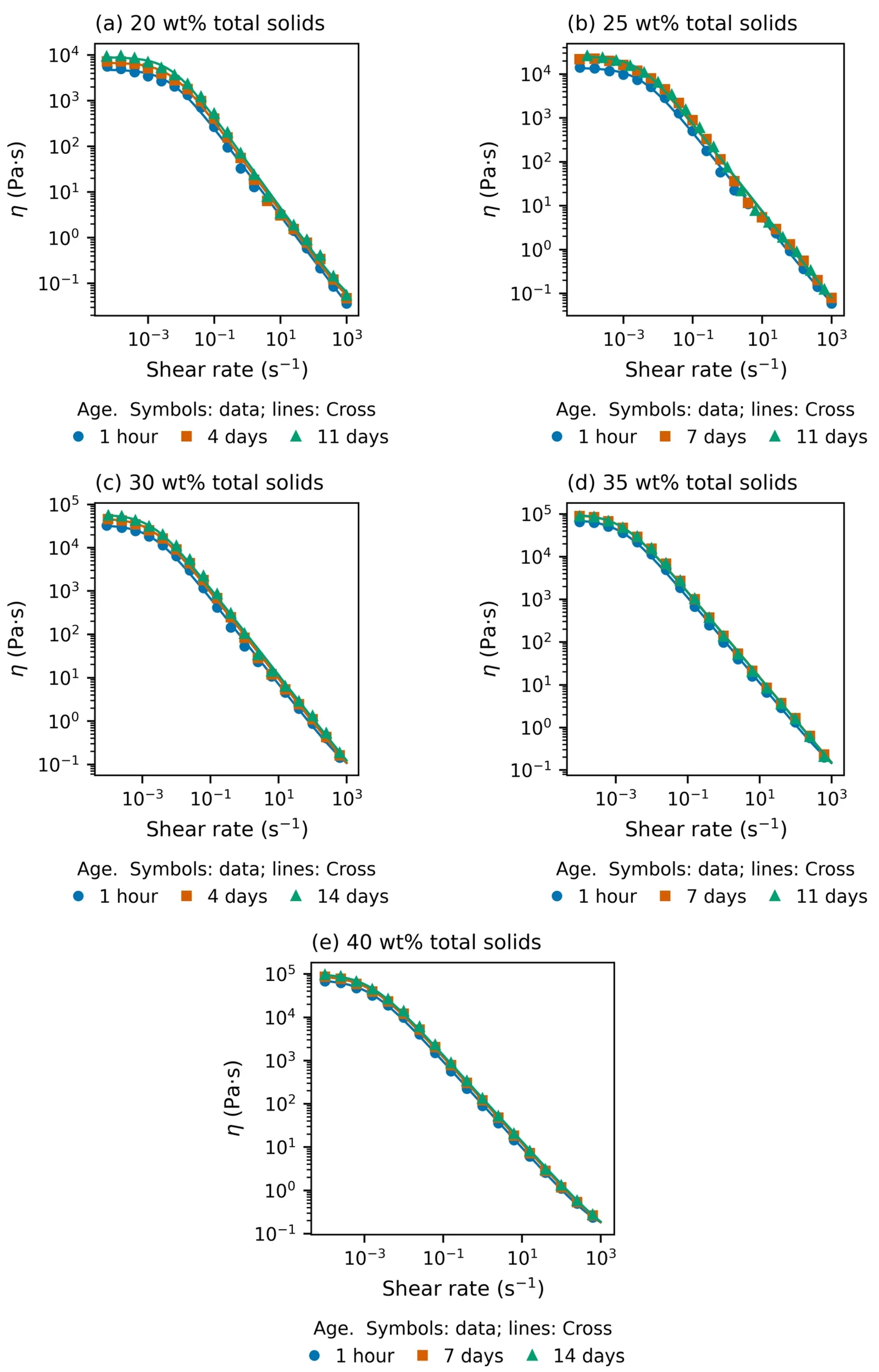
Steady-shear viscosity (symbols, every second point shown) and Cross-model fits (lines) for Set 1 hydrogels at three aging times per panel: (**a**) 20, (**b**) 25, (**c**) 30, (**d**) 35, (**e**) 40 wt% total solids. The fits represent the measured shear-thinning regime; larger deviations are possible at the lowest accessible shear rates in highly aged samples.

**Figure 6:**
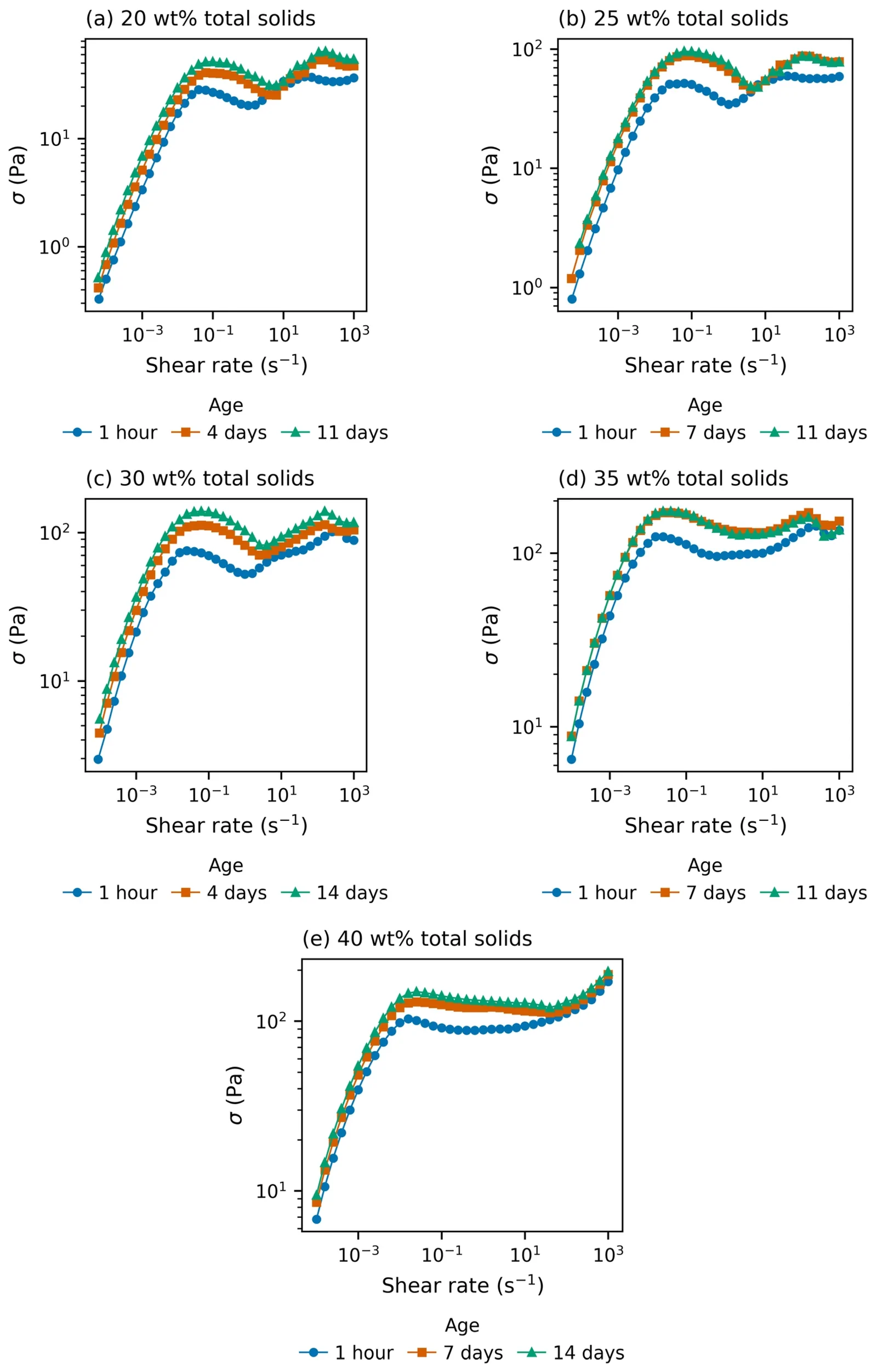
Shear stress as a function of measured shear rate for Set 1 hydrogels at three aging times per panel (controlled shear-rate protocol). Note the weak non-monotonic features at intermediate shear rates.

**Figure 7:**
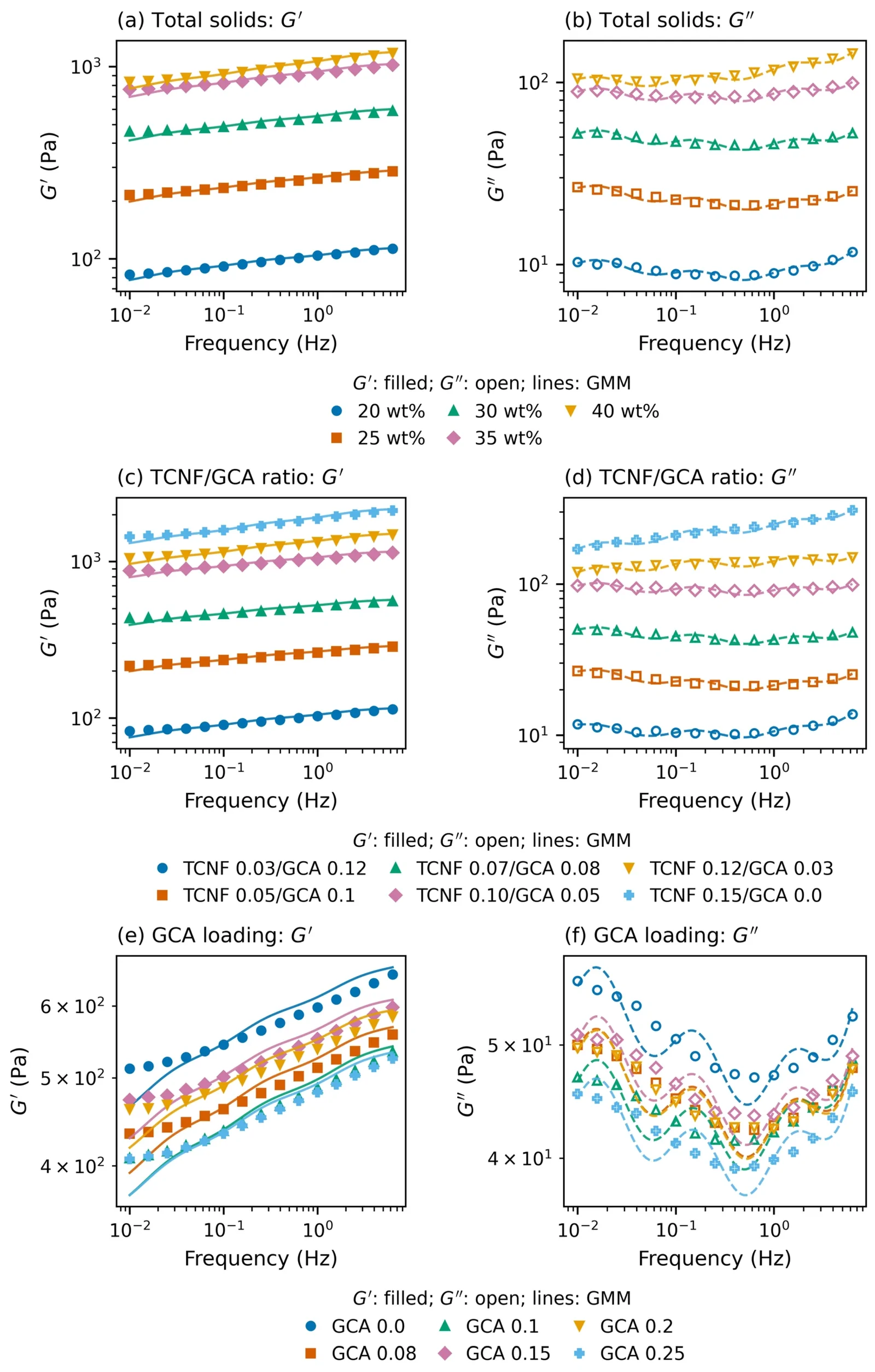
Frequency dependence of *G′* (**a**,**c**,**e**; filled symbols) and *G″* (**b**,**d**,**f**; open symbols) at the late-storage states for the composition series: (**a**,**b**) total solids (Set 1); (**c**,**d**) TCNF/GCA ratio (Set 2); (**e**,**f**) GCA loading (Set 3). Lines are GMM descriptions (solid: *G′*, dashed: *G″*). Note the small spread in panels (**e**,**f**). The late-storage ages are 11 days at 20, 25, and 35 wt% and 14 days at 30 and 40 wt% in Set 1, 11 to 14 days in Set 2, and 14 to 35 days in Set 3 (Table 1).

**Figure 8:**
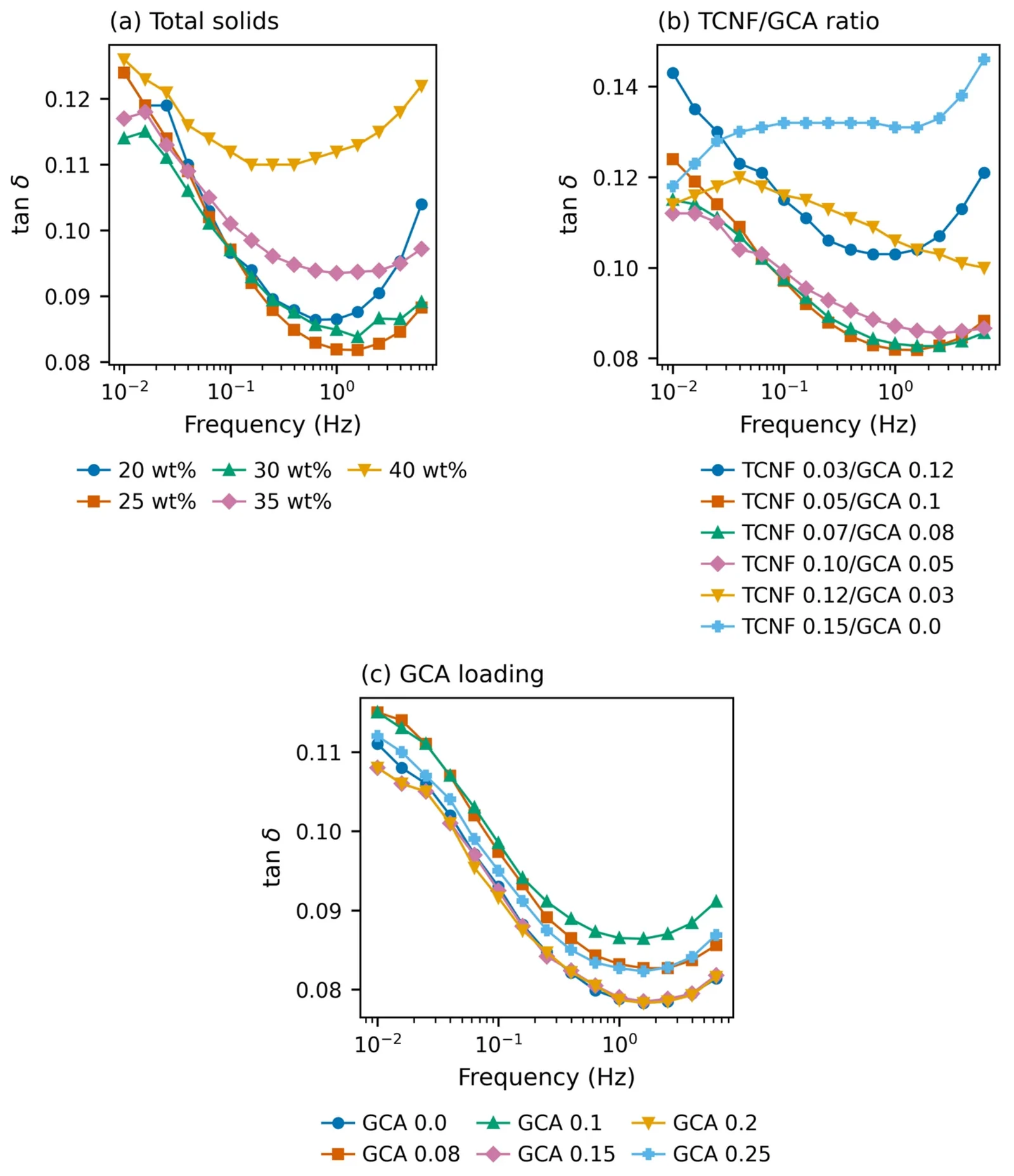
Damping factor tan *δ* versus frequency at the late-storage states: (**a**) Set 1, (**b**) Set 2, (**c**) Set 3. The late-storage ages are 11 days at 20, 25 and 35 wt% and 14 days at 30 and 40 wt% in Set 1, 11 to 14 days in Set 2, and 14 to 35 days in Set 3 (Table 1).

**Figure 9:**
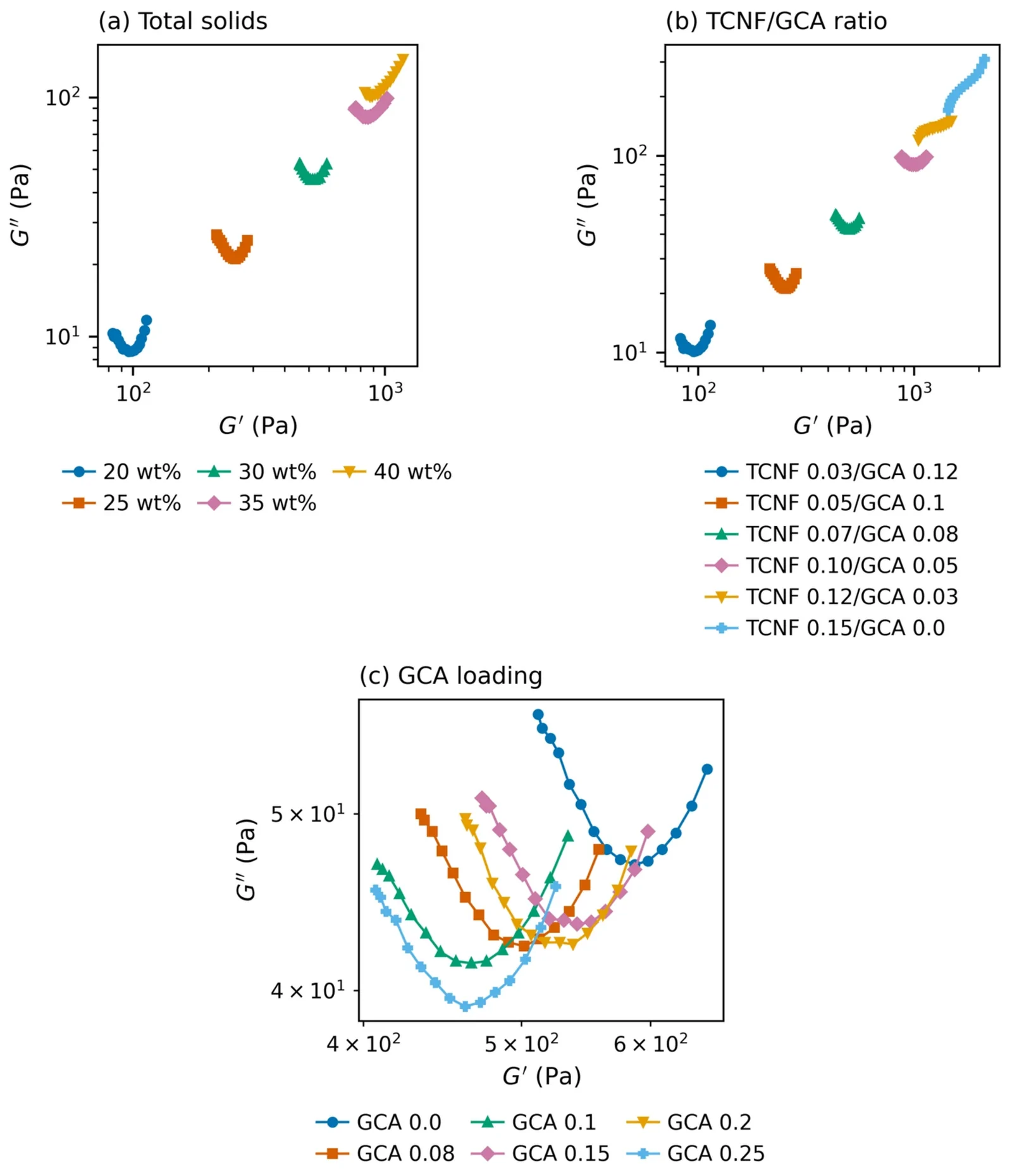
Cole–Cole representations at the late-storage states: (**a**) Set 1, (**b**) Set 2, (**c**) Set 3. The late-storage ages are 11 days at 20, 25 and 35 wt% and 14 days at 30 and 40 wt% in Set 1, 11 to 14 days in Set 2, and 14 to 35 days in Set 3 (Table 1).

**Figure 10:**
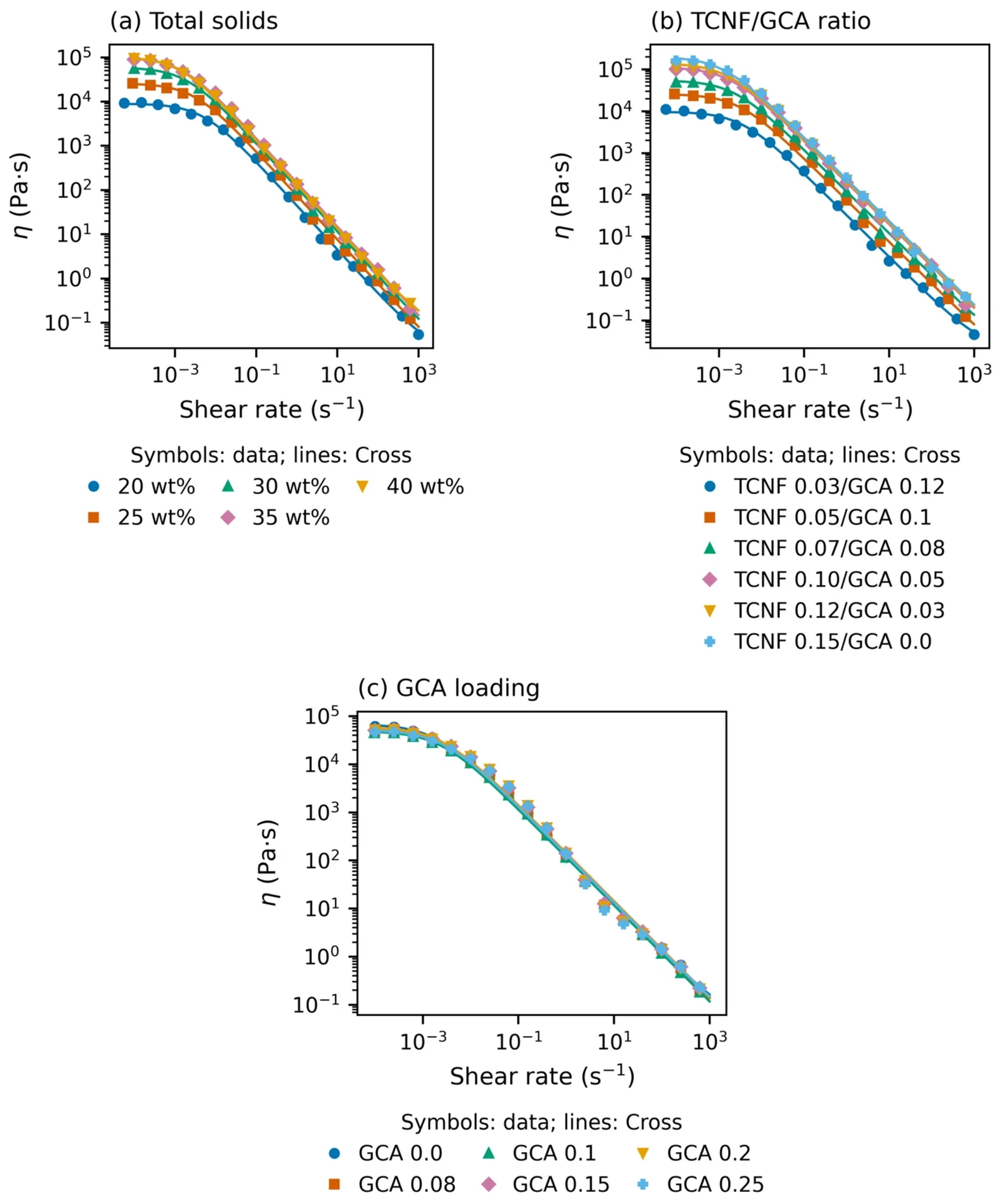
Steady-shear viscosity (symbols, every second point) with Cross fits (lines) at the late-storage states: (**a**) Set 1, (**b**) Set 2, (**c**) Set 3. The late-storage ages are 11 days at 20, 25, and 35 wt% and 14 days at 30 and 40 wt% in Set 1, 11 to 14 days in Set 2, and 14 to 35 days in Set 3 (Table 1).

**Figure 11:**
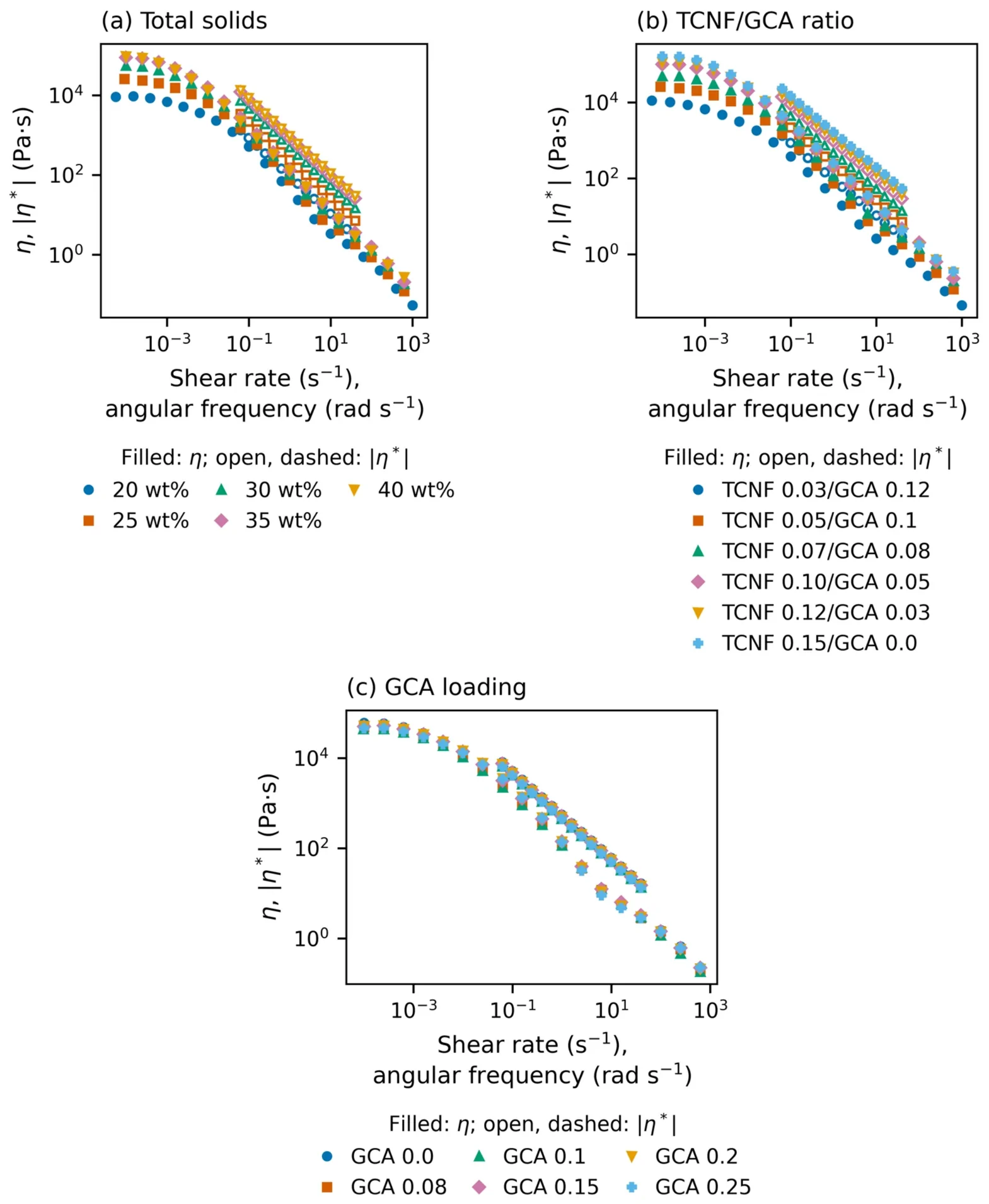
Cox–Merz comparison at the late-storage states: steady-shear viscosity *η*($\dot{\gamma }$) (filled symbols) and complex viscosity magnitude |η*|(*ω*) (open symbols, dashed) versus shear rate and angular frequency: (**a**) Set 1, (**b**) Set 2, (**c**) Set 3. The late-storage ages are 11 days at 20, 25, and 35 wt% and 14 days at 30 and 40 wt% in Set 1, 11 to 14 days in Set 2, and 14 to 35 days in Set 3 (Table 1).

**Figure 12:**
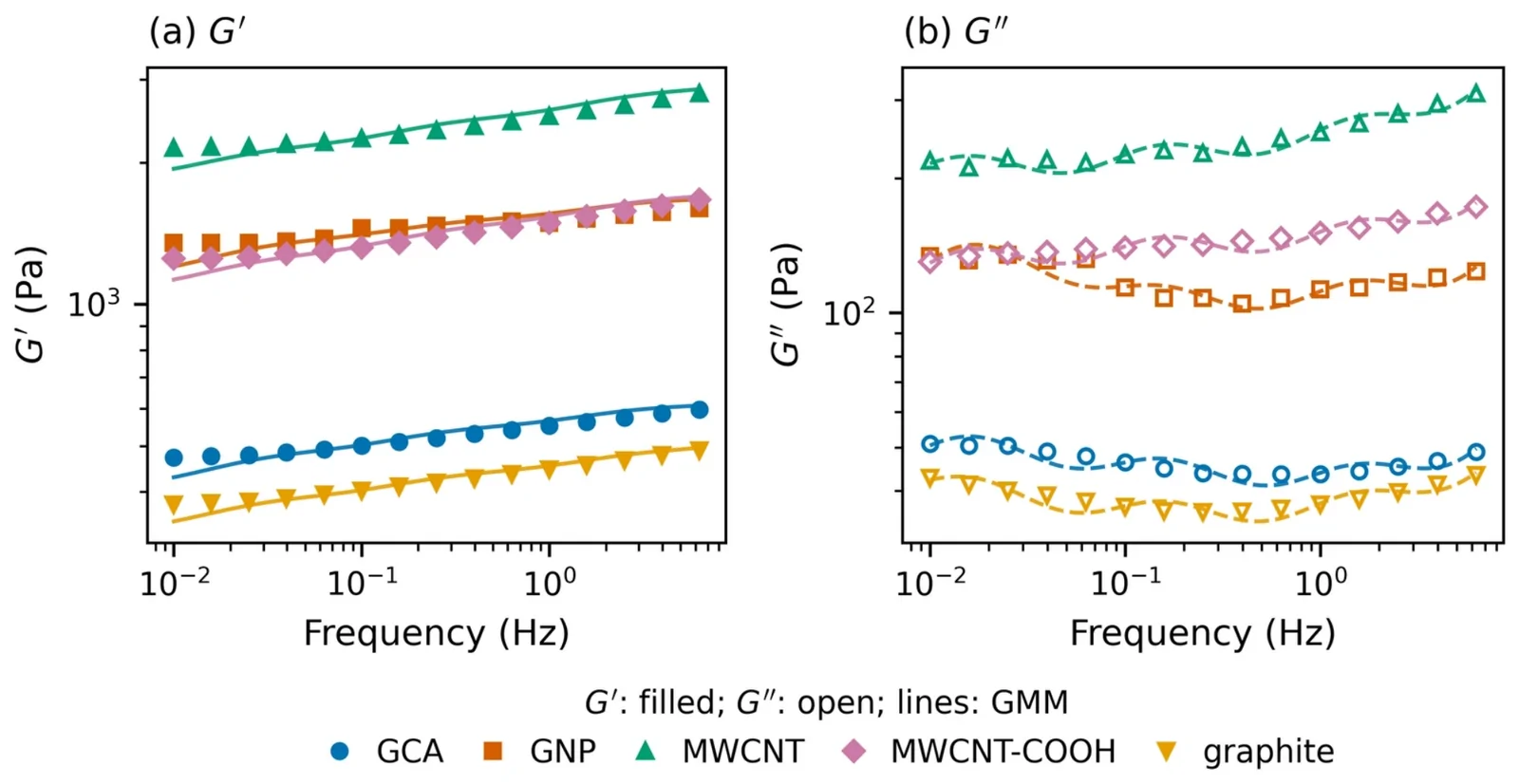
Frequency dependence of (**a**) *G′* and (**b**) *G″* for Set 4 hydrogels (additive series) at the late-storage states. Symbols: data; lines: GMM descriptions. The late-storage ages are 35 days for GCA, 15 days for GNP and MWCNT, and 13 days for MWCNT-COOH and graphite (Table 1).

**Figure 13:**
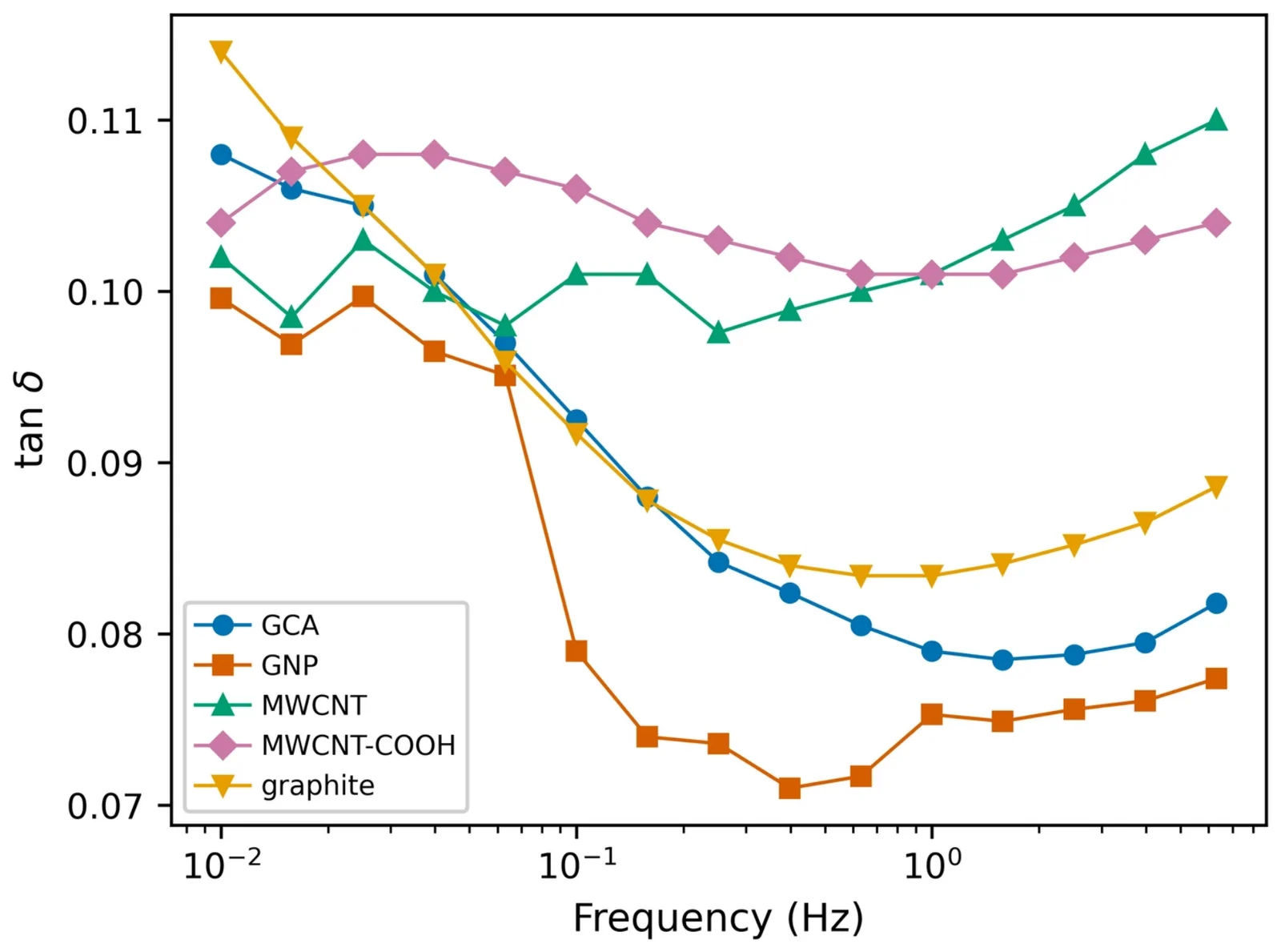
Damping factor tan *δ* versus frequency for Set 4 hydrogels. The late-storage ages are 35 days for GCA, 15 days for GNP and MWCNT, and 13 days for MWCNT-COOH and graphite (Table 1).

**Figure 14:**
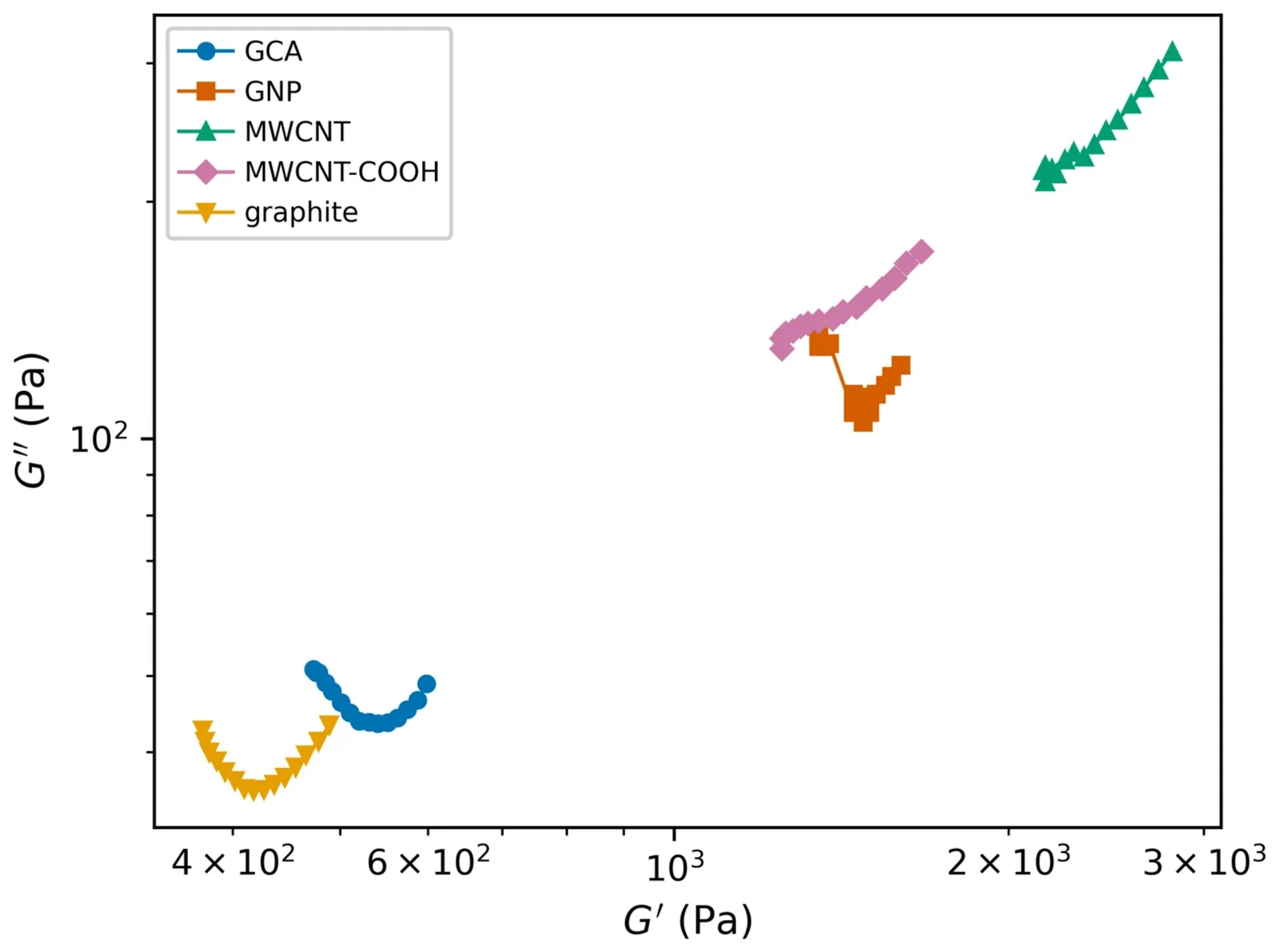
Cole–Cole representation for Set 4 hydrogels. The late-storage ages are 35 days for GCA, 15 days for GNP and MWCNT, and 13 days for MWCNT-COOH and graphite (Table 1).

**Figure 15:**
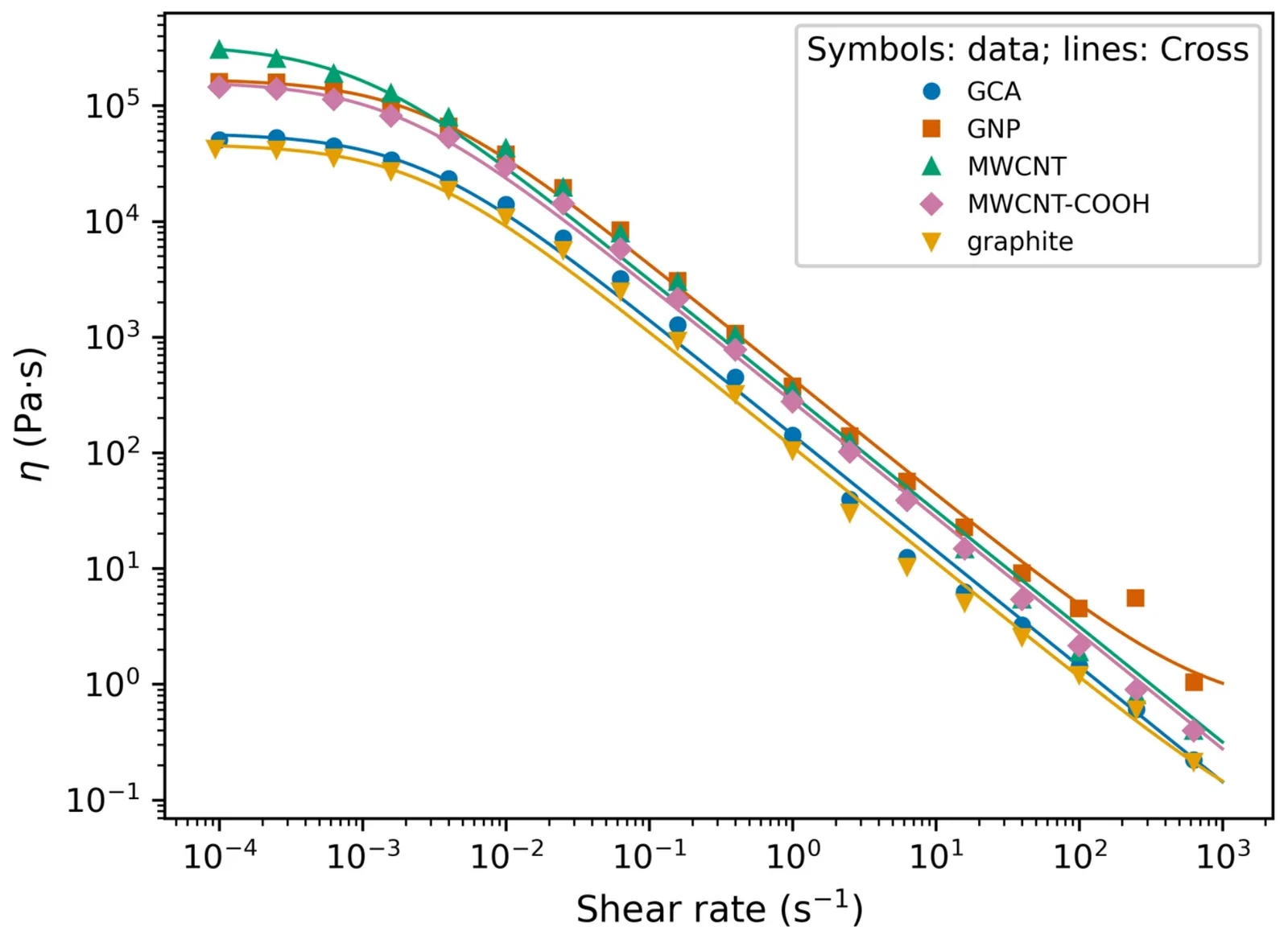
Steady-shear viscosity (symbols) and Cross fits (lines) for Set 4 hydrogels. In the GNP sample at 15 days, coagulates formed during the measurement and material was expelled at the plate edge at the highest rates, so the record of that curve above about 100 s^−1^ is not interpreted. The late-storage ages are 35 days for GCA, 15 days for GNP and MWCNT, and 13 days for MWCNT-COOH and graphite (Table 1).

**Figure 16:**
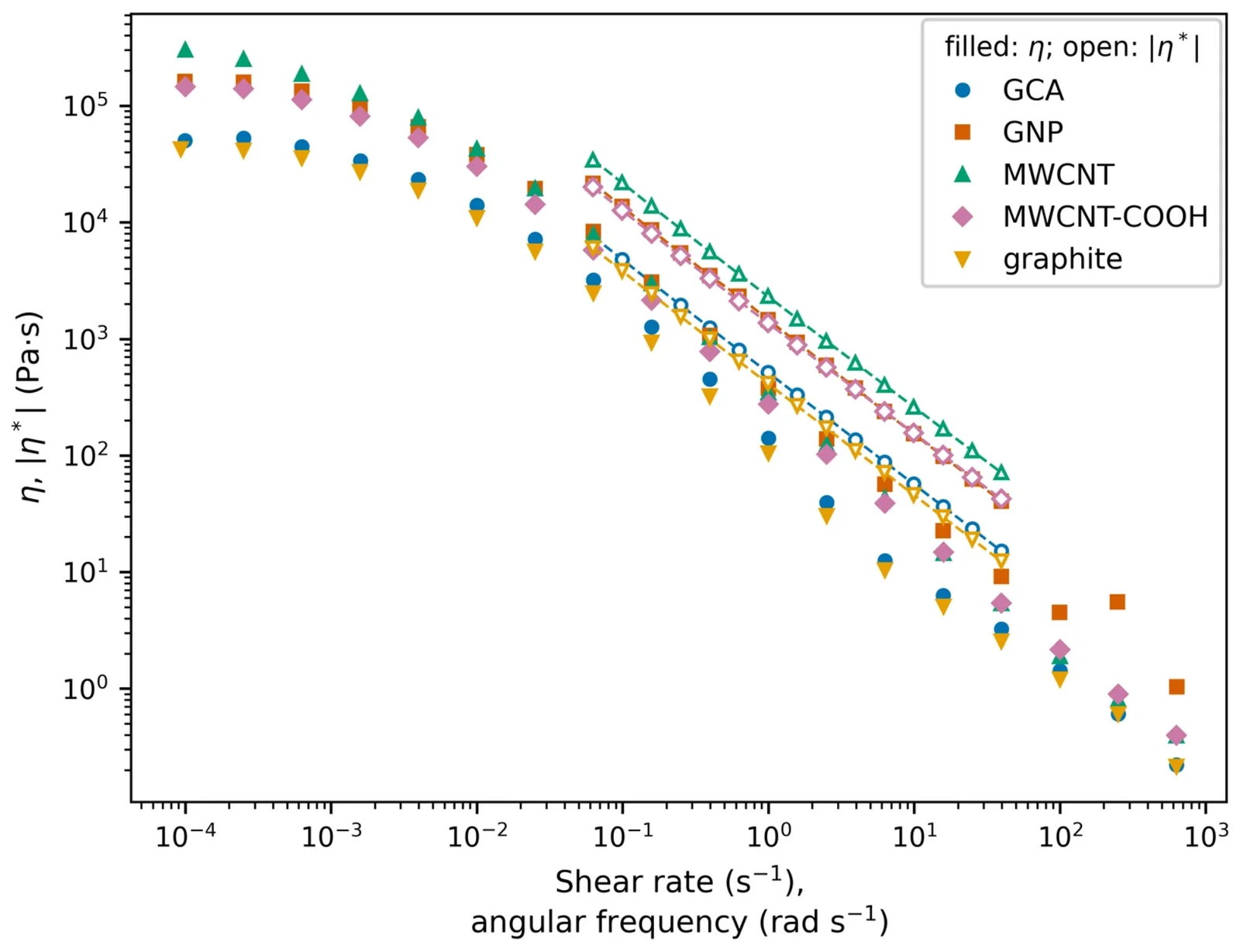
Cox–Merz comparison for Set 4 hydrogels: *η*($\dot{\gamma }$) (filled) and |η*|(*ω*) (open, dashed). The late-storage ages are 35 days for GCA, 15 days for GNP and MWCNT, and 13 days for MWCNT-COOH and graphite (Table 1).

**Table 1:** Formulations of sets 1-4: total solids content, dry-mass ratio NRL:TCNF:CA, carbon additive, number of measured aging times, and the aging time of the late-storage comparison state used in Section 2.2 and Section 2.3. NRL: natural rubber latex; TCNFs: TEMPO-oxidized cellulose nanofibrils; CA: carbon additive; GCA: graphene conductive additive; GNP: graphene nanoplatelets; MWCNT: multi-walled carbon nanotubes; MWCNT-COOH: carboxylated multi-walled carbon nanotubes. Rows sharing a superscript letter (^a^, ^b^, ^c^) are the same physical preparation, measured once and used in both sets, so the 22 entries correspond to 19 distinct preparations.

Set	Total Solids (wt%)	NRL:TCNF:CA	Carbon Additive	Aging Times Measured	Late-Storage Age (d)
1	20	1.0:0.05:0.10	GCA	4	11
1	25	1.0:0.05:0.10 ^a^	GCA	4	11
1	30	1.0:0.05:0.10	GCA	5	14
1	35	1.0:0.05:0.10	GCA	4	11
1	40	1.0:0.05:0.10	GCA	6	14
2	25	1.0:0.03:0.12	GCA	5	13
2	25	1.0:0.05:0.10 ^a^	GCA	4	11
2	25	1.0:0.07:0.08 ^b^	GCA	5	14
2	25	1.0:0.10:0.05	GCA	5	13
2	25	1.0:0.12:0.03	GCA	5	14
2	25	1.0:0.15:0.00	none	5	14
3	25	1.0:0.07:0.00	none	5	29
3	25	1.0:0.07:0.08 ^b^	GCA	5	14
3	25	1.0:0.07:0.10	GCA	5	29
3	25	1.0:0.07:0.15 ^c^	GCA	5	35
3	25	1.0:0.07:0.20	GCA	5	35
3	25	1.0:0.07:0.25	GCA	5	35
4	25	1.0:0.07:0.15 ^c^	GCA	5	35
4	25	1.0:0.07:0.15	GNP	6	15
4	25	1.0:0.07:0.15	MWCNT	6	15
4	25	1.0:0.07:0.15	MWCNT-COOH	5	13
4	25	1.0:0.07:0.15	graphite	5	13

## Data Availability

The data presented in this study are available on request from the corresponding author.

## Supplementary Materials

The following supporting information can be downloaded at: https://www.mdpi.com/article/10.3390/gels12100877/s1: Figure S1: GMM residuals at the late-storage states; Figure S2: Strain amplitude sweeps; Figure S3: Set 4 against storage time; Figure S4: Residuals of the Cross fits; Figure S5: Residuals of the generalized Maxwell fits on two scales; Figure S6: What the Cox–Merz deviation tracks; Table S1: Cross-model fits with 95% confidence intervals; Table S2: GMM fits: per-modulus goodness of fit and grid sensitivity; Table S3: Cox–Merz deviation metric at the late-storage states; Table S4: Late-storage comparison states and residual aging drift; Table S5: Strain-amplitude sweeps and the linear viscoelastic limits; Table S6: Cross fits with the shear-thinning exponent unconstrained; Table S7: Strain accumulated in the flow protocol; Table S8: Overlapping range of the Cox–Merz comparison; Table S9: Rank agreement between the oscillatory and steady-shear orderings; Table S10: Regression statistics for the aging trend; Table S11: Component masses per 100 g of hydrogel; Table S12: Bootstrap interval for the low-shear Cross parameter; Table S13: The additive comparison at matched storage times; Table S14: All measured and fitted quantities, per formulation and storage time.
