# Supplementary material for: Physical Aging, Composition, and Carbon-Additive Identity as Separable Rheological Controls in Colloidal-Cellulose Nanofibril–Natural-Rubber-Latex Hydrogels
Source: Gels. 2026 Sep 28;12(10):877. doi: 10.3390/gels12100877 (PMC13704036; doi:10.3390/gels12100877)

Supplementary material

Physical aging, composition, and carbon-additive identity as separable rheological controls in colloidal-cellulose nanofibril–natural-rubber-latex hydrogels

Branko Alič, Urška Šebenik, Matjaž Krajnc

Table S1. Cross-model fits with 95% confidence intervals.

Model: $\eta(\dot{\gamma}) = \eta\infty + (\eta 0 - \eta\infty)/(1 + (\lambda\dot{\gamma})^n)$, fitted on log10 η; λ is reported as k_s. n was constrained to $n \leq 1$.

Set	Formulation	Age	η0 ± CI95 (Pa·s)	λ (s)	n	R2(log10 η)
1st_set	20%_1.0NRL-0.05TCNF-0.1GCA	1 hour	4.77e+03 ± 571	211	0.963	0.999
1st_set	20%_1.0NRL-0.05TCNF-0.1GCA	24 hours (1 day)	6.08e+03 ± 785	215	0.975	0.998
1st_set	20%_1.0NRL-0.05TCNF-0.1GCA	4 days	6.76e+03 ± 915	195	0.99	0.998
1st_set	20%_1.0NRL-0.05TCNF-0.1GCA	11 days	8.9e+03 ± 1.19e+03	198	1 (at bound)	0.998
1st_set	25%_1.0NRL-0.05TCNF-0.1GCA	1 hour	1.38e4 ± 1.6e+03	304	0.988	0.999
1st_set	25%_1.0NRL-0.05TCNF-0.1GCA	24 hours (1 day)	1.81e4 ± 2.13e+03	303	0.997	0.999
1st_set	25%_1.0NRL-0.05TCNF-0.1GCA	7 days	2.24e4 ± 3.3e+03	310	1 (at bound)	0.998

Set	Formulation	Age	η0 ± CI95 (Pa·s)	λ (s)	n	R2(log10 η)
1st_set	25%_1.0NRL-0.05TCNF-0.1GCA	11 days	2.55e4 ± 4.34e+03	336	1 (at bound)	0.998
1st_set	30%_1.0NRL-0.05TCNF-0.1GCA	1 hour	3.33e4 ± 4.54e+03	503	0.992	0.999
1st_set	30%_1.0NRL-0.05TCNF-0.1GCA	24 hours (1 day)	3.75e4 ± 3.09e+03	443	1 (at bound)	1
1st_set	30%_1.0NRL-0.05TCNF-0.1GCA	4 days	4.73e4 ± 6.52e+03	496	1 (at bound)	0.999
1st_set	30%_1.0NRL-0.05TCNF-0.1GCA	7 days	4.40e4 ± 5.12e+03	428	1 (at bound)	0.999
1st_set	30%_1.0NRL-0.05TCNF-0.1GCA	14 days	5.84e4 ± 8.91e+03	507	1 (at bound)	0.999
1st_set	35%_1.0NRL-0.05TCNF-0.1GCA	1 hour	7.34e4 ± 8.3e+03	659	1 (at bound)	0.999
1st_set	35%_1.0NRL-0.05TCNF-0.1GCA	24 hours (1 day)	7.84e4 ± 6.7e+03	613	1 (at bound)	1
1st_set	35%_1.0NRL-0.05TCNF-0.1GCA	7 days	9.69e4 ± 1.01e4	638	1 (at bound)	1
1st_set	35%_1.0NRL-0.05TCNF-0.1GCA	11 days	9.87e4 ± 1.25e4	667	1 (at bound)	0.999
1st_set	40%_1.0NRL-0.05TCNF-0.1GCA	1 hour	7.32e4 ± 5.19e+03	764	1 (at bound)	1
1st_set	40%_1.0NRL-0.05TCNF-0.1GCA	24 hours (1 day)	7.67e4 ± 5.05e+03	728	1 (at bound)	1
1st_set	40%_1.0NRL-0.05TCNF-0.1GCA	4 days	8.17e4 ± 8.5e+03	768	1 (at bound)	1
1st_set	40%_1.0NRL-0.05TCNF-0.1GCA	7 days	8.99e4 ± 6.26e+03	744	1 (at bound)	1
1st_set	40%_1.0NRL-0.05TCNF-0.1GCA	9 days	1.01e5 ± 7.66e+03	802	1 (at bound)	1
1st_set	40%_1.0NRL-0.05TCNF-0.1GCA	14 days	1.01e5 ± 8.01e+03	751	1 (at bound)	1

Set	Formulation	Age	η0 ± CI95 (Pa·s)	λ (s)	n	R2(log10 η)
2nd_set	25%_1.0NRL-0.03TCNF-0.12GCA	1 hour	4.08e+03 ± 350	251	0.972	0.999
2nd_set	25%_1.0NRL-0.03TCNF-0.12GCA	24 hours (1 day)	5.34e+03 ± 612	256	0.983	0.999
2nd_set	25%_1.0NRL-0.03TCNF-0.12GCA	3 days	6.86e+03 ± 831	266	0.989	0.999
2nd_set	25%_1.0NRL-0.03TCNF-0.12GCA	6 days	9.16e+03 ± 1.36e+03	290	1 (at bound)	0.998
2nd_set	25%_1.0NRL-0.03TCNF-0.12GCA	13 days	9.64e+03 ± 1.1e+03	291	1 (at bound)	0.999
2nd_set	25%_1.0NRL-0.05TCNF-0.1GCA	1 hour	1.38e4 ± 1.6e+03	304	0.988	0.999
2nd_set	25%_1.0NRL-0.05TCNF-0.1GCA	24 hours (1 day)	1.81e4 ± 2.13e+03	303	0.997	0.999
2nd_set	25%_1.0NRL-0.05TCNF-0.1GCA	7 days	2.24e4 ± 3.3e+03	310	1 (at bound)	0.998
2nd_set	25%_1.0NRL-0.05TCNF-0.1GCA	11 days	2.55e4 ± 4.34e+03	336	1 (at bound)	0.998
2nd_set	25%_1.0NRL-0.07TCNF-0.08GCA	1 hour	3.39e4 ± 4.11e+03	360	1 (at bound)	0.999
2nd_set	25%_1.0NRL-0.07TCNF-0.08GCA	24 hours (1 day)	3.70e4 ± 3.85e+03	345	1 (at bound)	0.999
2nd_set	25%_1.0NRL-0.07TCNF-0.08GCA	3 days	4.06e4 ± 6.69e+03	351	1 (at bound)	0.998
2nd_set	25%_1.0NRL-0.07TCNF-0.08GCA	7 days	5.07e4 ± 9.47e+03	409	1 (at bound)	0.998
2nd_set	25%_1.0NRL-0.07TCNF-0.08GCA	14 days	5.41e4 ± 1.09e4	445	1 (at bound)	0.998
2nd_set	25%_1.0NRL-0.10TCNF-0.05GCA	1 hour	7.50e4 ± 1.12e4	504	1 (at bound)	0.999
2nd_set	25%_1.0NRL-0.10TCNF-0.05GCA	24 hours (1 day)	7.72e4 ± 9.37e+03	462	1 (at bound)	0.999

Set	Formulation	Age	η0 ± CI95 (Pa·s)	λ (s)	n	R2(log10 η)
2nd_set	25%_1.0NRL-0.10TCNF-0.05GCA	3 days	7.61e4 ± 8.7e+03	434	1 (at bound)	0.999
2nd_set	25%_1.0NRL-0.10TCNF-0.05GCA	6 days	8.10e4 ± 9.45e+03	456	1 (at bound)	0.999
2nd_set	25%_1.0NRL-0.10TCNF-0.05GCA	13 days	1.11e5 ± 1.77e4	558	1 (at bound)	0.999
2nd_set	25%_1.0NRL-0.12TCNF-0.03GCA	1 hour	8.20e4 ± 1.27e4	596	1 (at bound)	0.999
2nd_set	25%_1.0NRL-0.12TCNF-0.03GCA	24 hours (1 day)	8.87e4 ± 1.10e4	547	1 (at bound)	0.999
2nd_set	25%_1.0NRL-0.12TCNF-0.03GCA	3 days	1.07e5 ± 1.77e4	623	1 (at bound)	0.999
2nd_set	25%_1.0NRL-0.12TCNF-0.03GCA	7 days	1.20e5 ± 1.44e4	578	1 (at bound)	0.999
2nd_set	25%_1.0NRL-0.12TCNF-0.03GCA	14 days	1.39e5 ± 2.55e4	641	1 (at bound)	0.999
2nd_set	25%_1.0NRL-0.15TCNF-0.0GCA	1 hour	1.33e5 ± 2.17e4	723	1 (at bound)	0.999
2nd_set	25%_1.0NRL-0.15TCNF-0.0GCA	24 hours (1 day)	1.43e5 ± 2.61e4	714	1 (at bound)	0.999
2nd_set	25%_1.0NRL-0.15TCNF-0.0GCA	4 days	1.68e5 ± 2.85e4	734	1 (at bound)	0.999
2nd_set	25%_1.0NRL-0.15TCNF-0.0GCA	7 days	1.77e5 ± 2.65e4	722	1 (at bound)	0.999
2nd_set	25%_1.0NRL-0.15TCNF-0.0GCA	14 days	1.98e5 ± 3.82e4	831	1 (at bound)	0.999
3rd_set	25%_1.0NRL-0.07TCNF-0.08GCA	1 hour	3.39e4 ± 4.11e+03	360	1 (at bound)	0.999
3rd_set	25%_1.0NRL-0.07TCNF-0.08GCA	24 hours (1 day)	3.70e4 ± 3.85e+03	345	1 (at bound)	0.999
3rd_set	25%_1.0NRL-0.07TCNF-0.08GCA	3 days	4.06e4 ± 6.69e+03	351	1 (at bound)	0.998

Set	Formulation	Age	η0 ± CI95 (Pa·s)	λ (s)	n	R2(log10 η)
3rd_set	25%_1.0NRL-0.07TCNF-0.08GCA	7 days	5.07e4 ± 9.47e+03	409	1 (at bound)	0.998
3rd_set	25%_1.0NRL-0.07TCNF-0.08GCA	14 days	5.41e4 ± 1.09e4	445	1 (at bound)	0.998
3rd_set	25%_1.0NRL-0.07TCNF-0.0GCA	1 hour	3.48e4 ± 3.91e+03	397	0.993	0.999
3rd_set	25%_1.0NRL-0.07TCNF-0.0GCA	24 hours (1 day)	4.25e4 ± 4.47e+03	395	1 (at bound)	0.999
3rd_set	25%_1.0NRL-0.07TCNF-0.0GCA	3 days	4.65e4 ± 6.59e+03	418	1 (at bound)	0.999
3rd_set	25%_1.0NRL-0.07TCNF-0.0GCA	7 days	5.79e4 ± 9.4e+03	508	1 (at bound)	0.999
3rd_set	25%_1.0NRL-0.07TCNF-0.0GCA	29 days	6.76e4 ± 1.35e4	532	1 (at bound)	0.998
3rd_set	25%_1.0NRL-0.07TCNF-0.15GCA	1 hour	3.29e4 ± 5.08e+03	363	1 (at bound)	0.999
3rd_set	25%_1.0NRL-0.07TCNF-0.15GCA	24 hours (1 day)	3.62e4 ± 5.37e+03	341	1 (at bound)	0.999
3rd_set	25%_1.0NRL-0.07TCNF-0.15GCA	3 days	4.01e4 ± 7.69e+03	349	1 (at bound)	0.998
3rd_set	25%_1.0NRL-0.07TCNF-0.15GCA	6 days	4.37e4 ± 9.13e+03	371	1 (at bound)	0.997
3rd_set	25%_1.0NRL-0.07TCNF-0.15GCA	35 days	5.79e4 ± 1.42e4	404	1 (at bound)	0.997
3rd_set	25%_1.0NRL-0.07TCNF-0.1GCA	1 hour	3.11e4 ± 3.9e+03	366	1 (at bound)	0.999
3rd_set	25%_1.0NRL-0.07TCNF-0.1GCA	24 hours	3.87e4 ± 4.61e+03	365	1 (at bound)	0.999
3rd_set	25%_1.0NRL-0.07TCNF-0.1GCA	3 days	4.12e4 ± 5.76e+03	367	1 (at bound)	0.999
3rd_set	25%_1.0NRL-0.07TCNF-0.1GCA	7 days	4.42e4 ± 6.54e+03	353	1 (at bound)	0.999

Set	Formulation	Age	η0 ± CI95 (Pa·s)	λ (s)	n	R2(log10 η)
3rd_set	25%_1.0NRL-0.07TCNF-0.1GCA	29 days	4.86e4 ± 7.38e+03	416	1 (at bound)	0.999
3rd_set	25%_1.0NRL-0.07TCNF-0.25GCA	1 hour	2.66e4 ± 3.59e+03	331	1 (at bound)	0.999
3rd_set	25%_1.0NRL-0.07TCNF-0.25GCA	24 hours (1 day)	2.84e4 ± 4.74e+03	298	1 (at bound)	0.998
3rd_set	25%_1.0NRL-0.07TCNF-0.25GCA	3 days	3.48e4 ± 8.05e+03	348	1 (at bound)	0.997
3rd_set	25%_1.0NRL-0.07TCNF-0.25GCA	6 days	3.87e4 ± 1.01e4	355	1 (at bound)	0.996
3rd_set	25%_1.0NRL-0.07TCNF-0.25GCA	35 days	5.14e4 ± 1.72e4	386	1 (at bound)	0.994
3rd_set	25%_1.0NRL-0.07TCNF-0.2GCA	1 hour	3.28e4 ± 4.88e+03	373	1 (at bound)	0.999
3rd_set	25%_1.0NRL-0.07TCNF-0.2GCA	24 hours (1 day)	3.49e4 ± 5.78e+03	335	1 (at bound)	0.998
3rd_set	25%_1.0NRL-0.07TCNF-0.2GCA	3 days	3.81e4 ± 8.55e+03	335	1 (at bound)	0.997
3rd_set	25%_1.0NRL-0.07TCNF-0.2GCA	6 days	4.47e4 ± 1.04e4	353	1 (at bound)	0.997
3rd_set	25%_1.0NRL-0.07TCNF-0.2GCA	35 days	5.88e4 ± 1.81e4	416	1 (at bound)	0.995
4th_set	25%_1.0NRL-0.07TCNF-0.15GCA	1 hour	3.29e4 ± 5.08e+03	363	1 (at bound)	0.999
4th_set	25%_1.0NRL-0.07TCNF-0.15GCA	24 hours (1 day)	3.62e4 ± 5.37e+03	341	1 (at bound)	0.999
4th_set	25%_1.0NRL-0.07TCNF-0.15GCA	3 days	4.01e4 ± 7.69e+03	349	1 (at bound)	0.998
4th_set	25%_1.0NRL-0.07TCNF-0.15GCA	6 days	4.37e4 ± 9.13e+03	371	1 (at bound)	0.997
4th_set	25%_1.0NRL-0.07TCNF-0.15GCA	35 days	5.79e4 ± 1.42e4	404	1 (at bound)	0.997

Set	Formulation	Age	η0 ± CI95 (Pa·s)	λ (s)	n	R2(log10 η)
4th_set	25%_1.0NRL-0.07TCNF-0.15GNP	1 hour	1.02e5 ± 3.97e4	607	1 (at bound)	0.993
4th_set	25%_1.0NRL-0.07TCNF-0.15GNP	24 hours (1 day)	1.32e5 ± 5.09e4	663	1 (at bound)	0.994
4th_set	25%_1.0NRL-0.07TCNF-0.15GNP	3 days	1.50e5 ± 4.30e4	570	1 (at bound)	0.996
4th_set	25%_1.0NRL-0.07TCNF-0.15GNP	6 days	1.53e5 ± 4.14e4	546	1 (at bound)	0.997
4th_set	25%_1.0NRL-0.07TCNF-0.15GNP	10 days	1.63e5 ± 4.19e4	542	1 (at bound)	0.997
4th_set	25%_1.0NRL-0.07TCNF-0.15GNP	15 days	1.70e5 ± 4.96e4	391	1 (at bound)	0.995
4th_set	25%_1.0NRL-0.07TCNF-0.15MWCNT	1 hour	1.05e5 ± 1.52e4	622	1 (at bound)	0.999
4th_set	25%_1.0NRL-0.07TCNF-0.15MWCNT	24 hours (1 day)	1.06e5 ± 1.30e4	517	1 (at bound)	0.999
4th_set	25%_1.0NRL-0.07TCNF-0.15MWCNT	3 days	1.37e5 ± 1.79e4	582	1 (at bound)	0.999
4th_set	25%_1.0NRL-0.07TCNF-0.15MWCNT	6 days	1.49e5 ± 1.95e4	570	1 (at bound)	0.999
4th_set	25%_1.0NRL-0.07TCNF-0.15MWCNT	10 days	1.60e5 ± 2.68e4	489	1 (at bound)	0.999
4th_set	25%_1.0NRL-0.07TCNF-0.15MWCNT	15 days	3.38e5 ± 1.29e5	1.07e+03	1 (at bound)	0.996
4th_set	25%_1.0NRL-0.07TCNF-0.15MWCNT-COOH	1 hour	1.29e5 ± 3.29e4	683	1 (at bound)	0.997
4th_set	25%_1.0NRL-0.07TCNF-0.15MWCNT-COOH	24 hours (1 day)	1.21e5 ± 2.12e4	562	1 (at bound)	0.999
4th_set	25%_1.0NRL-0.07TCNF-0.15MWCNT-COOH	4 days	1.44e5 ± 2.45e4	570	1 (at bound)	0.999

Set	Formulation	Age	η0 ± CI95 (Pa·s)	λ (s)	n	R2(log10 η)
4th_set	25%_1.0NRL-0.07TCNF-0.15MWCNT-COOH	8 days	1.58e5 ± 2.67e4	561	1 (at bound)	0.999
4th_set	25%_1.0NRL-0.07TCNF-0.15MWCNT-COOH	13 days	1.62e5 ± 2.98e4	586	1 (at bound)	0.998
4th_set	25%_1.0NRL-0.07TCNF-0.15graphite	1 hour	2.87e4 ± 4.05e+03	329	1 (at bound)	0.999
4th_set	25%_1.0NRL-0.07TCNF-0.15graphite	24 hours (1 day)	3.30e4 ± 4.69e+03	335	1 (at bound)	0.999
4th_set	25%_1.0NRL-0.07TCNF-0.15graphite	4 days	4.01e4 ± 8.17e+03	376	1 (at bound)	0.997
4th_set	25%_1.0NRL-0.07TCNF-0.15graphite	8 days	4.74e4 ± 8.86e+03	396	1 (at bound)	0.998
4th_set	25%_1.0NRL-0.07TCNF-0.15graphite	14 days	4.67e4 ± 1.09e4	414	1 (at bound)	0.997

Table S2. GMM fits: per-modulus goodness of fit and grid sensitivity.

Fixed relaxation-time grid τi = 0.001-100 s (six modes, decade spacing). Ge_relchange columns give the relative change of Ge when the grid is shifted by a factor of 3 or 1/3.

Set	Formulation	Age	Ge (Pa)	R2(G')	R2(G")	rel. RMSE G'	rel. RMSE G"	ΔGe (grid ×3)	ΔGe (grid /3)
1st_set	20%_1.0NRL-0.05TCNF-0.1GCA	1 hour	16.7	-0.169	0.983	0.0751	0.0328	-1	0.257
1st_set	20%_1.0NRL-0.05TCNF-0.1GCA	24 hours (1 day)	34.7	0.986	0.95	0.0144	0.0321	0.113	0.0965
1st_set	20%_1.0NRL-0.05TC	4 days	39.7	0.988	0.918	0.0142	0.0331	0.124	0.108

Set	Formulation	Age	Ge (Pa)	R2(G')	R2(G")	rel. RMSE G'	rel. RMSE G"	ΔGe (grid ×3)	ΔGe (grid /3)
	NF-0.1GCA								
1st_set	20%_1.0 NRL-0.05TC NF-0.1GCA	11 days	58.9	0.962	0.852	0.0215	0.0348	0.0696	0.0535
1st_set	25%_1.0 NRL-0.05TC NF-0.1GCA	1 hour	46.6	-1.8	0.933	0.0821	0.035	-1	0.303
1st_set	25%_1.0 NRL-0.05TC NF-0.1GCA	24 hours (1 day)	93	0.918	0.838	0.0276	0.0338	-0.399	0.135
1st_set	25%_1.0 NRL-0.05TC NF-0.1GCA	7 days	144	0.93	0.643	0.0284	0.0666	-5.88e−3	-0.0189
1st_set	25%_1.0 NRL-0.05TC NF-0.1GCA	11 days	144	0.916	0.801	0.0279	0.034	-0.308	0.0955
1st_set	30%_1.0 NRL-0.05TC NF-0.1GCA	1 hour	115	-2.87	0.867	0.0875	0.0363	-1	0.265
1st_set	30%_1.0 NRL-0.05TC NF-0.1GCA	24 hours (1 day)	194	0.853	0.753	0.0357	0.0348	-0.779	0.127
1st_set	30%_1.0 NRL-0.05TC NF-0.1GCA	4 days	248	0.779	0.637	0.0406	0.0347	-0.764	0.0897
1st_set	30%_1.0 NRL-0.05TC NF-0.1GCA	7 days	259	0.899	0.657	0.0315	0.0349	-0.359	0.0787
1st_set	30%_1.0 NRL-	14 days	316	0.81	0.668	0.0377	0.0336	-0.0545	0.0567

Set	Formulation	Age	Ge (Pa)	R2(G')	R2(G")	rel. RMSE G'	rel. RMSE G"	ΔGe (grid ×3)	ΔGe (grid /3)
	0.05TC NF-0.1GCA								
1st_set	35%_1.0 NRL-0.05TC NF-0.1GCA	1 hour	275	-0.714	0.755	0.0743	0.036	-1	0.207
1st_set	35%_1.0 NRL-0.05TC NF-0.1GCA	24 hours (1 day)	379	0.842	0.736	0.038	0.0345	-0.849	0.12
1st_set	35%_1.0 NRL-0.05TC NF-0.1GCA	7 days	513	0.881	0.443	0.0348	0.0339	-0.445	0.0682
1st_set	35%_1.0 NRL-0.05TC NF-0.1GCA	11 days	528	0.892	0.613	0.0327	0.0326	0.0798	0.0642
1st_set	40%_1.0 NRL-0.05TC NF-0.1GCA	1 hour	300	0.713	0.898	0.0529	0.0349	-1	0.241
1st_set	40%_1.0 NRL-0.05TC NF-0.1GCA	24 hours (1 day)	405	0.86	0.908	0.0422	0.0351	-1	0.123
1st_set	40%_1.0 NRL-0.05TC NF-0.1GCA	4 days	409	0.892	0.922	0.0379	0.0351	-1	0.136
1st_set	40%_1.0 NRL-0.05TC NF-0.1GCA	7 days	475	0.926	0.92	0.0327	0.0336	-0.833	0.116
1st_set	40%_1.0 NRL-0.05TC NF-0.1GCA	9 days	508	0.929	0.919	0.0318	0.0333	-0.913	0.134

Set	Formulation	Age	Ge (Pa)	R2(G')	R2(G")	rel. RMSE G'	rel. RMSE G"	ΔGe (grid ×3)	ΔGe (grid /3)
1st_set	40%_1.0NRL-0.05TCNF-0.1GCA	14 days	544	0.941	0.918	0.0291	0.0339	-0.615	0.122
2nd_set	25%_1.0NRL-0.03TCNF-0.12GCA	1 hour	15	0.673	0.983	0.0547	0.0332	-1	0.299
2nd_set	25%_1.0NRL-0.03TCNF-0.12GCA	24 hours (1 day)	28.6	0.94	0.956	0.0301	0.031	-0.409	0.106
2nd_set	25%_1.0NRL-0.03TCNF-0.12GCA	3 days	35.7	0.931	0.923	0.032	0.0322	-0.597	0.116
2nd_set	25%_1.0NRL-0.03TCNF-0.12GCA	6 days	48.7	0.864	0.864	0.0381	0.0329	-0.774	0.0888
2nd_set	25%_1.0NRL-0.03TCNF-0.12GCA	13 days	46.9	0.903	0.858	0.0345	0.0342	-1	0.212
2nd_set	25%_1.0NRL-0.05TCNF-0.1GCA	1 hour	46.6	-1.8	0.933	0.0821	0.035	-1	0.303
2nd_set	25%_1.0NRL-0.05TCNF-0.1GCA	24 hours (1 day)	93	0.918	0.838	0.0276	0.0338	-0.399	0.135
2nd_set	25%_1.0NRL-0.05TCNF-0.1GCA	7 days	144	0.93	0.643	0.0284	0.0666	-5.88e−3	-0.0189

Set	Formulation	Age	Ge (Pa)	R2(G')	R2(G")	rel. RMSE G'	rel. RMSE G"	ΔGe (grid ×3)	ΔGe (grid /3)
2nd_set	25%_1.0 NRL-0.05TCNF-0.1GCA	11 days	144	0.916	0.801	0.0279	0.034	-0.308	0.0955
2nd_set	25%_1.0 NRL-0.07TCNF-0.08GCA	1 hour	139	-0.514	0.867	0.068	0.0324	-1	0.203
2nd_set	25%_1.0 NRL-0.07TCNF-0.08GCA	24 hours (1 day)	197	0.821	0.784	0.0366	0.0329	-0.567	0.12
2nd_set	25%_1.0 NRL-0.07TCNF-0.08GCA	3 days	232	0.892	0.718	0.0315	0.0336	-0.342	0.1
2nd_set	25%_1.0 NRL-0.07TCNF-0.08GCA	7 days	274	0.816	0.685	0.0369	0.0345	-0.477	0.0832
2nd_set	25%_1.0 NRL-0.07TCNF-0.08GCA	14 days	298	0.829	0.647	0.0361	0.0341	-0.409	0.0669
2nd_set	25%_1.0 NRL-0.10TCNF-0.05GCA	1 hour	285	-1.17	0.718	0.0753	0.0359	-1	0.193
2nd_set	25%_1.0 NRL-0.10TCNF-0.05GCA	24 hours (1 day)	413	0.805	0.32	0.0389	0.0342	-0.266	0.0764
2nd_set	25%_1.0 NRL-0.10TC	3 days	437	0.914	0.173	0.0294	0.035	-0.283	0.0671

Set	Formulation	Age	Ge (Pa)	R2(G')	R2(G")	rel. RMSE G'	rel. RMSE G"	ΔGe (grid ×3)	ΔGe (grid /3)
	NF-0.05GCA								
2nd_set	25%_1.0 NRL-0.10TC NF-0.05GCA	6 days	455	0.869	0.15	0.0349	0.0334	-0.448	0.0751
2nd_set	25%_1.0 NRL-0.10TC NF-0.05GCA	13 days	609	0.855	-0.0336	0.0349	0.034	-0.251	0.0643
2nd_set	25%_1.0 NRL-0.12TC NF-0.03GCA	1 hour	292	0.441	0.711	0.0662	0.036	-1	0.194
2nd_set	25%_1.0 NRL-0.12TC NF-0.03GCA	24 hours (1 day)	427	0.895	-0.541	0.0392	0.0331	-1	0.0972
2nd_set	25%_1.0 NRL-0.12TC NF-0.03GCA	3 days	495	0.937	-1.11	0.0317	0.0369	-1	0.129
2nd_set	25%_1.0 NRL-0.12TC NF-0.03GCA	7 days	429	0.797	0.157	0.0474	0.0355	-1	0.384
2nd_set	25%_1.0 NRL-0.12TC NF-0.03GCA	14 days	784	0.933	0.617	0.0318	0.037	-0.536	0.0224
2nd_set	25%_1.0 NRL-0.15TC NF-0.0GCA	1 hour	529	0.808	0.0474	0.0528	0.0388	-1	0.156

Set	Formulation	Age	Ge (Pa)	R2(G')	R2(G")	rel. RMSE G'	rel. RMSE G"	ΔGe (grid ×3)	ΔGe (grid /3)
2nd_set	25%_1.0NRL-0.15TCNF-0.0GCA	24 hours (1 day)	747	0.937	0.71	0.0363	0.0355	-1	0.0597
2nd_set	25%_1.0NRL-0.15TCNF-0.0GCA	4 days	823	0.94	0.783	0.0353	0.0373	-1	0.0864
2nd_set	25%_1.0NRL-0.15TCNF-0.0GCA	7 days	910	0.927	0.949	0.037	0.0363	-1	0.0585
2nd_set	25%_1.0NRL-0.15TCNF-0.0GCA	14 days	1.07e+03	0.93	0.954	0.0359	0.036	-0.688	0.0137
3rd_set	25%_1.0NRL-0.07TCNF-0.08GCA	1 hour	139	-0.514	0.867	0.068	0.0324	-1	0.203
3rd_set	25%_1.0NRL-0.07TCNF-0.08GCA	24 hours (1 day)	197	0.821	0.784	0.0366	0.0329	-0.567	0.12
3rd_set	25%_1.0NRL-0.07TCNF-0.08GCA	3 days	232	0.892	0.718	0.0315	0.0336	-0.342	0.1
3rd_set	25%_1.0NRL-0.07TCNF-0.08GCA	7 days	274	0.816	0.685	0.0369	0.0345	-0.477	0.0832
3rd_set	25%_1.0NRL-0.07TCNF-0.08GCA	14 days	298	0.829	0.647	0.0361	0.0341	-0.409	0.0669

Set	Formulation	Age	Ge (Pa)	R2(G')	R2(G")	rel. RMSE G'	rel. RMSE G"	ΔGe (grid ×3)	ΔGe (grid /3)
3rd_set	25%_1.0NRL-0.07TCNF-0.0GCA	1 hour	128	-2.1	0.876	0.0809	0.0352	-1	0.247
3rd_set	25%_1.0NRL-0.07TCNF-0.0GCA	24 hours (1 day)	232	0.848	0.807	0.0339	0.0333	-0.48	0.096
3rd_set	25%_1.0NRL-0.07TCNF-0.0GCA	3 days	272	0.83	0.774	0.0359	0.0353	-0.302	0.0565
3rd_set	25%_1.0NRL-0.07TCNF-0.0GCA	7 days	306	0.852	0.715	0.0339	0.0363	-0.49	0.066
3rd_set	25%_1.0NRL-0.07TCNF-0.0GCA	29 days	357	0.804	0.693	0.0358	0.0355	-0.41	0.0644
3rd_set	25%_1.0NRL-0.07TCNF-0.15GCA	1 hour	128	-0.674	0.891	0.0652	0.0339	-1	0.206
3rd_set	25%_1.0NRL-0.07TCNF-0.15GCA	24 hours (1 day)	195	0.866	0.769	0.0332	0.0326	-0.287	0.0835
3rd_set	25%_1.0NRL-0.07TCNF-0.15GCA	3 days	223	0.922	0.786	0.0269	0.033	-0.106	0.0909
3rd_set	25%_1.0NRL-0.07TCNF-0.15GCA	6 days	240	0.887	0.715	0.0308	0.0344	-0.324	0.0817

Set	Formulation	Age	Ge (Pa)	R2(G')	R2(G")	rel. RMSE G'	rel. RMSE G"	ΔGe (grid ×3)	ΔGe (grid /3)
3rd_set	25%_1.0NRL-0.07TCNF-0.15GCA	35 days	338	0.81	0.605	0.0355	0.0361	-0.247	0.046
3rd_set	25%_1.0NRL-0.07TCNF-0.1GCA	1 hours	144	0.679	0.821	0.0452	0.0332	-0.983	0.134
3rd_set	25%_1.0NRL-0.07TCNF-0.1GCA	24 hours (1¸day)	177	0.91	0.784	0.029	0.0339	-0.444	0.125
3rd_set	25%_1.0NRL-0.07TCNF-0.1GCA	3 days	226	0.881	0.683	0.0331	0.0339	-0.72	0.122
3rd_set	25%_1.0NRL-0.07TCNF-0.1GCA	7 days	243	0.924	0.698	0.0286	0.0324	-0.209	0.0923
3rd_set	25%_1.0NRL-0.07TCNF-0.1GCA	29 days	284	0.867	0.545	0.0338	0.0347	-0.285	0.0611
3rd_set	25%_1.0NRL-0.07TCNF-0.25GCA	1 hour	110	0.124	0.881	0.061	0.0341	-1	0.195
3rd_set	25%_1.0NRL-0.07TCNF-0.25GCA	24 hours (1 day)	162	0.925	0.72	0.0285	0.0343	-0.237	0.0822
3rd_set	25%_1.0NRL-0.07TCNF-0.25GCA	3 days	188	0.888	0.659	0.032	0.0353	-0.452	0.0876

Set	Formulation	Age	Ge (Pa)	R2(G')	R2(G")	rel. RMSE G'	rel. RMSE G"	ΔGe (grid ×3)	ΔGe (grid /3)
3rd_set	25%_1.0NRL-0.07TCNF-0.25GCA	6 days	215	0.887	0.575	0.0326	0.0358	-0.389	0.0689
3rd_set	25%_1.0NRL-0.07TCNF-0.25GCA	35 days	285	0.859	0.574	0.0331	0.0344	-0.313	0.0639
3rd_set	25%_1.0NRL-0.07TCNF-0.2GCA	1 hour	115	-1.61	0.889	0.0758	0.0343	-1	0.222
3rd_set	25%_1.0NRL-0.07TCNF-0.2GCA	24 hours (1 day)	182	0.884	0.744	0.0306	0.0348	-0.52	0.106
3rd_set	25%_1.0NRL-0.07TCNF-0.2GCA	3 days	216	0.91	0.746	0.0282	0.0344	-0.318	0.0884
3rd_set	25%_1.0NRL-0.07TCNF-0.2GCA	6 days	263	0.929	0.756	0.0256	0.0341	0.0367	0.0623
3rd_set	25%_1.0NRL-0.07TCNF-0.2GCA	35 days	328	0.818	0.618	0.035	0.0357	-0.088	0.0501
4th_set	25%_1.0NRL-0.07TCNF-0.15GCA	1 hour	128	-0.674	0.891	0.0652	0.0339	-1	0.206
4th_set	25%_1.0NRL-0.07TCNF-0.15GCA	24 hours (1 day)	195	0.866	0.769	0.0332	0.0326	-0.287	0.0835

Set	Formulation	Age	Ge (Pa)	R2(G')	R2(G")	rel. RMSE G'	rel. RMSE G"	ΔGe (grid ×3)	ΔGe (grid /3)
4th_set	25%_1.0NRL-0.07TCNF-0.15GCA	3 days	223	0.922	0.786	0.0269	0.033	-0.106	0.0909
4th_set	25%_1.0NRL-0.07TCNF-0.15GCA	6 days	240	0.887	0.715	0.0308	0.0344	-0.324	0.0817
4th_set	25%_1.0NRL-0.07TCNF-0.15GCA	35 days	338	0.81	0.605	0.0355	0.0361	-0.247	0.046
4th_set	25%_1.0NRL-0.07TCNF-0.15GNP	1 hour	379	-1.08	0.848	0.0657	0.0339	-1	0.158
4th_set	25%_1.0NRL-0.07TCNF-0.15GNP	24 hours (1 day)	540	-0.399	0.702	0.0565	0.0383	-1	0.106
4th_set	25%_1.0NRL-0.07TCNF-0.15GNP	3 days	718	-0.0351	0.425	0.0566	0.045	-0.745	0.0564
4th_set	25%_1.0NRL-0.07TCNF-0.15GNP	6 days	742	-0.129	0.928	0.055	0.0392	-1	0.0614
4th_set	25%_1.0NRL-0.07TCNF-0.15GNP	10 days	852	0.504	0.132	0.0435	0.0348	-0.26	0.0306
4th_set	25%_1.0NRL-	15 days	1.04e+03	0.346	0.61	0.0458	0.0509	-0.0712	-0.0236

Set	Formulation	Age	Ge (Pa)	R2(G')	R2(G")	rel. RMSE G'	rel. RMSE G"	ΔGe (grid ×3)	ΔGe (grid /3)
	0.07TCNF-0.15GNP								
4th_set	25%_1.0NRL-0.07TCNF-0.15MWCNT	1 hour	363	0.35	0.684	0.0682	0.0358	-1	0.263
4th_set	25%_1.0NRL-0.07TCNF-0.15MWCNT	24 hours (1 day)	505	0.76	0.961	0.0591	0.038	-1	0.0288
4th_set	25%_1.0NRL-0.07TCNF-0.15MWCNT	3 days	644	0.755	0.972	0.051	0.0318	-1	0.113
4th_set	25%_1.0NRL-0.07TCNF-0.15MWCNT	6 days	759	0.836	0.838	0.0445	0.0371	-1	0.0476
4th_set	25%_1.0NRL-0.07TCNF-0.15MWCNT	10 days	950	0.828	0.793	0.0445	0.0357	-0.683	0.0293
4th_set	25%_1.0NRL-0.07TCNF-0.15MWCNT	15 days	1.5e+03	0.814	0.92	0.04	0.0349	-1	0.0774
4th_set	25%_1.0NRL-0.07TCNF-0.15MWCNT-COOH	1 hour	436	-1.32	0.828	0.0802	0.035	-1	0.196
4th_set	25%_1.0NRL-0.07TC	24 hours (1 day)	614	0.762	0.568	0.0474	0.0357	-0.881	0.0429

Set	Formulation	Age	Ge (Pa)	R2(G')	R2(G")	rel. RMSE G'	rel. RMSE G"	ΔGe (grid ×3)	ΔGe (grid /3)
	NF-0.15MWCNT-COOH								
4th_set	25%_1.0NRL-0.07TCNF-0.15MWCNT-COOH	4 days	732	0.803	0.883	0.0485	0.0377	-1	0.0513
4th_set	25%_1.0NRL-0.07TCNF-0.15MWCNT-COOH	8 days	768	0.823	0.971	0.0428	0.0336	-1	0.0903
4th_set	25%_1.0NRL-0.07TCNF-0.15MWCNT-COOH	13 days	915	0.865	0.821	0.0375	0.035	-0.478	0.0318
4th_set	25%_1.0NRL-0.07TCNF-0.15graphite	1 hour	107	-0.711	0.887	0.0739	0.0335	-1	0.25
4th_set	25%_1.0NRL-0.07TCNF-0.15graphite	24 hours (1 day)	168	0.863	0.782	0.0346	0.0345	-0.739	0.132
4th_set	25%_1.0NRL-0.07TCNF-0.15graphite	4 days	212	0.901	0.707	0.031	0.0344	-0.436	0.105
4th_set	25%_1.0NRL-0.07TCNF-0.15graphite	8 days	254	0.804	0.704	0.0385	0.0335	-0.291	0.0824

Set	Formulation	Age	Ge (Pa)	R2(G')	R2(G")	rel. RMSE G'	rel. RMSE G"	ΔGe (grid ×3)	ΔGe (grid /3)
4th_set	25%_1.0NRL-0.07TCNF-0.15graphite	13 days	253	0.897	0.718	0.0292	0.034	-0.336	0.106

Table S3. Cox–Merz deviation metric at the late-storage states.

Δ(ω) = log10[|η*|(ω)/η(γ̇=ω)] interpolated on a common logarithmic grid over the overlapping range.

Set	Formulation	Age	Mean	Δ
1st_set	20%_1.0NRL-0.05TCNF-0.1GCA	11 days	0.368	2.33
1st_set	25%_1.0NRL-0.05TCNF-0.1GCA	11 days	0.545	3.51
1st_set	30%_1.0NRL-0.05TCNF-0.1GCA	14 days	0.683	4.82
1st_set	35%_1.0NRL-0.05TCNF-0.1GCA	11 days	0.792	6.2
1st_set	40%_1.0NRL-0.05TCNF-0.1GCA	14 days	0.871	7.44
2nd_set	25%_1.0NRL-0.03TCNF-0.12GCA	13 days	0.482	3.03
2nd_set	25%_1.0NRL-0.05TCNF-0.1GCA	11 days	0.545	3.51
2nd_set	25%_1.0NRL-0.07TCNF-0.08GCA	14 days	0.644	4.41
2nd_set	25%_1.0NRL-0.10TCNF-0.05GCA	13 days	0.689	4.89
2nd_set	25%_1.0NRL-0.12TCNF-0.03GCA	14 days	0.764	5.81
2nd_set	25%_1.0NRL-0.15TCNF-0.0GCA	14 days	0.86	7.24
3rd_set	25%_1.0NRL-0.07TCNF-0.08GCA	14 days	0.644	4.41
3rd_set	25%_1.0NRL-0.07TCNF-0.0GCA	29 days	0.701	5.03
3rd_set	25%_1.0NRL-0.07TCNF-0.15GCA	35 days	0.603	4.01
3rd_set	25%_1.0NRL-0.07TCNF-0.1GCA	29 days	0.619	4.16
3rd_set	25%_1.0NRL-0.07TCNF-0.25GCA	35 days	0.586	3.86
3rd_set	25%_1.0NRL-0.07TCNF-0.2GCA	35 days	0.601	3.99
4th_set	25%_1.0NRL-0.07TCNF-0.15GCA	35 days	0.603	4.01
4th_set	25%_1.0NRL-0.07TCNF-0.15GNP	15 days	0.567	3.69
4th_set	25%_1.0NRL-0.07TCNF-0.15MWCNT	15 days	0.861	7.26
4th_set	25%_1.0NRL-0.07TCNF-0.15MWCNT-COOH	13 days	0.71	5.13
4th_set	25%_1.0NRL-0.07TCNF-0.15graphite	14 days	0.623	4.19

Table S4. Late-storage comparison states and residual aging drift.

Set	Formulation	Late-storage age	G'(1 Hz) (Pa)	ΔG'/G' (%/day, final interval)
1st_set	20%_1.0NRL-0.05TCNF-0.1GCA	11 days	104	5.5
1st_set	25%_1.0NRL-0.05TCNF-0.1GCA	11 days	261	3.12
1st_set	30%_1.0NRL-0.05TCNF-0.1GCA	14 days	539	2.27
1st_set	35%_1.0NRL-0.05TCNF-0.1GCA	11 days	922	0.358
1st_set	40%_1.0NRL-0.05TCNF-0.1GCA	14 days	1.05e+03	1.21
2nd_set	25%_1.0NRL-0.03TCNF-0.12GCA	13 days	103	1.69
2nd_set	25%_1.0NRL-0.05TCNF-0.1GCA	11 days	261	3.12
2nd_set	25%_1.0NRL-0.07TCNF-0.08GCA	14 days	513	0.95
2nd_set	25%_1.0NRL-0.10TCNF-0.05GCA	13 days	1.04e+03	4.33
2nd_set	25%_1.0NRL-0.12TCNF-0.03GCA	14 days	1.33e+03	3.15
2nd_set	25%_1.0NRL-0.15TCNF-0.0GCA	14 days	1.88e+03	1.33
3rd_set	25%_1.0NRL-0.07TCNF-0.08GCA	14 days	513	0.95
3rd_set	25%_1.0NRL-0.07TCNF-0.0GCA	29 days	598	0.603
3rd_set	25%_1.0NRL-0.07TCNF-0.15GCA	35 days	552	1.08
3rd_set	25%_1.0NRL-0.07TCNF-0.1GCA	29 days	487	0.44
3rd_set	25%_1.0NRL-0.07TCNF-0.2GCA	35 days	538	0.665
3rd_set	25%_1.0NRL-0.07TCNF-0.25GCA	35 days	482	0.96
4th_set	25%_1.0NRL-0.07TCNF-0.15GCA	35 days	552	1.08

Set	Formulation	Late-storage age	G'(1 Hz) (Pa)	ΔG'/G' (%/day, final interval)
4th_set	25%_1.0NRL-0.07TCNF-0.15GNP	15 days	1.49e+03	3.28
4th_set	25%_1.0NRL-0.07TCNF-0.15graphite	13 days	446	0.181
4th_set	25%_1.0NRL-0.07TCNF-0.15MWCNT-COOH	13 days	1.49e+03	1.44
4th_set	25%_1.0NRL-0.07TCNF-0.15MWCNT	15 days	2.51e+03	10.8

Figure S1. GMM residuals at the late-storage states.

Relative residuals of the standard-grid GMM fits (Section 4.4) for each formulation at its late-storage age.

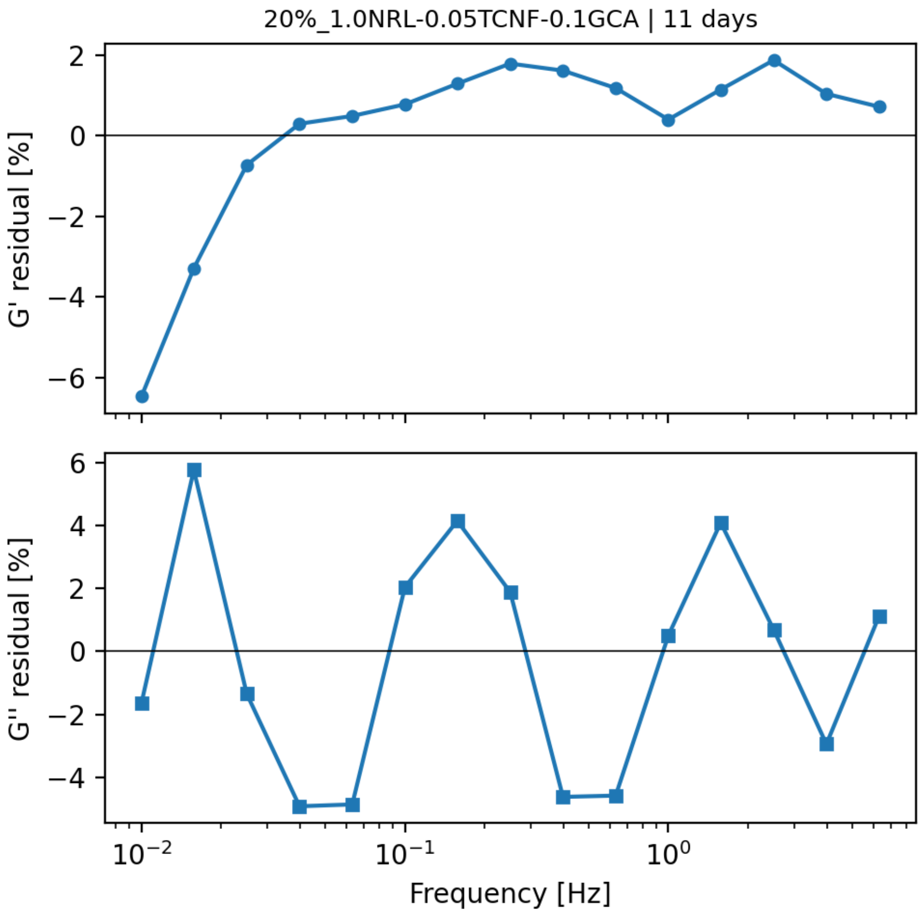

20%_1.0NRL-0.05TCNF-0.1GCA | 11 days
G' residual [%]
G'' residual [%]
Frequency [Hz]

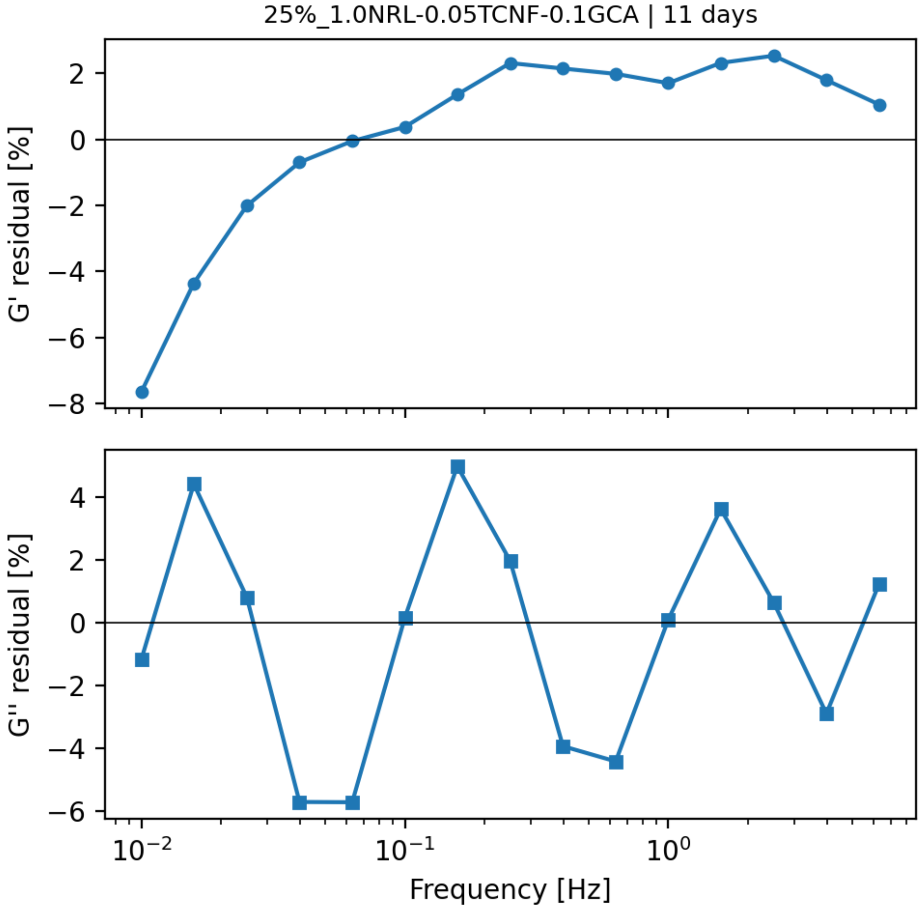

25%_1.0NRL-0.05TCNF-0.1GCA | 11 days
G' residual [%]
G'' residual [%]
Frequency [Hz]

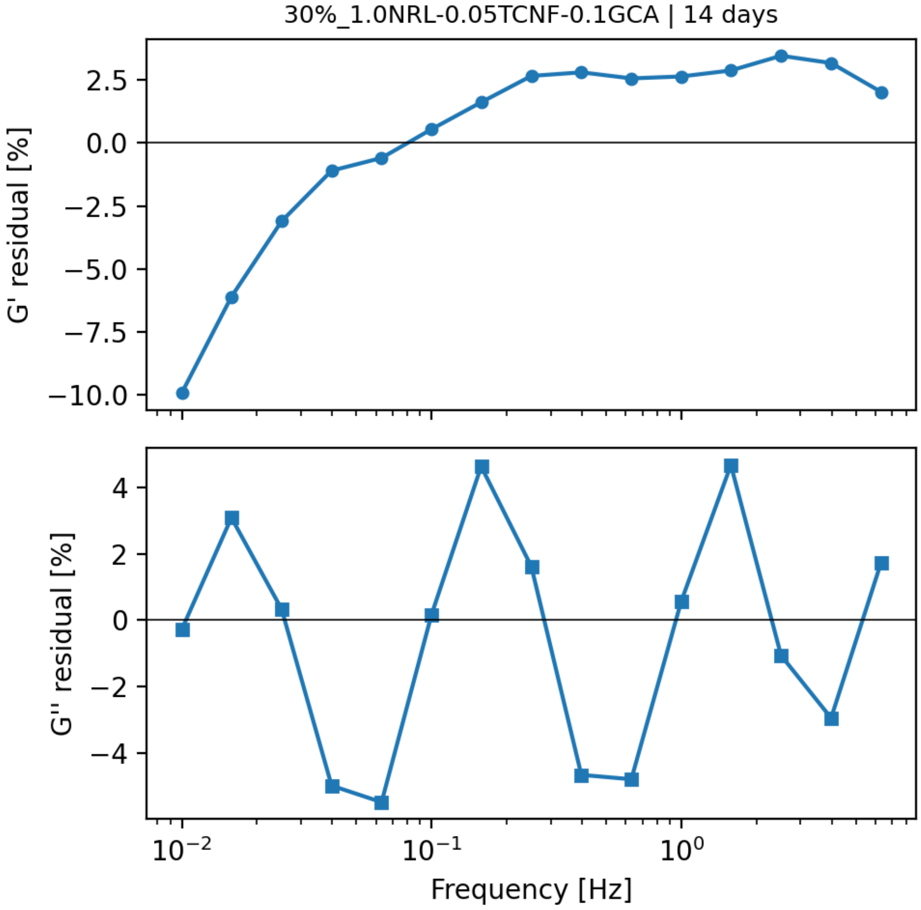

30%_1.0NRL-0.05TCNF-0.1GCA | 14 days
G' residual [%]
2.5
0.0
−2.5
−5.0
−7.5
−10.0
G'' residual [%]
4
2
0
−2
−4
10^{-2}
10^{-1}
10^{0}
Frequency [Hz]

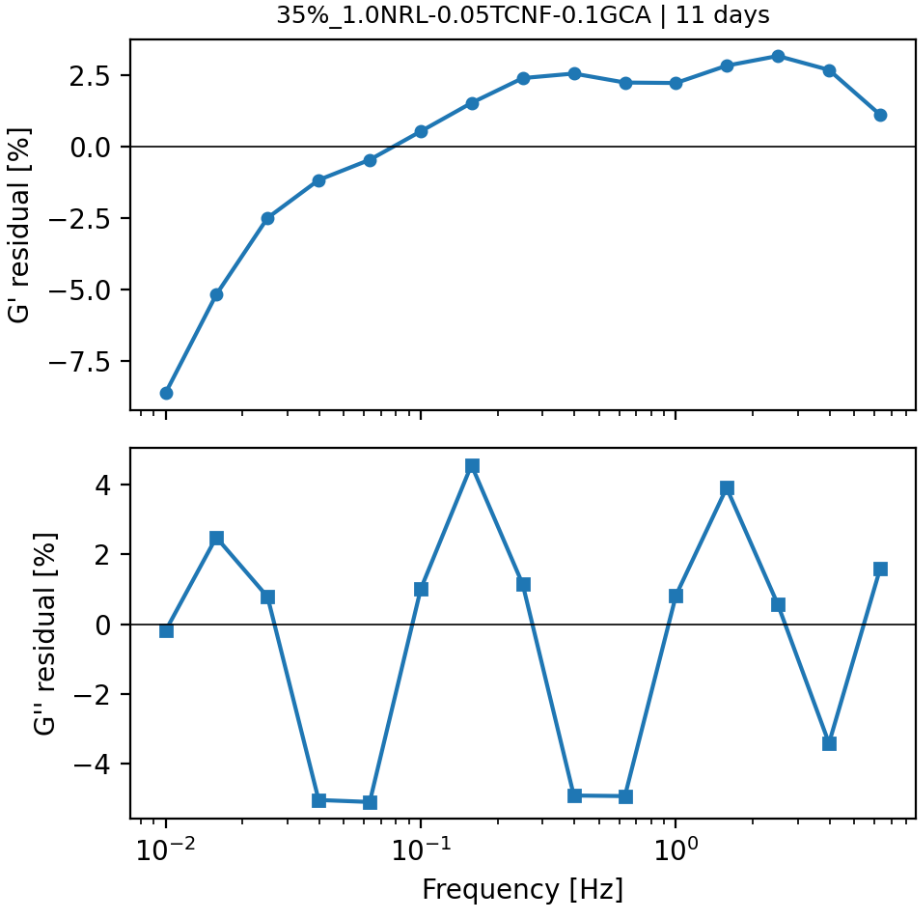
35%_1.0NRL-0.05TCNF-0.1GCA | 11 days
G' residual [%]
2.5
0.0
−2.5
−5.0
−7.5
G'' residual [%]
4
2
0
−2
−4
10^{-2}
10^{-1}
10^{0}
Frequency [Hz]

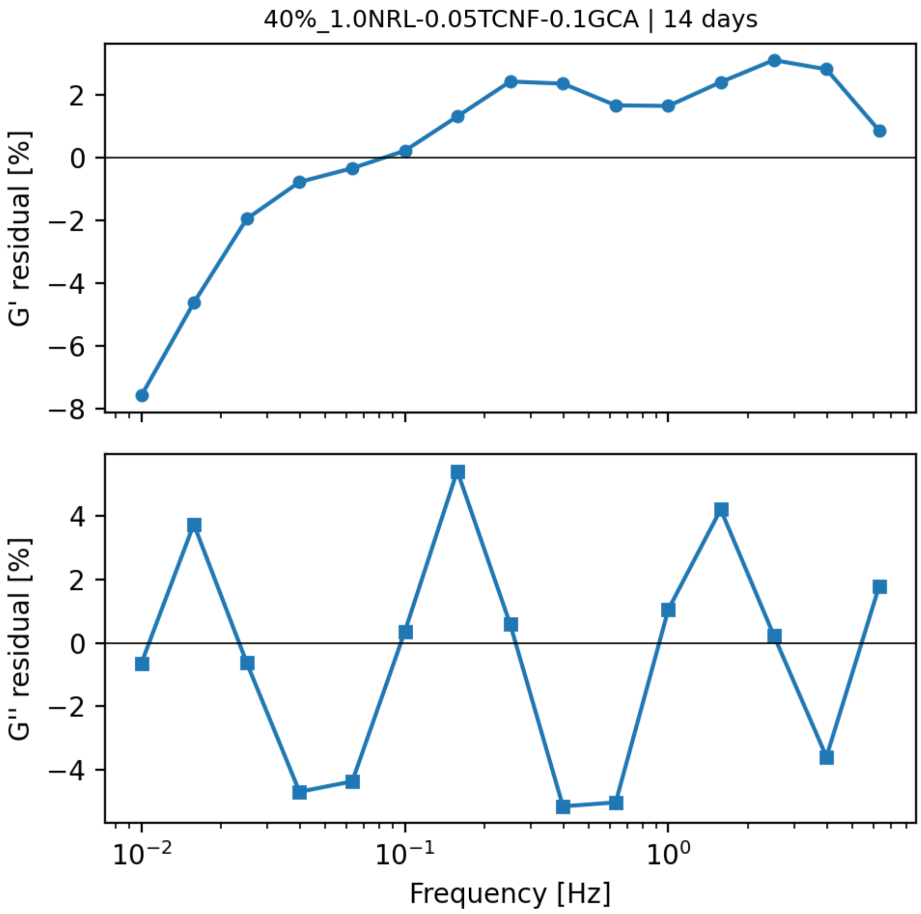

40%_1.0NRL-0.05TCNF-0.1GCA | 14 days
G' residual [%]
G'' residual [%]
Frequency [Hz]

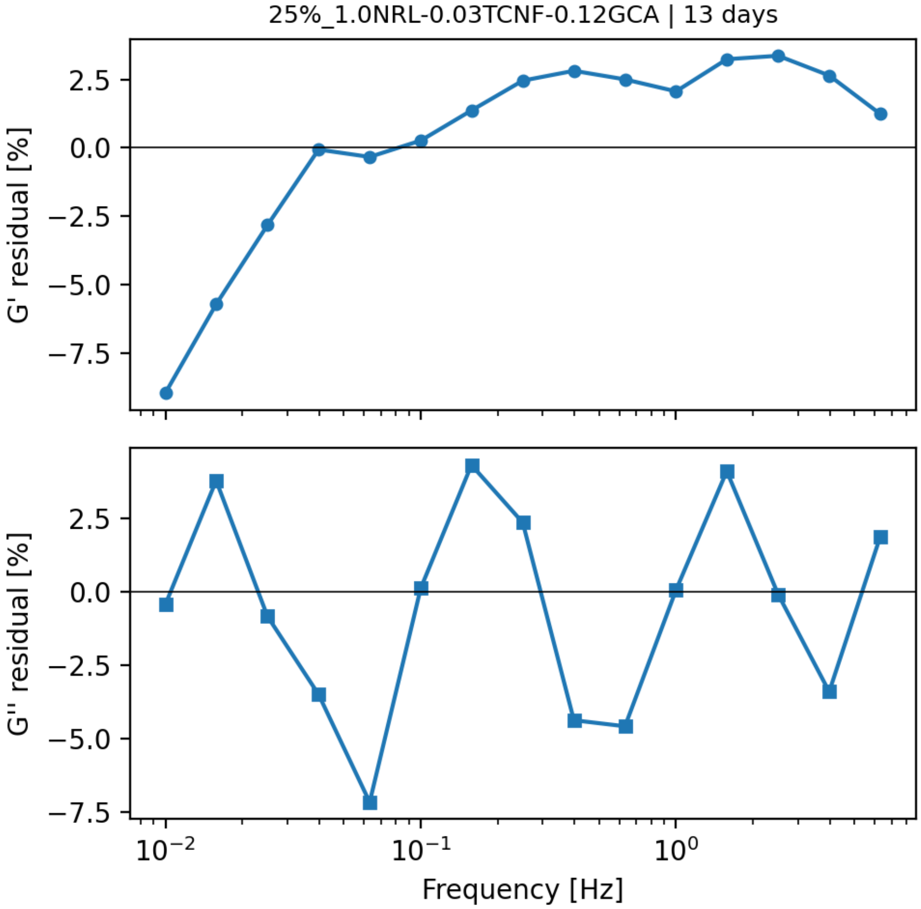
25%_1.0NRL-0.03TCNF-0.12GCA | 13 days
G' residual [%]
G'' residual [%]
2.5
0.0
−2.5
−5.0
−7.5
10^{-2}
10^{-1}
10^{0}
Frequency [Hz]

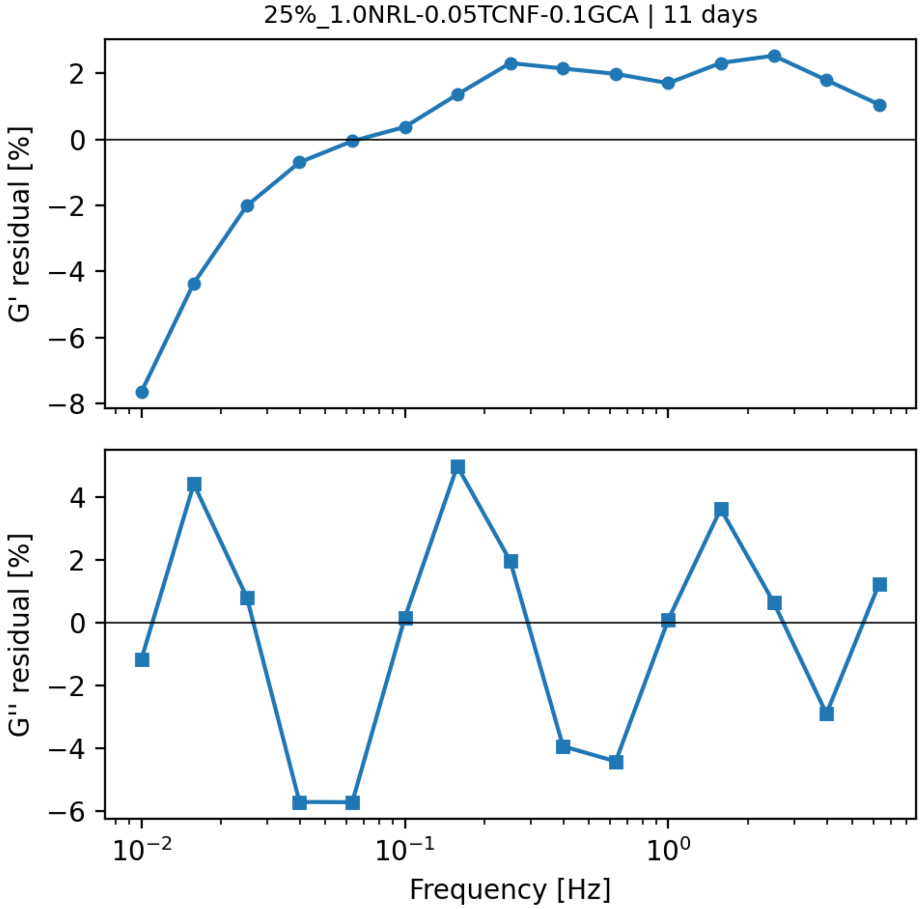

25%_1.0NRL-0.05TCNF-0.1GCA | 11 days
G' residual [%]
G'' residual [%]
Frequency [Hz]

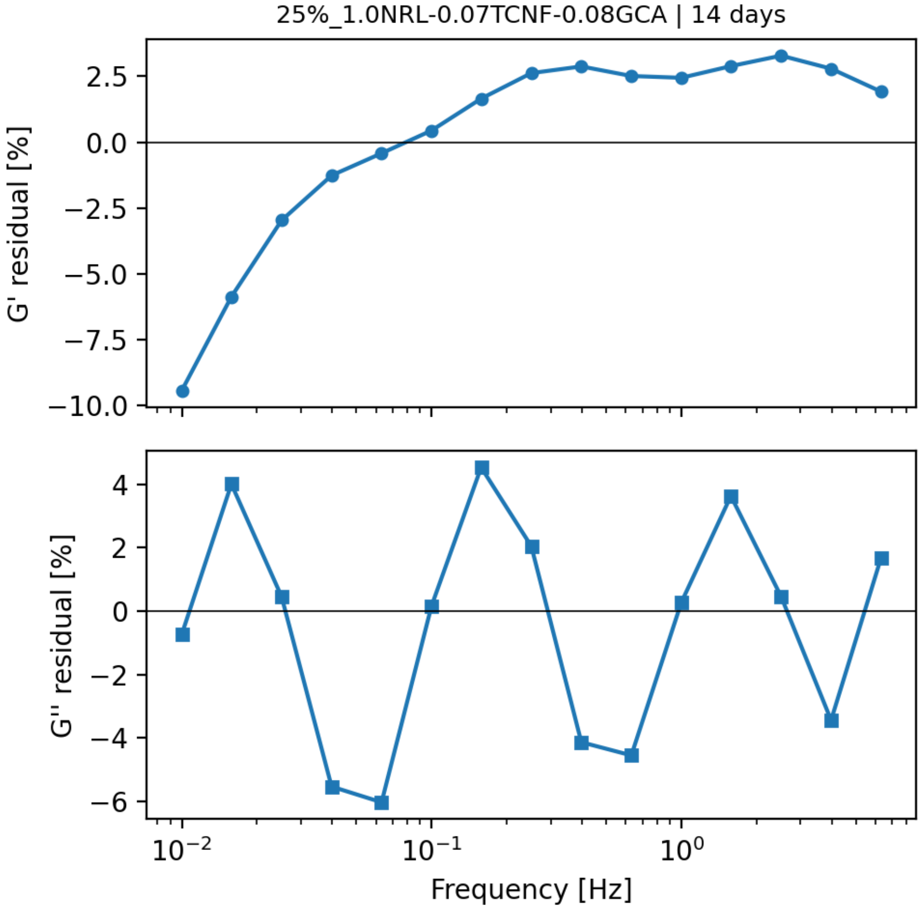

25%_1.0NRL-0.07TCNF-0.08GCA | 14 days
G' residual [%]
2.5
0.0
−2.5
−5.0
−7.5
−10.0
G'' residual [%]
4
2
0
−2
−4
−6
10^{-2}
10^{-1}
10^{0}
Frequency [Hz]

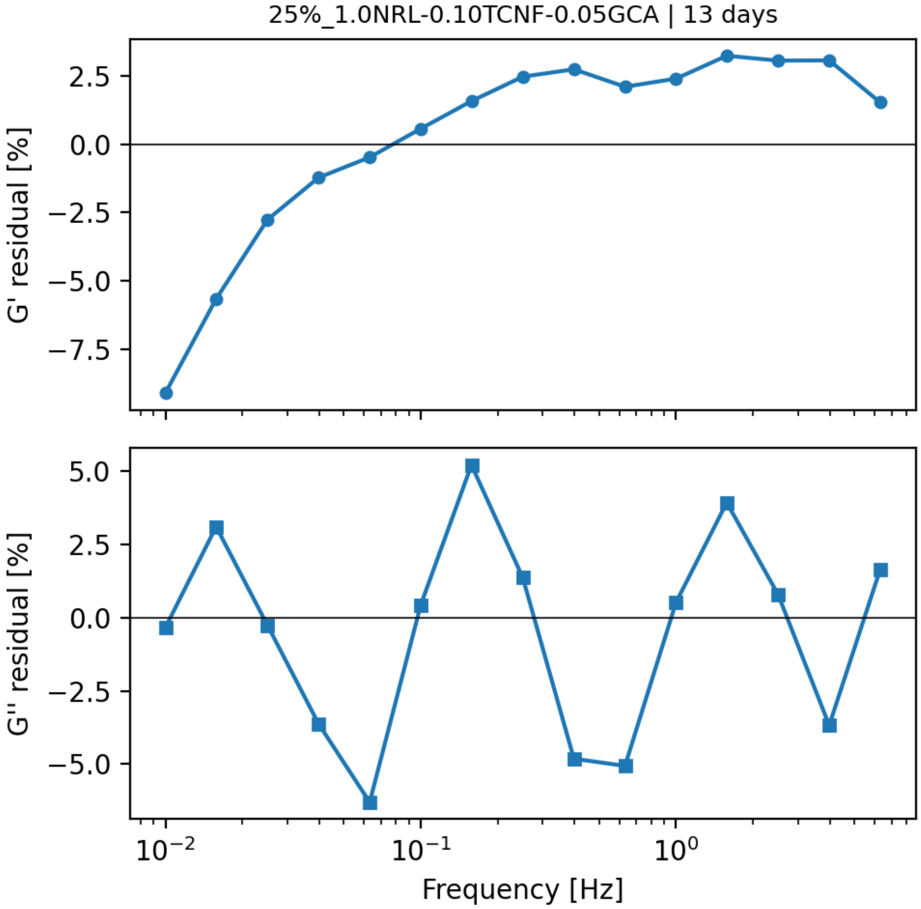

25%_1.0NRL-0.10TCNF-0.05GCA | 13 days
G' residual [%]
2.5
0.0
−2.5
−5.0
−7.5
G'' residual [%]
5.0
2.5
0.0
−2.5
−5.0
10^{-2}
10^{-1}
10^{0}
Frequency [Hz]

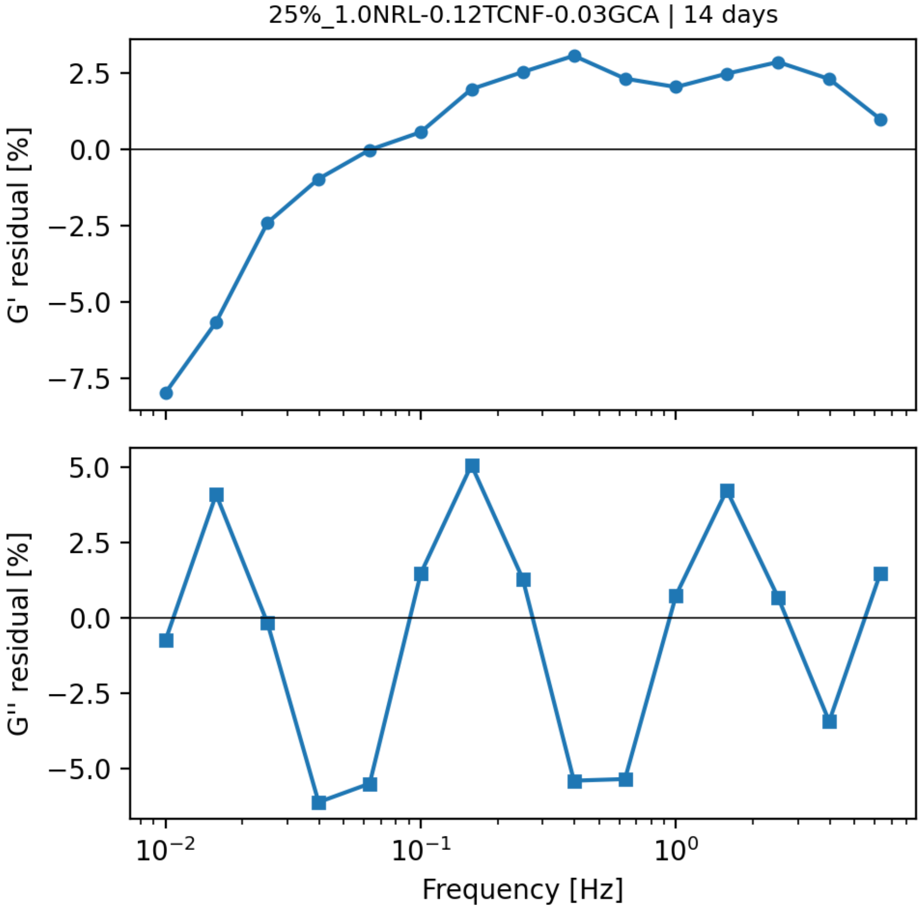
25%_1.0NRL-0.12TCNF-0.03GCA | 14 days
G' residual [%]
2.5
0.0
−2.5
−5.0
−7.5
G'' residual [%]
5.0
2.5
0.0
−2.5
−5.0
10^{-2}
10^{-1}
10^{0}
Frequency [Hz]

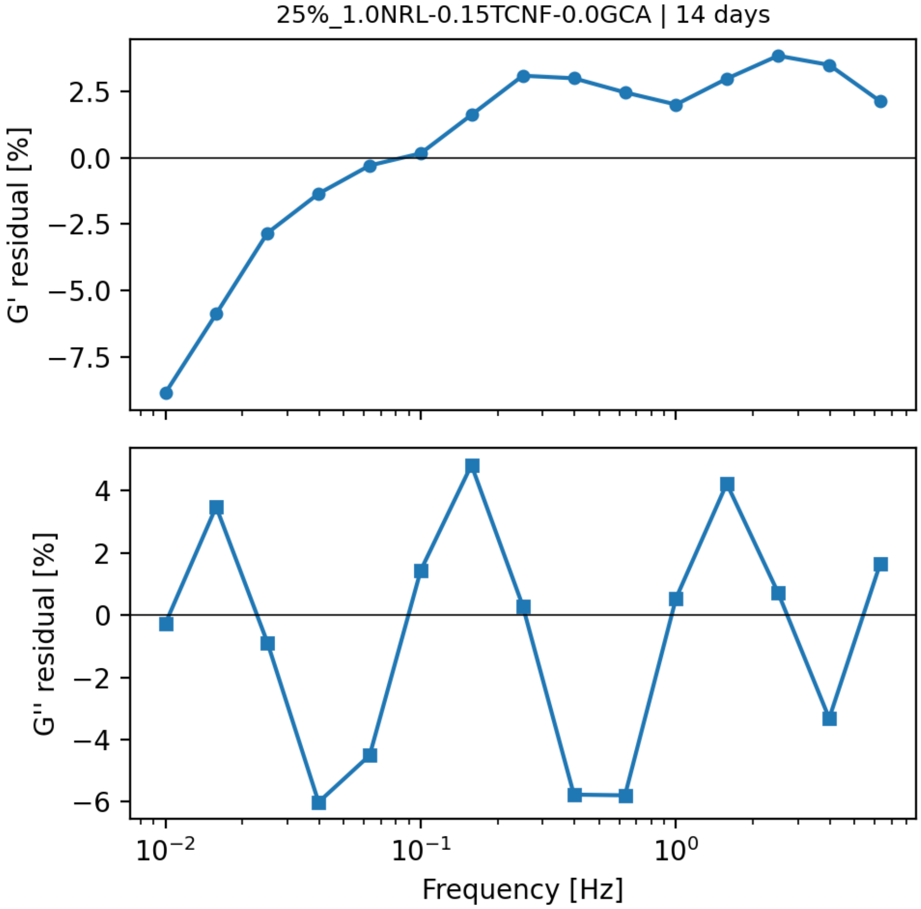

25%_1.0NRL-0.15TCNF-0.0GCA | 14 days
G' residual [%]
2.5
0.0
−2.5
−5.0
−7.5
G'' residual [%]
4
2
0
−2
−4
−6
10^{-2}
10^{-1}
10^{0}
Frequency [Hz]

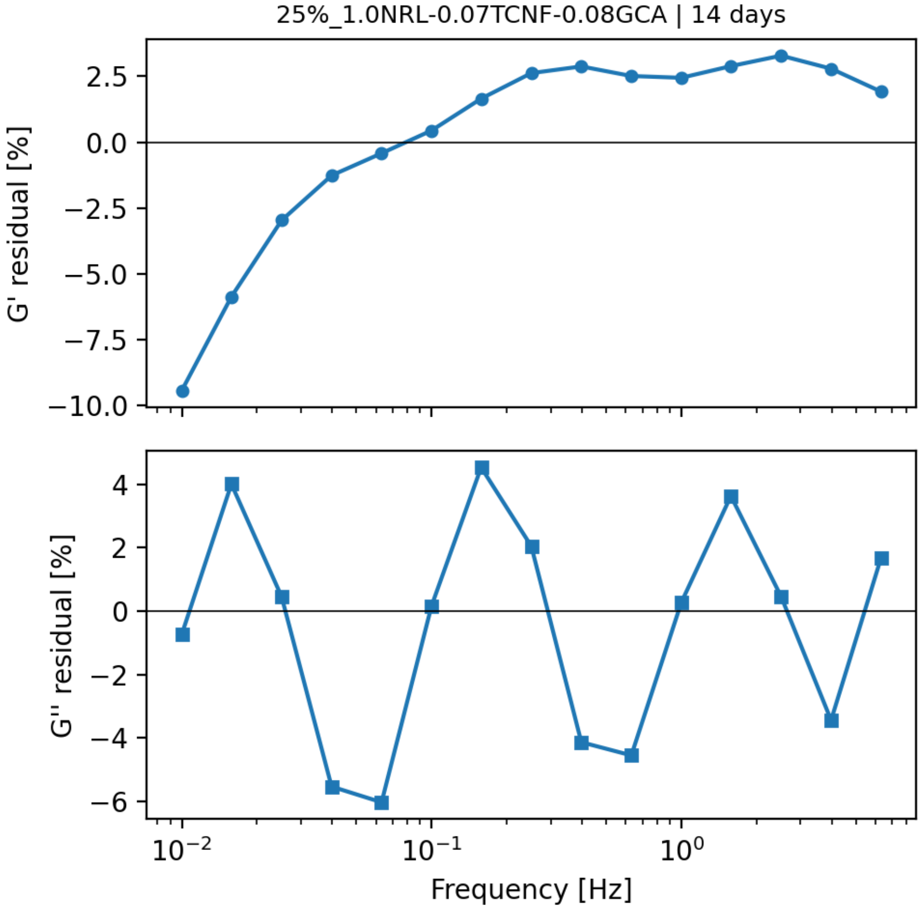

25%_1.0NRL-0.07TCNF-0.08GCA | 14 days
G' residual [%]
2.5
0.0
−2.5
−5.0
−7.5
−10.0
G'' residual [%]
4
2
0
−2
−4
−6
10^{-2}
10^{-1}
10^{0}
Frequency [Hz]

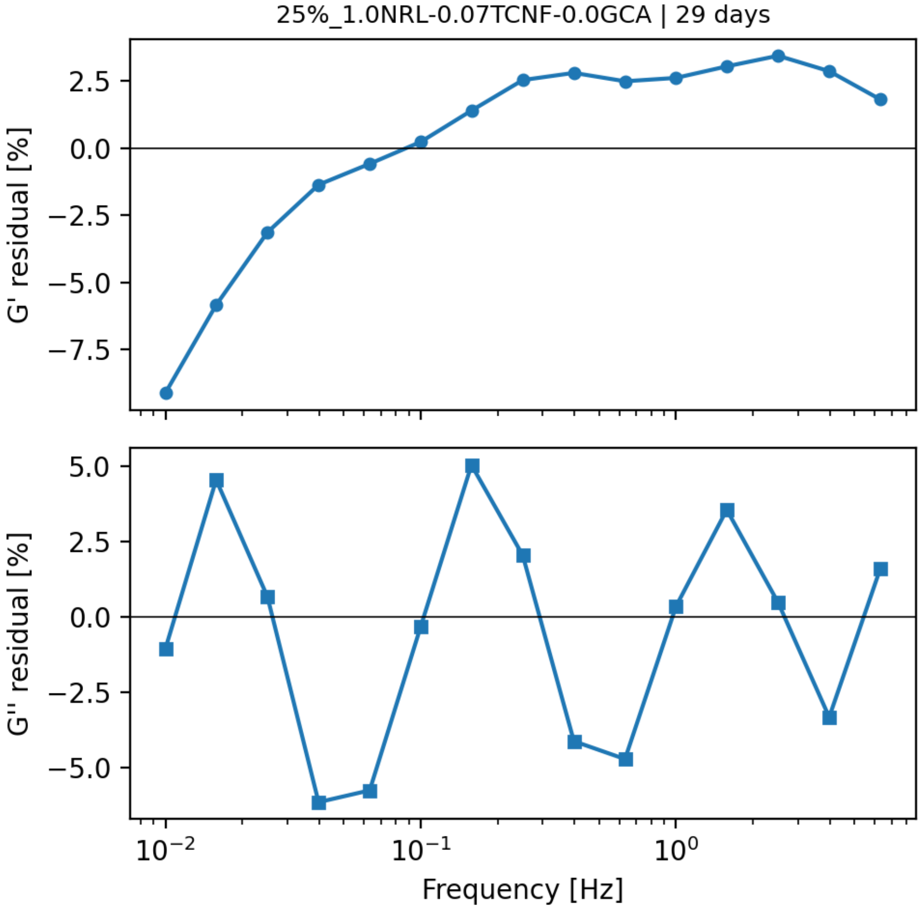

25%_1.0NRL-0.07TCNF-0.0GCA | 29 days
G' residual [%]
G'' residual [%]
Frequency [Hz]

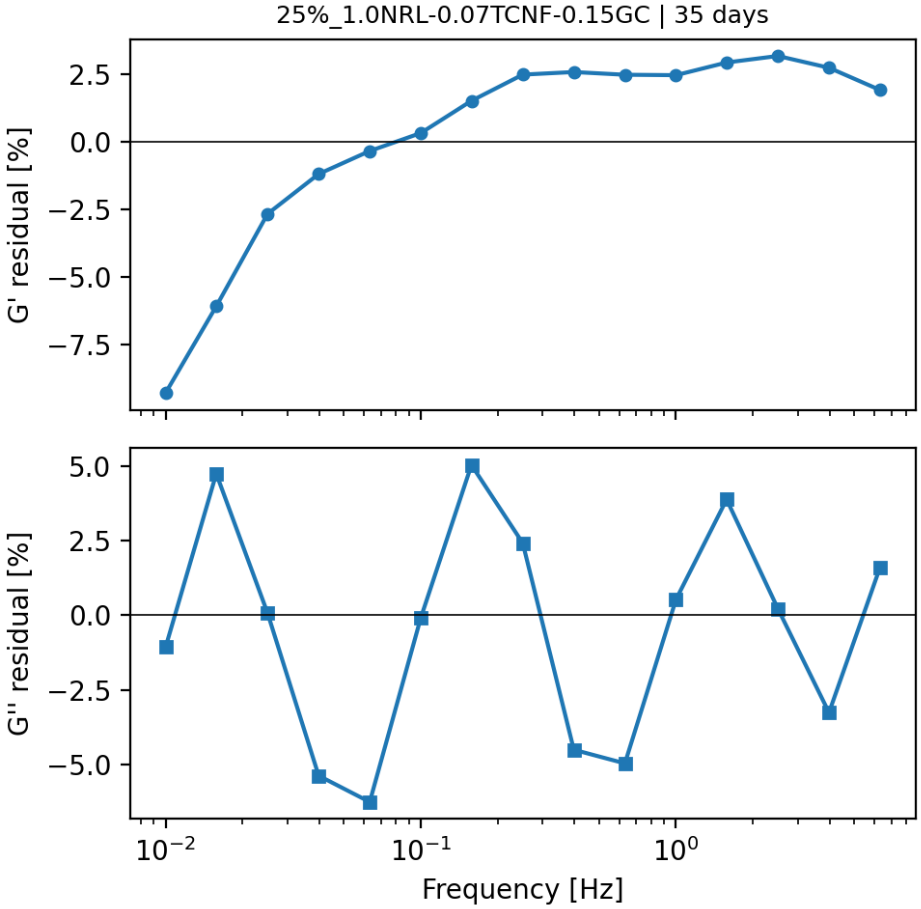

25%_1.0NRL-0.07TCNF-0.15GC | 35 days
G' residual [%]
2.5
0.0
−2.5
−5.0
−7.5
G'' residual [%]
5.0
2.5
0.0
−2.5
−5.0
10^{-2}
10^{-1}
10^{0}
Frequency [Hz]

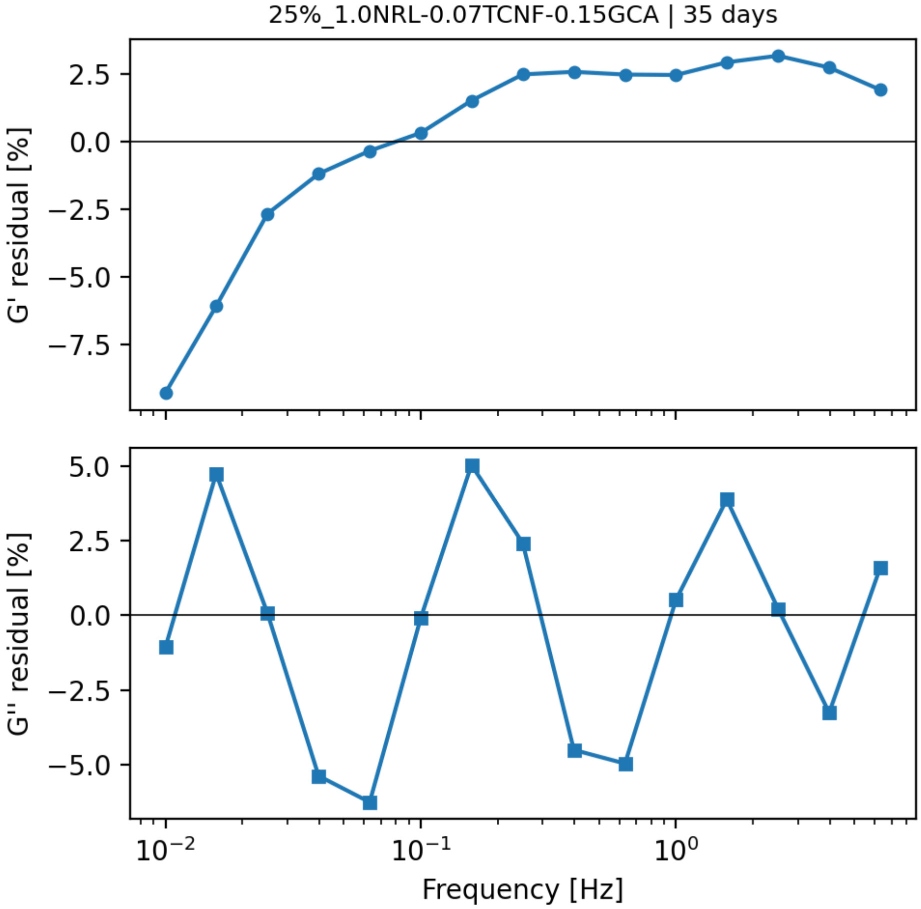

25%_1.0NRL-0.07TCNF-0.15GCA | 35 days
G' residual [%]
2.5
0.0
−2.5
−5.0
−7.5
G'' residual [%]
5.0
2.5
0.0
−2.5
−5.0
10^{-2}
10^{-1}
10^{0}
Frequency [Hz]

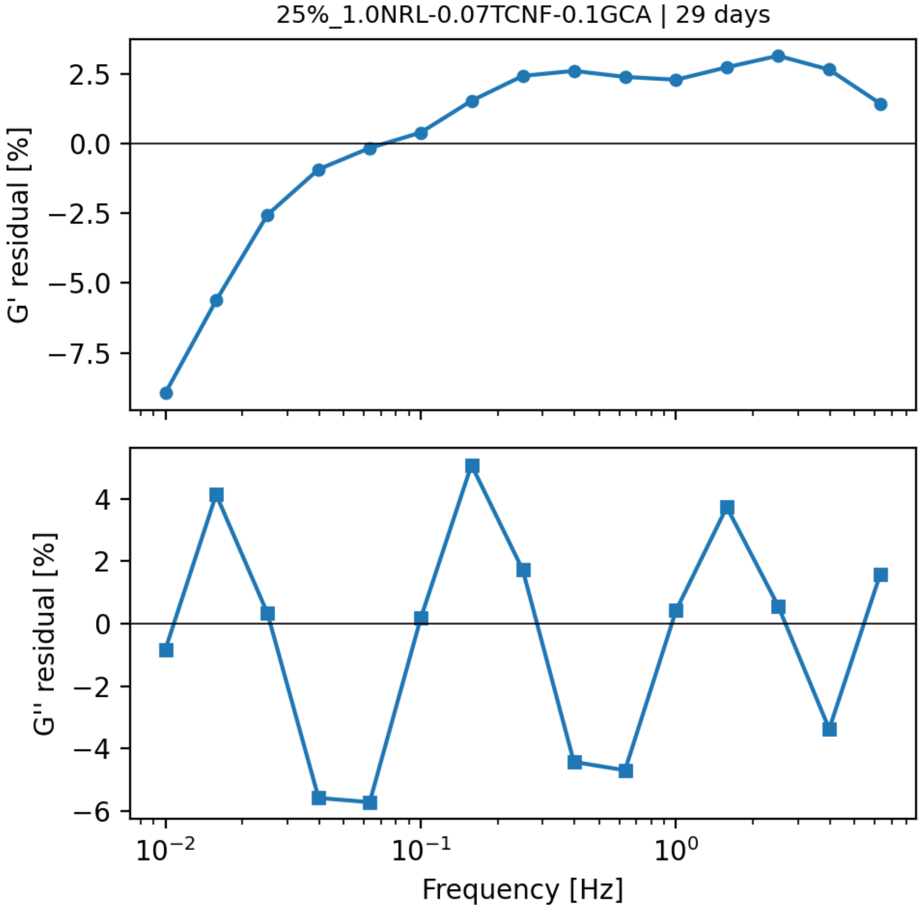
25%_1.0NRL-0.07TCNF-0.1GCA | 29 days
G' residual [%]
2.5
0.0
−2.5
−5.0
−7.5
G'' residual [%]
4
2
0
−2
−4
−6
10^{-2}
10^{-1}
10^{0}
Frequency [Hz]

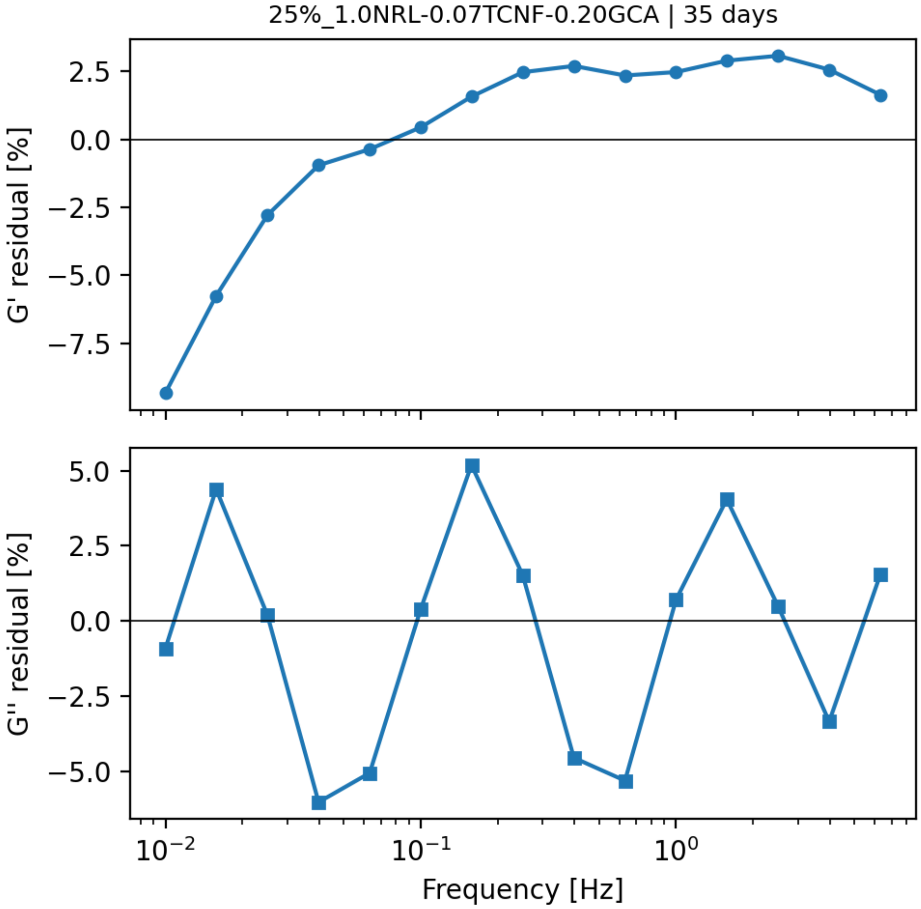

25%_1.0NRL-0.07TCNF-0.20GCA | 35 days
G' residual [%]
2.5
0.0
−2.5
−5.0
−7.5
G'' residual [%]
5.0
2.5
0.0
−2.5
−5.0
10^{-2}
10^{-1}
10^{0}
Frequency [Hz]

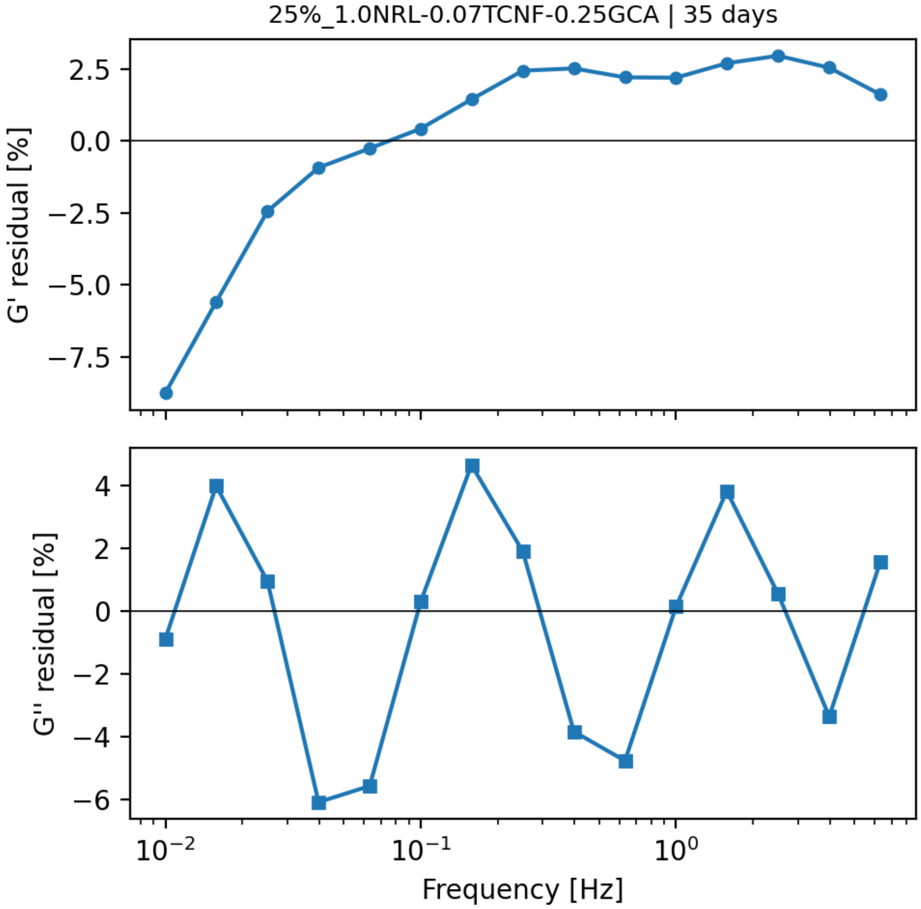

25%_1.0NRL-0.07TCNF-0.25GCA | 35 days
G' residual [%]
2.5
0.0
−2.5
−5.0
−7.5
G'' residual [%]
4
2
0
−2
−4
−6
10^{-2}
10^{-1}
10^{0}
Frequency [Hz]

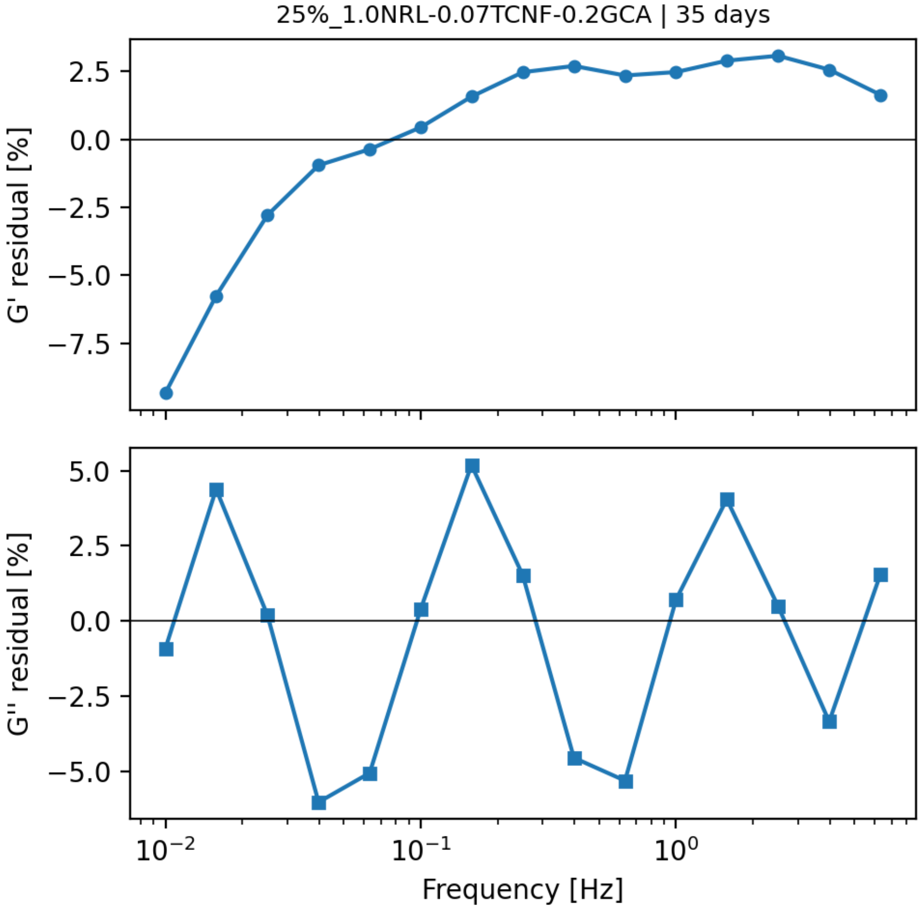

25%_1.0NRL-0.07TCNF-0.2GCA | 35 days
G' residual [%]
2.5
0.0
−2.5
−5.0
−7.5
G'' residual [%]
5.0
2.5
0.0
−2.5
−5.0
10^{-2}
10^{-1}
10^{0}
Frequency [Hz]

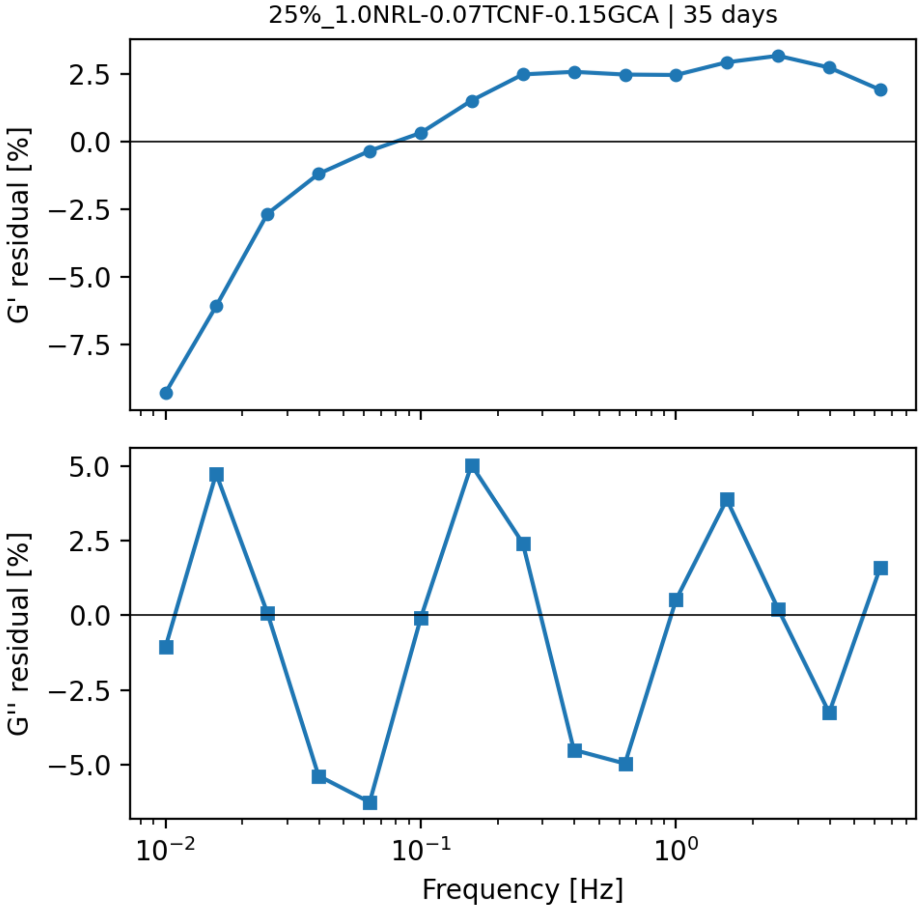

25%_1.0NRL-0.07TCNF-0.15GCA | 35 days
G' residual [%]
G'' residual [%]
2.5
0.0
−2.5
−5.0
−7.5
5.0
2.5
0.0
−2.5
−5.0
10^{-2}
10^{-1}
10^{0}
Frequency [Hz]

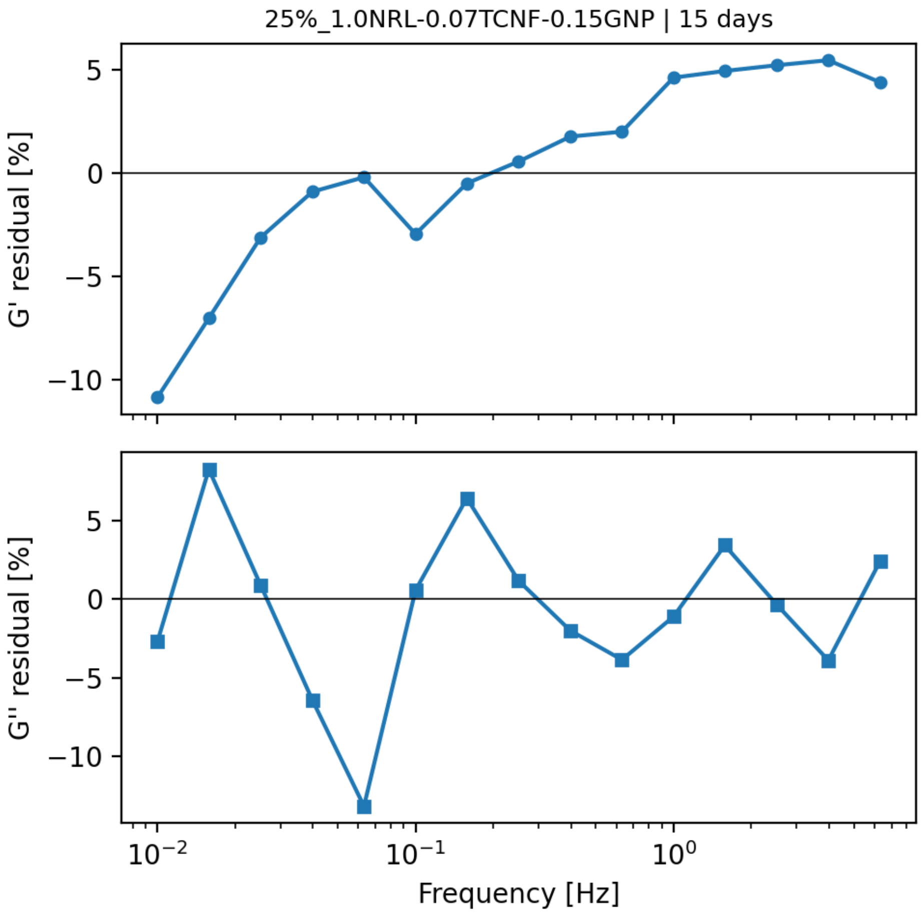

25%_1.0NRL-0.07TCNF-0.15GNP | 15 days
G' residual [%]
G'' residual [%]
5
0
−5
−10
10^{-2}
10^{-1}
10^{0}
Frequency [Hz]

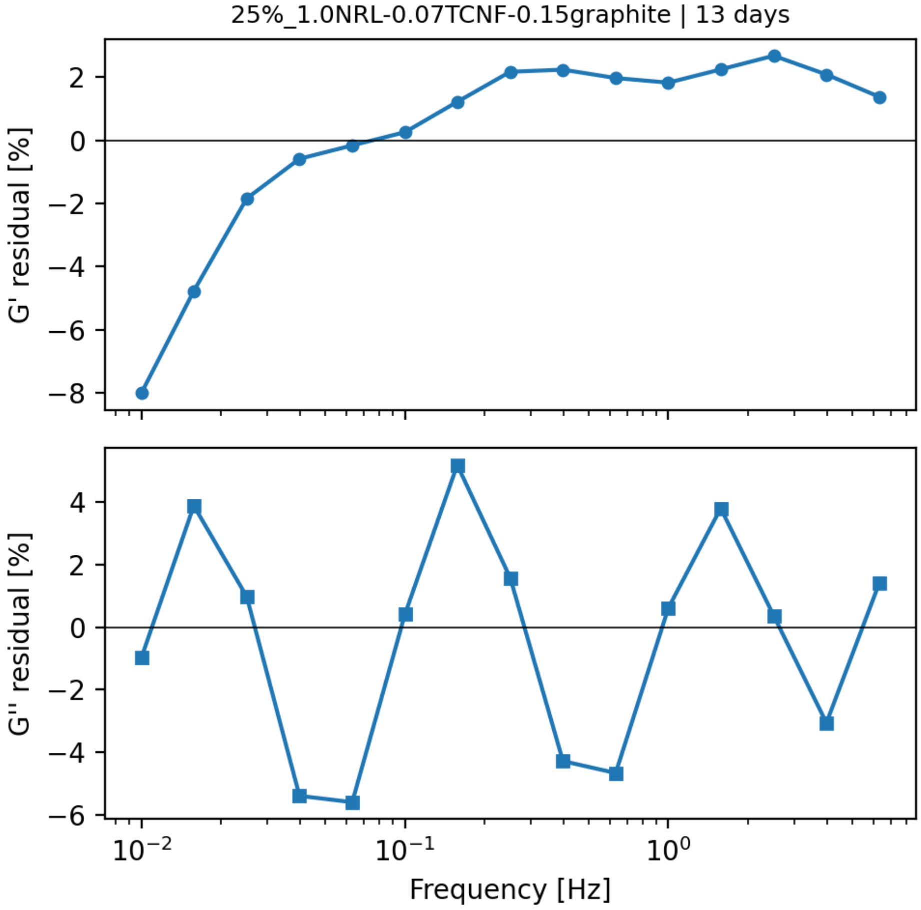

25%_1.0NRL-0.07TCNF-0.15graphite | 13 days
G' residual [%]
G'' residual [%]
Frequency [Hz]
2
0
−2
−4
−6
−8
4
10^{-2}
10^{-1}
10^{0}

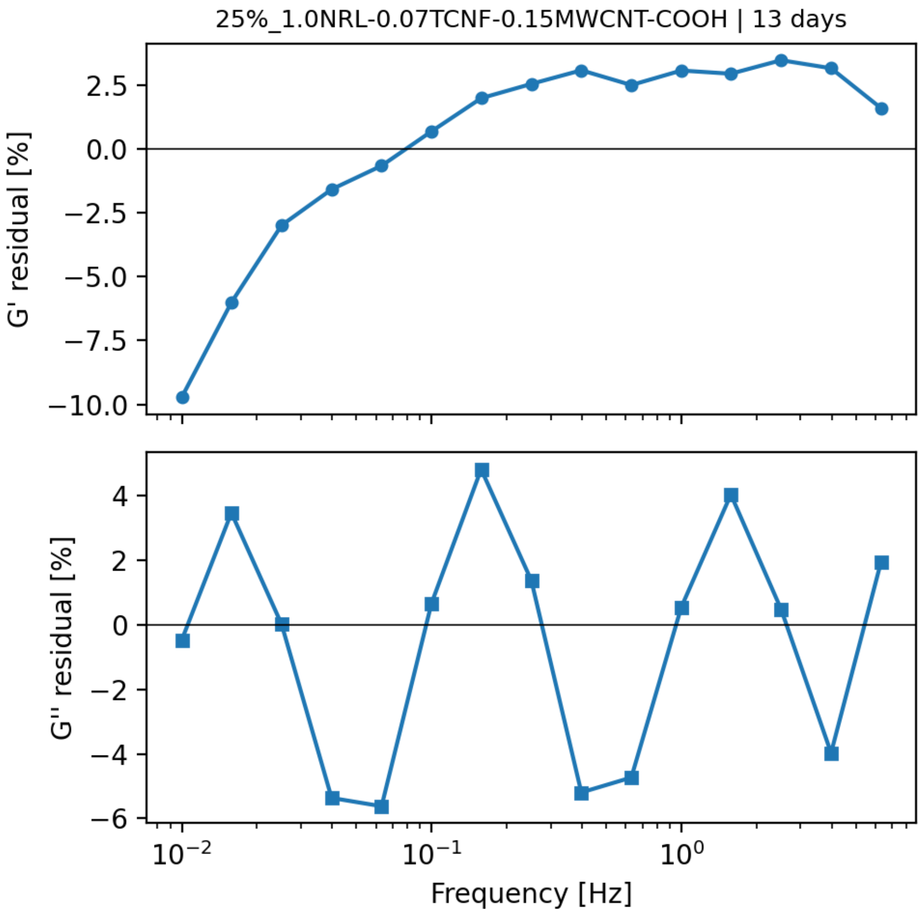
25%_1.0NRL-0.07TCNF-0.15MWCNT-COOH | 13 days
G' residual [%]
2.5
0.0
−2.5
−5.0
−7.5
−10.0
G'' residual [%]
4
2
0
−2
−4
−6
10^{-2}
10^{-1}
10^{0}
Frequency [Hz]

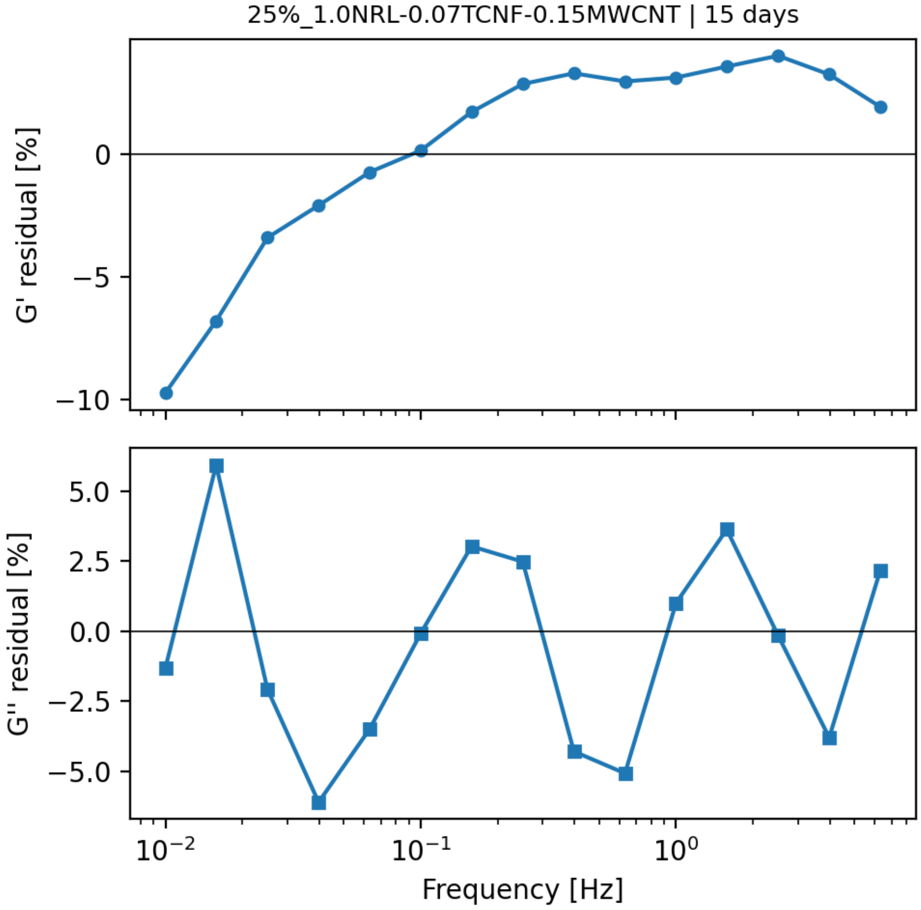

Table S5. Strain-amplitude sweeps and the linear viscoelastic limits.

Sweeps at 1 Hz, strain amplitude 0.001 to 1000% at five points per decade, 20 °C, PP50/P2 at a 1.0 mm gap. The plateau modulus is the median of the points below 1% strain that the instrument resolves, that is, those above the 0.1 μN·m minimum torque. The limit column gives the largest strain at which G′ is still within 5% (or 10%) of that plateau, walking upward from the working strain. Two sweeps were recorded during the measurement campaign and four on 2026-09-08 on retained samples, three of the latter were remixed with a spatula before loading, which destroys the structure built during storage.

Sample	Recorded	Treatment	G′ plateau (Pa)	G′(0.1%) / plateau	tan δ at 0.1%	Torque at 0.1% (μN·m)	γ at 5% departure (%)	γ at 10% departure (%)	Points below torque floor
25 wt%, 1.0:0.03:0.12 GCA	2026-09-08	retained sample, remixed with a spatula before loading	38	0.999	0.144	0.94	6.31	10	6
25 wt%, 1.0:0.07:0 (no additive)	2026-09-08	retained sample, remixed with a spatula before loading	347	1.01	0.103	8.65	2.51	3.98	1
20 wt%, 1.0:0.05:0.10 GCA	2026-09-08	retained sample, remixed with a spatula before loading	31.6	0.998	0.157	0.781	6.31	10	6
25 wt%, 1.0:0.07:0.10 GCA, fresh	campaign	fresh sample, undisturbed	191	1.01	0.119	4.74	2.51	3.98	2
25 wt%, 1.0:0.07:0.10 GCA, 10 months	2026-09-08	same formulation, aged 10 months in the sealed vial, undisturbed	368	1.01	0.0989	9.12	2.51	3.98	2
NRL latex, 61 wt%, fresh	campaign	fresh latex, undisturbed	8.45	1	0.728	0.257	0.681	1	10

Table S6. Cross fits with the shear-thinning exponent unconstrained.

The fits of Table S1 constrain $n \leq 1$, and 97 of 109 curves reach that bound. Refitting without the constraint lowers η_0 by a median of 7% (largest change 29%) and leaves the rankings of Sets 1, 2,

and 4 unchanged. The bounded fits remain the primary description because n > 1 has no physical reading in this model.

Set	Formulation	Age	η0-bounded (Pa·s)	n-bounded	η0-free (Pa·s)	n-free	Δη0 (%)	R2-bounded	R2-free
1st_set	20%_1.0 NRL-0.05TC NF-0.1GCA	1 hour	4.77e+03	0.963	4.77e+03	0.963	9.80e−6	0.999	0.999
1st_set	20%_1.0 NRL-0.05TC NF-0.1GCA	11 days	8.9e+03	1	8.9e+03	1	-0.0172	0.998	0.998
1st_set	20%_1.0 NRL-0.05TC NF-0.1GCA	24 hours (1 day)	6.08e+03	0.975	6.08e+03	0.975	-4.82e−5	0.998	0.998
1st_set	20%_1.0 NRL-0.05TC NF-0.1GCA	4 days	6.76e+03	0.99	6.76e+03	0.99	-8.55e−5	0.998	0.998
1st_set	25%_1.0 NRL-0.05TC NF-0.1GCA	1 hour	1.38e4	0.988	1.38e4	0.988	-5.65e−6	0.999	0.999
1st_set	25%_1.0 NRL-0.05TC NF-0.1GCA	11 days	2.55e4	1	2.36e4	1.04	-7.39	0.998	0.998
1st_set	25%_1.0 NRL-0.05TC NF-0.1GCA	24 hours (1 day)	1.81e4	0.997	1.81e4	0.997	-6.34e−5	0.999	0.999
1st_set	25%_1.0 NRL-0.05TC NF-0.1GCA	7 days	2.24e4	1	2.14e4	1.03	-4.54	0.998	0.998
1st_set	30%_1.0 NRL-0.05TC	1 hour	3.33e4	0.992	3.33e4	0.992	-8.59e−5	0.999	0.999

Set	Formulation	Age	η0-bounded (Pa·s)	n-bounded	η0-free (Pa·s)	n-free	Δη0 (%)	R2-bounded	R2-free
	NF-0.1GCA								
1st_set	30%_1.0 NRL-0.05TC NF-0.1GCA	14 days	5.84e4	1	5.47e4	1.03	-6.35	0.999	0.999
1st_set	30%_1.0 NRL-0.05TC NF-0.1GCA	24 hours (1 day)	3.75e4	1	3.68e4	1.01	-1.86	1	1
1st_set	30%_1.0 NRL-0.05TC NF-0.1GCA	4 days	4.73e4	1	4.48e4	1.02	-5.41	0.999	0.999
1st_set	30%_1.0 NRL-0.05TC NF-0.1GCA	7 days	4.40e4	1	4.22e4	1.02	-4.1	0.999	0.999
1st_set	35%_1.0 NRL-0.05TC NF-0.1GCA	1 hour	7.34e4	1	7.16e4	1.01	-2.53	0.999	0.999
1st_set	35%_1.0 NRL-0.05TC NF-0.1GCA	11 days	9.87e4	1	9.06e4	1.03	-8.22	0.999	1
1st_set	35%_1.0 NRL-0.05TC NF-0.1GCA	24 hours (1 day)	7.84e4	1	7.57e4	1.01	-3.42	1	1
1st_set	35%_1.0 NRL-0.05TC NF-0.1GCA	7 days	9.69e4	1	9.15e4	1.02	-5.63	1	1
1st_set	40%_1.0 NRL-0.05TC NF-0.1GCA	1 hour	7.32e4	1	7.22e4	1	-1.35	1	1
1st_set	40%_1.0 NRL-	14 days	1.01e5	1	9.41e4	1.02	-6.82	1	1

Set	Formulation	Age	η0-bounded (Pa·s)	n-bounded	η0-free (Pa·s)	n-free	Δη0 (%)	R2-bounded	R2-free
	0.05TC NF-0.1GCA								
1st_set	40%_1.0 NRL-0.05TC NF-0.1GCA	24 hours (1 day)	7.67e4	1	7.31e4	1.02	-4.58	1	1
1st_set	40%_1.0 NRL-0.05TC NF-0.1GCA	4 days	8.17e4	1	7.45e4	1.03	-8.83	1	1
1st_set	40%_1.0 NRL-0.05TC NF-0.1GCA	7 days	8.99e4	1	8.49e4	1.02	-5.61	1	1
1st_set	40%_1.0 NRL-0.05TC NF-0.1GCA	9 days	1.01e5	1	9.43e4	1.02	-6.35	1	1
2nd_set	25%_1.0 NRL-0.03TC NF-0.12GCA	1 hour	4.08e+03	0.972	4.08e+03	0.972	-2.03e−5	0.999	0.999
2nd_set	25%_1.0 NRL-0.03TC NF-0.12GCA	13 days	9.64e+03	1	9.63e+03	1	-0.159	0.999	0.999
2nd_set	25%_1.0 NRL-0.03TC NF-0.12GCA	24 hours (1 day)	5.34e+03	0.983	5.34e+03	0.983	-1.45e−4	0.999	0.999
2nd_set	25%_1.0 NRL-0.03TC NF-0.12GCA	3 days	6.86e+03	0.989	6.86e+03	0.989	-6.65e−5	0.999	0.999
2nd_set	25%_1.0 NRL-0.03TC	6 days	9.16e+03	1	8.92e+03	1.01	-2.57	0.998	0.998

Set	Formulation	Age	η0-bounded (Pa·s)	n-bounded	η0-free (Pa·s)	n-free	Δη0 (%)	R2-bounded	R2-free
	NF-0.12GCA								
2nd_set	25%_1.0 NRL-0.05TC NF-0.1GCA	1 hour	1.38e4	0.988	1.38e4	0.988	-5.65e−6	0.999	0.999
2nd_set	25%_1.0 NRL-0.05TC NF-0.1GCA	11 days	2.55e4	1	2.36e4	1.04	-7.39	0.998	0.998
2nd_set	25%_1.0 NRL-0.05TC NF-0.1GCA	24 hours (1 day)	1.81e4	0.997	1.81e4	0.997	-6.34e−5	0.999	0.999
2nd_set	25%_1.0 NRL-0.05TC NF-0.1GCA	7 days	2.24e4	1	2.14e4	1.03	-4.54	0.998	0.998
2nd_set	25%_1.0 NRL-0.07TC NF-0.08GCA	1 hour	3.39e4	1	3.36e4	1	-0.62	0.999	0.999
2nd_set	25%_1.0 NRL-0.07TC NF-0.08GCA	14 days	5.41e4	1	4.89e4	1.05	-9.74	0.998	0.998
2nd_set	25%_1.0 NRL-0.07TC NF-0.08GCA	24 hours (1 day)	3.70e4	1	3.61e4	1.01	-2.63	0.999	0.999
2nd_set	25%_1.0 NRL-0.07TC NF-0.08GCA	3 days	4.06e4	1	3.79e4	1.03	-6.52	0.998	0.999
2nd_set	25%_1.0 NRL-0.07TC	7 days	5.07e4	1	4.65e4	1.04	-8.44	0.998	0.998

Set	Formulation	Age	η0-bounded (Pa·s)	n-bounded	η0-free (Pa·s)	n-free	Δη0 (%)	R2-bounded	R2-free
	NF-0.08GCA								
2nd_set	25%_1.0NRL-0.10TCNF-0.05GCA	1 hour	7.50e4	1	7.17e4	1.02	-4.33	0.999	0.999
2nd_set	25%_1.0NRL-0.10TCNF-0.05GCA	13 days	1.11e5	1	9.86e4	1.04	-11.4	0.999	1
2nd_set	25%_1.0NRL-0.10TCNF-0.05GCA	24 hours (1 day)	7.72e4	1	7.14e4	1.04	-7.57	0.999	1
2nd_set	25%_1.0NRL-0.10TCNF-0.05GCA	3 days	7.61e4	1	7.11e4	1.03	-6.51	0.999	1
2nd_set	25%_1.0NRL-0.10TCNF-0.05GCA	6 days	8.10e4	1	7.53e4	1.03	-7.03	0.999	1
2nd_set	25%_1.0NRL-0.12TCNF-0.03GCA	1 hour	8.20e4	1	7.12e4	1.06	-13.2	0.999	1
2nd_set	25%_1.0NRL-0.12TCNF-0.03GCA	14 days	1.39e5	1	1.17e5	1.07	-15.8	0.999	1
2nd_set	25%_1.0NRL-0.12TCNF-	24 hours (1 day)	8.87e4	1	7.88e4	1.05	-11.1	0.999	1

Set	Formulation	Age	η0-bounded (Pa·s)	n-bounded	η0-free (Pa·s)	n-free	Δη0 (%)	R2-bounded	R2-free
	0.03GCA								
2nd_set	25%_1.0NRL-0.12TCNF-0.03GCA	3 days	1.07e5	1	9.20e4	1.07	-14.3	0.999	1
2nd_set	25%_1.0NRL-0.12TCNF-0.03GCA	7 days	1.20e5	1	1.07e5	1.05	-10.8	0.999	1
2nd_set	25%_1.0NRL-0.15TCNF-0.0GCA	1 hour	1.33e5	1	1.14e5	1.06	-14.3	0.999	1
2nd_set	25%_1.0NRL-0.15TCNF-0.0GCA	14 days	1.98e5	1	1.65e5	1.07	-16.8	0.999	1
2nd_set	25%_1.0NRL-0.15TCNF-0.0GCA	24 hours (1 day)	1.43e5	1	1.21e5	1.07	-15.7	0.999	1
2nd_set	25%_1.0NRL-0.15TCNF-0.0GCA	4 days	1.68e5	1	1.43e5	1.06	-15	0.999	1
2nd_set	25%_1.0NRL-0.15TCNF-0.0GCA	7 days	1.77e5	1	1.53e5	1.06	-13.6	0.999	1
3rd_set	25%_1.0NRL-0.07TCNF-0.08GCA	1 hour	3.39e4	1	3.36e4	1	-0.62	0.999	0.999
3rd_set	25%_1.0NRL-0.07TCNF-	14 days	5.41e4	1	4.89e4	1.05	-9.74	0.998	0.998

Set	Formulation	Age	η0-bounded (Pa·s)	n-bounded	η0-free (Pa·s)	n-free	Δη0 (%)	R2-bounded	R2-free
	0.08GCA								
3rd_set	25%_1.0NRL-0.07TCNF-0.08GCA	24 hours (1 day)	3.70e4	1	3.61e4	1.01	-2.63	0.999	0.999
3rd_set	25%_1.0NRL-0.07TCNF-0.08GCA	3 days	4.06e4	1	3.79e4	1.03	-6.52	0.998	0.999
3rd_set	25%_1.0NRL-0.07TCNF-0.08GCA	7 days	5.07e4	1	4.65e4	1.04	-8.44	0.998	0.998
3rd_set	25%_1.0NRL-0.07TCNF-0.0GCA	1 hour	3.48e4	0.993	3.48e4	0.993	-4.07e−6	0.999	0.999
3rd_set	25%_1.0NRL-0.07TCNF-0.0GCA	24 hours (1 day)	4.25e4	1	4.18e4	1.01	-1.69	0.999	0.999
3rd_set	25%_1.0NRL-0.07TCNF-0.0GCA	29 days	6.76e4	1	6.20e4	1.04	-8.22	0.998	0.998
3rd_set	25%_1.0NRL-0.07TCNF-0.0GCA	3 days	4.65e4	1	4.48e4	1.02	-3.67	0.999	0.999
3rd_set	25%_1.0NRL-0.07TCNF-0.0GCA	7 days	5.79e4	1	5.63e4	1.01	-2.67	0.999	0.999
3rd_set	25%_1.0NRL-0.07TCNF-0.1GCA	1 hour	3.11e4	1	3.03e4	1.01	-2.59	0.999	0.999

Set	Formulation	Age	η0-bounded (Pa·s)	n-bounded	η0-free (Pa·s)	n-free	Δη0 (%)	R2-bounded	R2-free
3rd_set	25%_1.0NRL-0.07TCNF-0.1GCA	24 hours	3.87e4	1	3.75e4	1.01	-2.95	0.999	0.999
3rd_set	25%_1.0NRL-0.07TCNF-0.1GCA	29 days	4.86e4	1	4.42e4	1.04	-9.02	0.999	0.999
3rd_set	25%_1.0NRL-0.07TCNF-0.1GCA	3 days	4.12e4	1	3.73e4	1.05	-9.57	0.999	0.999
3rd_set	25%_1.0NRL-0.07TCNF-0.1GCA	7 days	4.42e4	1	4.17e4	1.03	-5.56	0.999	0.999
3rd_set	25%_1.0NRL-0.07TCNF-0.15GCA	1 hour	3.29e4	1	3.17e4	1.02	-3.49	0.999	0.999
3rd_set	25%_1.0NRL-0.07TCNF-0.15GCA	24 hours (1 day)	3.62e4	1	3.38e4	1.03	-6.59	0.999	0.999
3rd_set	25%_1.0NRL-0.07TCNF-0.15GCA	3 days	4.01e4	1	3.66e4	1.05	-8.62	0.998	0.998
3rd_set	25%_1.0NRL-0.07TCNF-0.15GCA	35 days	5.79e4	1	4.99e4	1.08	-13.8	0.997	0.998
3rd_set	25%_1.0NRL-0.07TCNF-0.15GCA	6 days	4.37e4	1	3.91e4	1.06	-10.4	0.997	0.998

Set	Formulation	Age	η0-bounded (Pa·s)	n-bounded	η0-free (Pa·s)	n-free	Δη0 (%)	R2-bounded	R2-free
3rd_set	25%_1.0NRL-0.07TCNF-0.2GCA	1 hour	3.28e4	1	3.04e4	1.03	-7.31	0.999	0.999
3rd_set	25%_1.0NRL-0.07TCNF-0.2GCA	24 hours (1 day)	3.49e4	1	3.21e4	1.04	-8.07	0.998	0.999
3rd_set	25%_1.0NRL-0.07TCNF-0.2GCA	3 days	3.81e4	1	3.43e4	1.06	-9.9	0.997	0.998
3rd_set	25%_1.0NRL-0.07TCNF-0.2GCA	35 days	5.88e4	1	4.83e4	1.11	-17.8	0.995	0.997
3rd_set	25%_1.0NRL-0.07TCNF-0.2GCA	6 days	4.47e4	1	3.97e4	1.06	-11.3	0.997	0.998
3rd_set	25%_1.0NRL-0.07TCNF-0.25GCA	1 hour	2.66e4	1	2.52e4	1.02	-5.22	0.999	0.999
3rd_set	25%_1.0NRL-0.07TCNF-0.25GCA	24 hours (1 day)	2.84e4	1	2.65e4	1.04	-6.91	0.998	0.998
3rd_set	25%_1.0NRL-0.07TCNF-0.25GCA	3 days	3.48e4	1	3.06e4	1.07	-12.3	0.997	0.998
3rd_set	25%_1.0NRL-0.07TCNF-0.25GCA	35 days	5.14e4	1	4.20e4	1.11	-18.3	0.994	0.996

Set	Formulation	Age	η0-bounded (Pa·s)	n-bounded	η0-free (Pa·s)	n-free	Δη0 (%)	R2-bounded	R2-free
3rd_set	25%_1.0NRL-0.07TCNF-0.25GCA	6 days	3.87e4	1	3.34e4	1.08	-13.8	0.996	0.997
4th_set	25%_1.0NRL-0.07TCNF-0.15GCA	1 hour	3.29e4	1	3.17e4	1.02	-3.49	0.999	0.999
4th_set	25%_1.0NRL-0.07TCNF-0.15GCA	24 hours (1 day)	3.62e4	1	3.38e4	1.03	-6.59	0.999	0.999
4th_set	25%_1.0NRL-0.07TCNF-0.15GCA	3 days	4.01e4	1	3.66e4	1.05	-8.62	0.998	0.998
4th_set	25%_1.0NRL-0.07TCNF-0.15GCA	35 days	5.79e4	1	4.99e4	1.08	-13.8	0.997	0.998
4th_set	25%_1.0NRL-0.07TCNF-0.15GCA	6 days	4.37e4	1	3.91e4	1.06	-10.4	0.997	0.998
4th_set	25%_1.0NRL-0.07TCNF-0.15GNP	1 hour	1.02e5	1	7.79e4	1.13	-23.6	0.993	0.998
4th_set	25%_1.0NRL-0.07TCNF-0.15GNP	10 days	1.63e5	1	1.31e5	1.1	-20	0.997	0.999
4th_set	25%_1.0NRL-	15 days	1.70e5	1	1.64e5	1.02	-3.92	0.995	0.995

Set	Formulation	Age	η0-bounded (Pa·s)	n-bounded	η0-free (Pa·s)	n-free	Δη0 (%)	R2-bounded	R2-free
	0.07TCNF-0.15GNP								
4th_set	25%_1.0NRL-0.07TCNF-0.15GNP	24 hours (1 day)	1.32e5	1	9.81e4	1.14	-26	0.994	0.999
4th_set	25%_1.0NRL-0.07TCNF-0.15GNP	3 days	1.50e5	1	1.18e5	1.11	-21.6	0.996	0.999
4th_set	25%_1.0NRL-0.07TCNF-0.15GNP	6 days	1.53e5	1	1.22e5	1.11	-20.7	0.997	0.999
4th_set	25%_1.0NRL-0.07TCNF-0.15graphite	1 hour	2.87e4	1	2.78e4	1.02	-3.42	0.999	0.999
4th_set	25%_1.0NRL-0.07TCNF-0.15graphite	14 days	4.67e4	1	4.11e4	1.07	-12	0.997	0.998
4th_set	25%_1.0NRL-0.07TCNF-0.15graphite	24 hours (1 day)	3.30e4	1	3.10e4	1.03	-6.17	0.999	0.999
4th_set	25%_1.0NRL-0.07TCNF-0.15graphite	4 days	4.01e4	1	3.76e4	1.03	-6.13	0.997	0.998
4th_set	25%_1.0NRL-0.07TCNF-	8 days	4.74e4	1	4.39e4	1.04	-7.54	0.998	0.998

Set	Formulation	Age	η0-bounded (Pa·s)	n-bounded	η0-free (Pa·s)	n-free	Δη0 (%)	R2-bounded	R2-free
	0.15graphite								
4th_set	25%_1.0NRL-0.07TCNF-0.15MWCNT-COOH	1 hour	1.29e5	1	1.03e5	1.1	-20.1	0.997	1
4th_set	25%_1.0NRL-0.07TCNF-0.15MWCNT-COOH	13 days	1.62e5	1	1.37e5	1.07	-15.8	0.998	1
4th_set	25%_1.0NRL-0.07TCNF-0.15MWCNT-COOH	24 hours (1 day)	1.21e5	1	1.03e5	1.07	-15.2	0.999	1
4th_set	25%_1.0NRL-0.07TCNF-0.15MWCNT-COOH	4 days	1.44e5	1	1.22e5	1.07	-14.9	0.999	1
4th_set	25%_1.0NRL-0.07TCNF-0.15MWCNT-COOH	8 days	1.58e5	1	1.35e5	1.07	-14.7	0.999	1
4th_set	25%_1.0NRL-0.07TCNF-0.15MWCNT	1 hour	1.05e5	1	9.19e4	1.06	-12.6	0.999	1
4th_set	25%_1.0NRL-0.07TCNF-0.15MWCNT	10 days	1.60e5	1	1.45e5	1.04	-9.68	0.999	0.999

Set	Formulation	Age	η0-bounded (Pa·s)	n-bounded	η0-free (Pa·s)	n-free	Δη0 (%)	R2-bounded	R2-free
4th_set	25%_1.0 NRL-0.07TCNF-0.15MWCNT	15 days	3.38e5	1	2.40e5	1.12	-29.1	0.996	0.999
4th_set	25%_1.0 NRL-0.07TCNF-0.15MWCNT	24 hours (1 day)	1.06e5	1	9.47e4	1.05	-10.8	0.999	1
4th_set	25%_1.0 NRL-0.07TCNF-0.15MWCNT	3 days	1.37e5	1	1.21e5	1.05	-11.6	0.999	1
4th_set	25%_1.0 NRL-0.07TCNF-0.15MWCNT	6 days	1.49e5	1	1.32e5	1.05	-11.6	0.999	1

Table S7. Strain accumulated in the flow protocol.

The measuring time per point falls logarithmically from 120 s to 1 s over the 41 points of the ramp, so the strain accumulated at a given point is the product of that time and the measured shear rate. The first retained point is the fourth point of the ramp, the first three being discarded (Section 4.3). The rate at which the cumulative strain first reaches 2%, a value of the order of the linear-viscoelastic limit of these materials (Table S5), marks where the material has been deformed far enough for a steady flow state to be plausible.

Formulation	Set	Age	First retained rate (1/s)	Strain in that point (%)	Cumulative strain (%)	Strain at 1e−3 1/s (%)	Cumulative at 1e−3 1/s (%)	Rate at 2% cumulative (1/s)
20%_1.0 NRL-0.05TCN	1st_set	1 hour	3.62e−5	0.303	0.741	3.58	11.9	1.52e−4

Formulation	Set	Age	First retained rate (1/s)	Strain in that point (%)	Cumulative strain (%)	Strain at 1e−3 1/s (%)	Cumulative at 1e−3 1/s (%)	Rate at 2% cumulative (1/s)
F-0.1GCA								
20%_1.0 NRL-0.05TCNF-0.1GCA	1st_set	24 hours (1 day)	3.60e−5	0.302	0.723	3.62	11.9	1.52e−4
20%_1.0 NRL-0.05TCNF-0.1GCA	1st_set	4 days	3.49e−5	0.292	0.723	3.63	11.9	1.51e−4
20%_1.0 NRL-0.05TCNF-0.1GCA	1st_set	11 days	3.43e−5	0.287	0.706	3.63	11.9	1.50e−4
25%_1.0 NRL-0.05TCNF-0.1GCA	1st_set	1 hour	3.39e−5	0.284	0.668	3.63	11.9	1.50e−4
25%_1.0 NRL-0.05TCNF-0.1GCA	1st_set	24 hours (1 day)	3.38e−5	0.283	0.64	3.63	11.9	1.50e−4
25%_1.0 NRL-0.05TCNF-0.1GCA	1st_set	7 days	3.23e−5	0.271	0.635	3.63	11.9	1.49e−4
25%_1.0 NRL-0.05TCNF-0.1GCA	1st_set	11 days	3.21e−5	0.269	0.615	3.63	11.8	1.49e−4
30%_1.0 NRL-0.05TCNF-0.1GCA	1st_set	1 hour	3.24e−5	0.272	0.603	3.63	11.9	1.59e−4
30%_1.0 NRL-0.05TCNF-0.1GCA	1st_set	24 hours (1 day)	3.12e−5	0.261	0.588	3.63	11.9	1.58e−4

Formulation	Set	Age	First retained rate (1/s)	Strain in that point (%)	Cumulative strain (%)	Strain at 1e−3 1/s (%)	Cumulative at 1e−3 1/s (%)	Rate at 2% cumulative (1/s)
30%_1.0 NRL-0.05TCNF-0.1GCA	1st_set	4 days	3.21e−5	0.269	0.554	3.63	11.9	1.58e−4
30%_1.0 NRL-0.05TCNF-0.1GCA	1st_set	7 days	2.97e−5	0.249	0.554	3.63	11.8	1.58e−4
30%_1.0 NRL-0.05TCNF-0.1GCA	1st_set	14 days	3.15e−5	0.264	0.549	3.63	11.9	1.58e−4
35%_1.0 NRL-0.05TCNF-0.1GCA	1st_set	1 hour	3.06e−5	0.256	0.529	3.63	11.8	1.58e−4
35%_1.0 NRL-0.05TCNF-0.1GCA	1st_set	24 hours (1 day)	3.09e−5	0.259	0.517	3.63	11.9	1.59e−4
35%_1.0 NRL-0.05TCNF-0.1GCA	1st_set	7 days	3.01e−5	0.252	0.486	3.63	11.9	1.59e−4
35%_1.0 NRL-0.05TCNF-0.1GCA	1st_set	11 days	2.96e−5	0.248	0.468	3.63	11.9	1.58e−4
40%_1.0 NRL-0.05TCNF-0.1GCA	1st_set	1 hour	3.11e−5	0.261	0.532	3.63	11.9	1.58e−4
40%_1.0 NRL-0.05TCNF-0.1GCA	1st_set	24 hours (1 day)	3.00e−5	0.251	0.482	3.63	11.8	1.59e−4
40%_1.0 NRL-0.05TCN	1st_set	4 days	3.06e−5	0.256	0.481	3.63	11.9	1.58e−4

Formulation	Set	Age	First retained rate (1/s)	Strain in that point (%)	Cumulative strain (%)	Strain at 1e−3 1/s (%)	Cumulative at 1e−3 1/s (%)	Rate at 2% cumulative (1/s)
F-0.1GCA								
40%_1.0 NRL-0.05TCNF-0.1GCA	1st_set	7 days	3.06e−5	0.256	0.504	3.63	11.9	1.59e−4
40%_1.0 NRL-0.05TCNF-0.1GCA	1st_set	9 days	3.03e−5	0.254	0.489	3.63	11.9	1.58e−4
40%_1.0 NRL-0.05TCNF-0.1GCA	1st_set	14 days	2.91e−5	0.244	0.453	3.63	11.8	1.58e−4
25%_1.0 NRL-0.03TCNF-0.12GCA	2nd_set	1 hour	3.63e−5	0.304	0.749	3.59	11.9	1.52e−4
25%_1.0 NRL-0.03TCNF-0.12GCA	2nd_set	24 hours (1 day)	3.56e−5	0.298	0.732	3.58	11.8	1.52e−4
25%_1.0 NRL-0.03TCNF-0.12GCA	2nd_set	3 days	3.52e−5	0.295	0.724	3.63	11.9	1.51e−4
25%_1.0 NRL-0.03TCNF-0.12GCA	2nd_set	6 days	3.48e−5	0.292	0.696	3.63	11.9	1.51e−4
25%_1.0 NRL-0.03TCNF-0.12GCA	2nd_set	13 days	3.47e−5	0.291	0.694	3.63	11.9	1.51e−4
25%_1.0 NRL-0.05TCNF-0.1GCA	2nd_set	1 hour	3.39e−5	0.284	0.668	3.63	11.9	1.50e−4

Formulation	Set	Age	First retained rate (1/s)	Strain in that point (%)	Cumulative strain (%)	Strain at 1e−3 1/s (%)	Cumulative at 1e−3 1/s (%)	Rate at 2% cumulative (1/s)
25%_1.0 NRL-0.05TCNF-0.1GCA	2nd_set	24 hours (1 day)	3.38e−5	0.283	0.64	3.63	11.9	1.50e−4
25%_1.0 NRL-0.05TCNF-0.1GCA	2nd_set	7 days	3.23e−5	0.271	0.635	3.63	11.9	1.49e−4
25%_1.0 NRL-0.05TCNF-0.1GCA	2nd_set	11 days	3.21e−5	0.269	0.615	3.63	11.8	1.49e−4
25%_1.0 NRL-0.07TCNF-0.08GCA	2nd_set	1 hour	3.17e−5	0.266	0.572	3.63	11.8	1.59e−4
25%_1.0 NRL-0.07TCNF-0.08GCA	2nd_set	24 hours (1 day)	3.15e−5	0.264	0.554	3.63	11.8	1.59e−4
25%_1.0 NRL-0.07TCNF-0.08GCA	2nd_set	3 days	3.00e−5	0.251	0.583	3.63	11.8	1.58e−4
25%_1.0 NRL-0.07TCNF-0.08GCA	2nd_set	7 days	3.12e−5	0.261	0.534	3.63	11.9	1.59e−4
25%_1.0 NRL-0.07TCNF-0.08GCA	2nd_set	14 days	3.06e−5	0.256	0.524	3.63	11.8	1.58e−4
25%_1.0 NRL-0.10TCNF-0.05GCA	2nd_set	1 hour	3.04e−5	0.255	0.497	3.63	11.8	1.58e−4
25%_1.0 NRL-0.10TCN	2nd_set	24 hours (1 day)	2.99e−5	0.251	0.497	3.63	11.8	1.58e−4

Formulation	Set	Age	First retained rate (1/s)	Strain in that point (%)	Cumulative strain (%)	Strain at 1e−3 1/s (%)	Cumulative at 1e−3 1/s (%)	Rate at 2% cumulative (1/s)
F-0.05GCA								
25%_1.0 NRL-0.10TCN F-0.05GCA	2nd_set	3 days	2.96e−5	0.248	0.535	3.63	11.8	1.58e−4
25%_1.0 NRL-0.10TCN F-0.05GCA	2nd_set	6 days	2.91e−5	0.244	0.504	3.63	11.8	1.58e−4
25%_1.0 NRL-0.10TCN F-0.05GCA	2nd_set	13 days	2.91e−5	0.244	0.464	3.63	11.9	1.59e−4
25%_1.0 NRL-0.12TCN F-0.03GCA	2nd_set	1 hour	3.07e−5	0.257	0.542	3.63	11.9	1.59e−4
25%_1.0 NRL-0.12TCN F-0.03GCA	2nd_set	24 hours (1 day)	3.05e−5	0.256	0.522	3.63	11.9	1.58e−4
25%_1.0 NRL-0.12TCN F-0.03GCA	2nd_set	3 days	2.89e−5	0.242	0.455	3.63	11.8	1.58e−4
25%_1.0 NRL-0.12TCN F-0.03GCA	2nd_set	7 days	3.04e−5	0.255	0.478	3.63	11.9	1.58e−4
25%_1.0 NRL-0.12TCN F-0.03GCA	2nd_set	14 days	2.62e−5	0.22	0.373	3.63	11.8	1.59e−4
25%_1.0 NRL-0.15TCN F-0.0GCA	2nd_set	1 hour	2.83e−5	0.237	0.425	3.63	11.8	1.59e−4

Formulation	Set	Age	First retained rate (1/s)	Strain in that point (%)	Cumulative strain (%)	Strain at 1e−3 1/s (%)	Cumulative at 1e−3 1/s (%)	Rate at 2% cumulative (1/s)
25%_1.0 NRL-0.15TCNF-0.0GCA	2nd_set	24 hours (1 day)	2.72e−5	0.228	0.418	3.63	11.8	1.59e−4
25%_1.0 NRL-0.15TCNF-0.0GCA	2nd_set	4 days	2.81e−5	0.235	0.428	3.63	11.8	1.59e−4
25%_1.0 NRL-0.15TCNF-0.0GCA	2nd_set	7 days	2.73e−5	0.229	0.433	3.63	11.8	1.58e−4
25%_1.0 NRL-0.15TCNF-0.0GCA	2nd_set	14 days	2.62e−5	0.22	0.384	3.63	11.8	1.58e−4
25%_1.0 NRL-0.07TCNF-0.08GCA	3rd_set	1 hour	3.17e−5	0.266	0.572	3.63	11.8	1.59e−4
25%_1.0 NRL-0.07TCNF-0.08GCA	3rd_set	24 hours (1 day)	3.15e−5	0.264	0.554	3.63	11.8	1.59e−4
25%_1.0 NRL-0.07TCNF-0.08GCA	3rd_set	3 days	3.00e−5	0.251	0.583	3.63	11.8	1.58e−4
25%_1.0 NRL-0.07TCNF-0.08GCA	3rd_set	7 days	3.12e−5	0.261	0.534	3.63	11.9	1.59e−4
25%_1.0 NRL-0.07TCNF-0.08GCA	3rd_set	14 days	3.06e−5	0.256	0.524	3.63	11.8	1.58e−4
25%_1.0 NRL-0.07TCN	3rd_set	1 hour	3.16e−5	0.265	0.604	3.63	11.9	1.59e−4

Formulation	Set	Age	First retained rate (1/s)	Strain in that point (%)	Cumulative strain (%)	Strain at 1e−3 1/s (%)	Cumulative at 1e−3 1/s (%)	Rate at 2% cumulative (1/s)
F-0.0GCA								
25%_1.0 NRL-0.07TCNF-0.0GCA	3rd_set	24 hours (1 day)	3.09e−5	0.259	0.568	3.63	11.8	1.59e−4
25%_1.0 NRL-0.07TCNF-0.0GCA	3rd_set	3 days	3.00e−5	0.251	0.568	3.63	11.8	1.59e−4
25%_1.0 NRL-0.07TCNF-0.0GCA	3rd_set	7 days	3.16e−5	0.265	0.567	3.63	11.9	1.59e−4
25%_1.0 NRL-0.07TCNF-0.0GCA	3rd_set	29 days	3.07e−5	0.257	0.502	3.63	11.8	1.59e−4
25%_1.0 NRL-0.07TCNF-0.10GCA	3rd_set	1 hour	3.27e−5	0.274	0.599	3.63	11.9	1.59e−4
25%_1.0 NRL-0.07TCNF-0.10GCA	3rd_set	24 hours	3.25e−5	0.272	0.563	3.62	11.8	1.58e−4
25%_1.0 NRL-0.07TCNF-0.10GCA	3rd_set	3 days	2.89e−5	0.242	0.579	3.63	11.8	1.58e−4
25%_1.0 NRL-0.07TCNF-0.10GCA	3rd_set	7 days	2.91e−5	0.244	0.558	3.63	11.8	1.59e−4
25%_1.0 NRL-0.07TCNF-0.10GCA	3rd_set	29 days	3.04e−5	0.255	0.555	3.63	11.9	1.59e−4

Formulation	Set	Age	First retained rate (1/s)	Strain in that point (%)	Cumulative strain (%)	Strain at 1e−3 1/s (%)	Cumulative at 1e−3 1/s (%)	Rate at 2% cumulative (1/s)
25%_1.0 NRL-0.07TCNF-0.15GCA	3rd_set	1 hour	3.24e−5	0.272	0.588	3.63	11.9	1.59e−4
25%_1.0 NRL-0.07TCNF-0.15GCA	3rd_set	24 hours (1 day)	3.27e−5	0.274	0.576	3.63	11.9	1.59e−4
25%_1.0 NRL-0.07TCNF-0.15GCA	3rd_set	3 days	3.10e−5	0.26	0.564	3.63	11.8	1.59e−4
25%_1.0 NRL-0.07TCNF-0.15GCA	3rd_set	6 days	3.13e−5	0.262	0.579	3.63	11.9	1.58e−4
25%_1.0 NRL-0.07TCNF-0.15GCA	3rd_set	35 days	3.00e−5	0.251	0.53	3.63	11.9	1.59e−4
25%_1.0 NRL-0.07TCNF-0.20GCA	3rd_set	1 hour	3.25e−5	0.272	0.604	3.63	11.9	1.58e−4
25%_1.0 NRL-0.07TCNF-0.20GCA	3rd_set	24 hours (1 day)	3.28e−5	0.275	0.599	3.63	11.9	1.58e−4
25%_1.0 NRL-0.07TCNF-0.20GCA	3rd_set	3 days	3.10e−5	0.26	0.593	3.63	11.9	1.58e−4
25%_1.0 NRL-0.07TCNF-0.20GCA	3rd_set	6 days	2.99e−5	0.251	0.559	3.63	11.8	1.58e−4
25%_1.0 NRL-0.07TCN	3rd_set	35 days	3.06e−5	0.256	0.536	3.63	11.9	1.59e−4

Formulation	Set	Age	First retained rate (1/s)	Strain in that point (%)	Cumulative strain (%)	Strain at 1e−3 1/s (%)	Cumulative at 1e−3 1/s (%)	Rate at 2% cumulative (1/s)
F-0.20GCA								
25%_1.0 NRL-0.07TCN F-0.25GCA	3rd_set	1 hour	3.26e−5	0.273	0.61	3.63	11.8	1.51e−4
25%_1.0 NRL-0.07TCN F-0.25GCA	3rd_set	24 hours (1 day)	3.19e−5	0.267	0.595	3.63	11.8	1.55e−4
25%_1.0 NRL-0.07TCN F-0.25GCA	3rd_set	3 days	3.19e−5	0.267	0.593	3.63	11.9	1.58e−4
25%_1.0 NRL-0.07TCN F-0.25GCA	3rd_set	6 days	3.20e−5	0.268	0.581	3.63	11.8	1.58e−4
25%_1.0 NRL-0.07TCN F-0.25GCA	3rd_set	35 days	3.10e−5	0.26	0.537	3.63	11.9	1.59e−4
25%_1.0 NRL-0.07TCN F-0.15GCA	4th_set	1 hour	3.24e−5	0.272	0.588	3.63	11.9	1.59e−4
25%_1.0 NRL-0.07TCN F-0.15GCA	4th_set	24 hours (1 day)	3.27e−5	0.274	0.576	3.63	11.9	1.59e−4
25%_1.0 NRL-0.07TCN F-0.15GCA	4th_set	3 days	3.10e−5	0.26	0.564	3.63	11.8	1.59e−4
25%_1.0 NRL-0.07TCN F-0.15GCA	4th_set	6 days	3.13e−5	0.262	0.579	3.63	11.9	1.58e−4

Formulation	Set	Age	First retained rate (1/s)	Strain in that point (%)	Cumulative strain (%)	Strain at 1e−3 1/s (%)	Cumulative at 1e−3 1/s (%)	Rate at 2% cumulative (1/s)
25%_1.0 NRL-0.07TCNF-0.15GCA	4th_set	35 days	3.00e−5	0.251	0.53	3.63	11.9	1.59e−4
25%_1.0 NRL-0.07TCNF-0.15GNP	4th_set	1 hour	3.05e−5	0.256	0.503	3.63	11.9	1.59e−4
25%_1.0 NRL-0.07TCNF-0.15GNP	4th_set	24 hours (1 day)	3.32e−5	0.278	0.482	3.63	11.9	1.58e−4
25%_1.0 NRL-0.07TCNF-0.15GNP	4th_set	3 days	2.89e−5	0.242	0.443	3.63	11.8	1.59e−4
25%_1.0 NRL-0.07TCNF-0.15GNP	4th_set	6 days	2.86e−5	0.24	0.441	3.63	11.8	1.58e−4
25%_1.0 NRL-0.07TCNF-0.15GNP	4th_set	10 days	3.20e−5	0.268	0.485	3.63	11.9	1.59e−4
25%_1.0 NRL-0.07TCNF-0.15GNP	4th_set	15 days	2.92e−5	0.245	0.374	3.63	11.8	1.58e−4
25%_1.0 NRL-0.07TCNF-0.15Grpht	4th_set	1 hour	3.25e−5	0.272	0.601	3.63	11.9	1.56e−4
25%_1.0 NRL-0.07TCNF-0.15Grpht	4th_set	24 hours (1 day)	3.26e−5	0.273	0.591	3.63	11.9	1.59e−4
25%_1.0 NRL-0.07TCN	4th_set	4 days	3.16e−5	0.265	0.565	3.63	11.8	1.59e−4

Formulation	Set	Age	First retained rate (1/s)	Strain in that point (%)	Cumulative strain (%)	Strain at 1e−3 1/s (%)	Cumulative at 1e−3 1/s (%)	Rate at 2% cumulative (1/s)
F-0.15Grpht								
25%_1.0 NRL-0.07TCNF-0.15Grpht	4th_set	8 days	3.26e−5	0.273	0.561	3.63	11.9	1.58e−4
25%_1.0 NRL-0.07TCNF-0.15Grpht	4th_set	14 days	3.05e−5	0.256	0.561	3.63	11.8	1.59e−4
25%_1.0 NRL-0.07TCNF-0.15MWNT-CO	4th_set	1 hour	3.00e−5	0.251	0.487	3.63	11.9	1.59e−4
25%_1.0 NRL-0.07TCNF-0.15MWNT-CO	4th_set	24 hours (1 day)	2.92e−5	0.245	0.487	3.63	11.9	1.59e−4
25%_1.0 NRL-0.07TCNF-0.15MWNT-CO	4th_set	4 days	2.85e−5	0.239	0.399	3.62	11.8	1.59e−4
25%_1.0 NRL-0.07TCNF-0.15MWNT-CO	4th_set	8 days	3.47e−5	0.291	0.498	3.63	11.9	1.59e−4
25%_1.0 NRL-0.07TCNF-0.15MWNT-CO	4th_set	13 days	2.77e−5	0.232	0.425	3.63	11.8	1.59e−4
25%_1.0 NRL-0.07TCNF-0.15MWNT	4th_set	1 hour	3.03e−5	0.254	0.49	3.63	11.9	1.59e−4

Formulation	Set	Age	First retained rate (1/s)	Strain in that point (%)	Cumulative strain (%)	Strain at 1e−3 1/s (%)	Cumulative at 1e−3 1/s (%)	Rate at 2% cumulative (1/s)
25%_1.0NRL-0.07TCNF-0.15MWNT	4th_set	24 hours (1 day)	3.00e−5	0.251	0.441	3.63	11.8	1.58e−4
25%_1.0NRL-0.07TCNF-0.15MWNT	4th_set	3 days	3.05e−5	0.256	0.494	3.63	11.9	1.58e−4
25%_1.0NRL-0.07TCNF-0.15MWNT	4th_set	6 days	2.82e−5	0.236	0.427	3.63	11.8	1.59e−4
25%_1.0NRL-0.07TCNF-0.15MWNT	4th_set	10 days	3.25e−5	0.272	0.478	3.63	11.9	1.58e−4
25%_1.0NRL-0.07TCNF-0.15MWNT	4th_set	15 days	3.98e−5	0.334	0.601	3.63	12	1.59e−4

Figure S2. Strain amplitude sweeps.

a) Storage modulus against strain amplitude. b) The same data normalized to the plateau modulus of each sweep, with the 5% departure criterion marked. Open, faded symbols are points whose torque is below the 0.1 μN·m minimum of the instrument and are shown for completeness only. The dotted vertical line is the 0.1% working strain used in all frequency sweeps, which lies at least seven times below the narrowest limit measured.

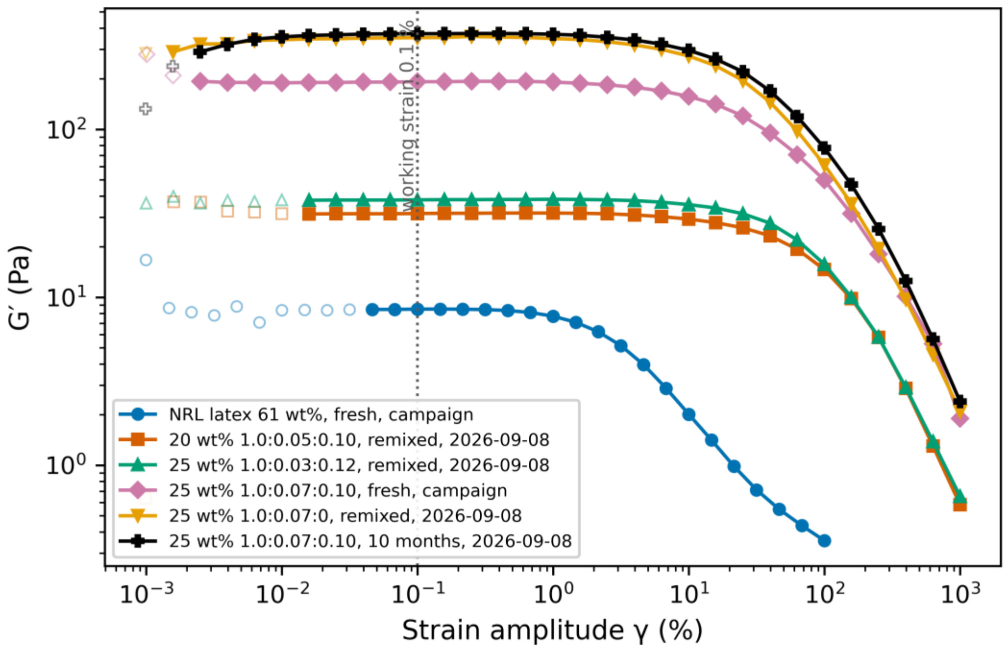

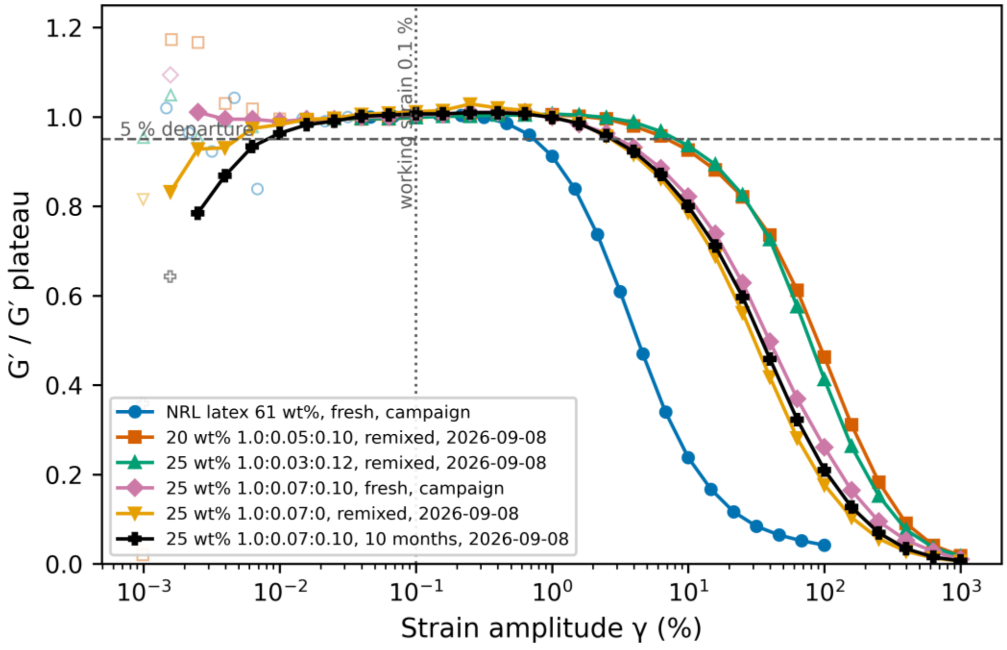

Table S8. Overlapping range of the Cox–Merz comparison.

The oscillatory and the steady-shear measurement of a given formulation and storage time were made on the same loading, one after the other, so the comparison is between two protocols applied

to one specimen and not between two separately loaded specimens. The overlapping range is the intersection of the retained shear-rate window with the angular-frequency window, and the point counts are measured points inside it, not interpolated ones.

Set	Formulation	Lower limit (1/s)	Upper limit (1/s)	Decades	Flow points	Oscillatory points
1st_set	20%_1.0NRL -0.05TCNF-0.1GCA	0.0628	39.6	2.8	14	15
1st_set	25%_1.0NRL -0.05TCNF-0.1GCA	0.0628	39.6	2.8	14	15
1st_set	30%_1.0NRL -0.05TCNF-0.1GCA	0.0628	39.6	2.8	14	15
1st_set	35%_1.0NRL -0.05TCNF-0.1GCA	0.0628	39.6	2.8	14	15
1st_set	40%_1.0NRL -0.05TCNF-0.1GCA	0.0628	39.6	2.8	14	15
2nd_set	25%_1.0NRL -0.03TCNF-0.12GCA	0.0628	39.6	2.8	14	15
2nd_set	25%_1.0NRL -0.05TCNF-0.1GCA	0.0628	39.6	2.8	14	15
2nd_set	25%_1.0NRL -0.07TCNF-0.08GCA	0.0628	39.6	2.8	14	15
2nd_set	25%_1.0NRL -0.10TCNF-0.05GCA	0.0628	39.6	2.8	14	15
2nd_set	25%_1.0NRL -0.12TCNF-0.03GCA	0.0628	39.6	2.8	14	15
2nd_set	25%_1.0NRL -0.15TCNF-0.0GCA	0.0628	39.6	2.8	14	15
3rd_set	25%_1.0NRL -0.07TCNF-0.08GCA	0.0628	39.6	2.8	14	15
3rd_set	25%_1.0NRL -0.07TCNF-0.0GCA	0.0628	39.6	2.8	14	15

Set	Formulation	Lower limit (1/s)	Upper limit (1/s)	Decades	Flow points	Oscillatory points
3rd_set	25%_1.0NRL-0.07TCNF-0.15GCA	0.0628	39.6	2.8	14	15
3rd_set	25%_1.0NRL-0.07TCNF-0.1GCA	0.0628	39.6	2.8	14	15
3rd_set	25%_1.0NRL-0.07TCNF-0.25GCA	0.0628	39.6	2.8	14	15
3rd_set	25%_1.0NRL-0.07TCNF-0.2GCA	0.0628	39.6	2.8	14	15
4th_set	25%_1.0NRL-0.07TCNF-0.15GCA	0.0628	39.6	2.8	14	15
4th_set	25%_1.0NRL-0.07TCNF-0.15GNP	0.0628	39.6	2.8	14	15
4th_set	25%_1.0NRL-0.07TCNF-0.15MWCNT	0.0628	39.6	2.8	14	15
4th_set	25%_1.0NRL-0.07TCNF-0.15MWCNT-COOH	0.0628	39.6	2.8	14	15
4th_set	25%_1.0NRL-0.07TCNF-0.15graphite	0.0628	39.6	2.8	14	15

Table S9. Rank agreement between the oscillatory and steady-shear orderings.

Spearman coefficient between the ranking by G′(1 Hz) and the ranking by the low-shear Cross parameter, with a two-sided p value obtained by enumerating every permutation of the labels rather than from an asymptotic approximation. With five or six formulations, the smallest attainable p is 1/60 or 1/360, so the p value is bounded from below by the design and is reported as a descriptive statement about these measured rankings, not as evidence of a mechanism or of reproducibility. Ties were resolved with average ranks.

Set	Formulations	Spearman ρ	Exact two-sided p	Permutations enumerated	Smallest attainable p	Tied values
1st_set	5	1	0.0167	120	0.0167	0

Set	Formulations	Spearman ρ	Exact two-sided p	Permutations enumerated	Smallest attainable p	Tied values
2nd_set	6	1	2.78e−3	720	2.78e−3	0
3rd_set	6	0.886	0.0333	720	2.78e−3	0
4th_set	5	0.975	0.0333	120	0.0167	1

Table S10. Regression statistics for the aging trend.

Two descriptions are fitted to each formulation over its measured storage window: a straight line against age, and a power law, that is, a straight line in logarithmic coordinates. The residual column is the largest absolute residual as a fraction of the total range of the fitted quantity. For the low-shear Cross parameter, which is the quantity the Conclusions describe as approximately linear in time, the linear description has a median R2 of 0.900 and is the better of the two in 15 of 22 formulations, and in four of the five formulations of Set 1. For the storage modulus the power law is clearly the better description, with a median R2 of 0.989 against 0.803. Neither description is used outside the measured window.

Set	Formulation	Quantity	Model	Ages	Slope	Slope standard error	R2	Largest residual/range
1st_set	20%_1.0 NRL-0.05TCNF-0.1GCA	Cross low-shear parameter	linear_in_age	4	338	58.9	0.943	0.124
1st_set	20%_1.0 NRL-0.05TCNF-0.1GCA	Cross low-shear parameter	log_log	4	0.101	0.0228	0.908	0.148
1st_set	25%_1.0 NRL-0.05TCNF-0.1GCA	Cross low-shear parameter	linear_in_age	4	943	195	0.921	0.147
1st_set	25%_1.0 NRL-0.05TCNF-0.1GCA	Cross low-shear parameter	log_log	4	0.104	0.0118	0.975	0.082

Set	Formulation	Quantity	Model	Ages	Slope	Slope standard error	R2	Largest residual/range
1st_set	30%_1.0 NRL-0.05TCNF-0.1GCA	Cross low-shear parameter	linear_in_age	5	1.63e+03	334	0.888	0.206
1st_set	30%_1.0 NRL-0.05TCNF-0.1GCA	Cross low-shear parameter	log_log	5	0.0842	0.0244	0.799	0.222
1st_set	35%_1.0 NRL-0.05TCNF-0.1GCA	Cross low-shear parameter	linear_in_age	4	2.39e+03	475	0.926	0.186
1st_set	35%_1.0 NRL-0.05TCNF-0.1GCA	Cross low-shear parameter	log_log	4	0.0551	0.0146	0.878	0.263
1st_set	40%_1.0 NRL-0.05TCNF-0.1GCA	Cross low-shear parameter	linear_in_age	6	2.18e+03	340	0.911	0.239
1st_set	40%_1.0 NRL-0.05TCNF-0.1GCA	Cross low-shear parameter	log_log	6	0.0542	0.0165	0.728	0.27
2nd_set	25%_1.0 NRL-0.03TCNF-0.12GCA	Cross low-shear parameter	linear_in_age	5	411	118	0.801	0.283
2nd_set	25%_1.0 NRL-0.03TCNF-0.12GCA	Cross low-shear parameter	log_log	5	0.155	0.0279	0.911	0.176
2nd_set	25%_1.0 NRL-0.05TCNF-0.1GCA	Cross low-shear parameter	linear_in_age	4	943	195	0.921	0.147
2nd_set	25%_1.0 NRL-0.05TCN	Cross low-shear parameter	log_log	4	0.104	0.0118	0.975	0.082

Set	Formulation	Quantity	Model	Ages	Slope	Slope standard error	R2	Largest residual/range
	F-0.1GCA							
2nd_set	25%_1.0NRL-0.07TCNF-0.08GCA	Cross low-shear parameter	linear_in_age	5	1.46e+03	282	0.9	0.225
2nd_set	25%_1.0NRL-0.07TCNF-0.08GCA	Cross low-shear parameter	log_log	5	0.0793	0.0225	0.806	0.212
2nd_set	25%_1.0NRL-0.10TCNF-0.05GCA	Cross low-shear parameter	linear_in_age	5	2.78e+03	547	0.896	0.192
2nd_set	25%_1.0NRL-0.10TCNF-0.05GCA	Cross low-shear parameter	log_log	5	0.0465	0.0331	0.397	0.489
2nd_set	25%_1.0NRL-0.12TCNF-0.03GCA	Cross low-shear parameter	linear_in_age	5	3.93e+03	598	0.935	0.139
2nd_set	25%_1.0NRL-0.12TCNF-0.03GCA	Cross low-shear parameter	log_log	5	0.0862	0.0222	0.835	0.245
2nd_set	25%_1.0NRL-0.15TCNF-0.0GCA	Cross low-shear parameter	linear_in_age	5	4.46e+03	740	0.924	0.154
2nd_set	25%_1.0NRL-0.15TCNF-0.0GCA	Cross low-shear parameter	log_log	5	0.0646	0.0151	0.859	0.225
3rd_set	25%_1.0NRL-0.07TCNF-0.08GCA	Cross low-shear parameter	linear_in_age	5	1.46e+03	282	0.9	0.225

Set	Formulation	Quantity	Model	Ages	Slope	Slope standard error	R2	Largest residual/range
3rd_set	25%_1.0 NRL-0.07TCNF-0.08GCA	Cross low-shear parameter	log_log	5	0.0793	0.0225	0.806	0.212
3rd_set	25%_1.0 NRL-0.07TCNF-0.0GCA	Cross low-shear parameter	linear_in_age	5	953	288	0.784	0.273
3rd_set	25%_1.0 NRL-0.07TCNF-0.0GCA	Cross low-shear parameter	log_log	5	0.102	0.0165	0.926	0.131
3rd_set	25%_1.0 NRL-0.07TCNF-0.1GCA	Cross low-shear parameter	linear_in_age	5	431	190	0.631	0.356
3rd_set	25%_1.0 NRL-0.07TCNF-0.1GCA	Cross low-shear parameter	log_log	5	0.0679	1.14e−3	0.999	0.0191
3rd_set	25%_1.0 NRL-0.07TCNF-0.15GCA	Cross low-shear parameter	linear_in_age	5	633	106	0.923	0.144
3rd_set	25%_1.0 NRL-0.07TCNF-0.15GCA	Cross low-shear parameter	log_log	5	0.0796	0.0197	0.845	0.187
3rd_set	25%_1.0 NRL-0.07TCNF-0.2GCA	Cross low-shear parameter	linear_in_age	5	685	117	0.919	0.188
3rd_set	25%_1.0 NRL-0.07TCNF-0.2GCA	Cross low-shear parameter	log_log	5	0.0831	0.025	0.787	0.209
3rd_set	25%_1.0 NRL-0.07TCN	Cross low-shear parameter	linear_in_age	5	630	138	0.874	0.185

Set	Formulation	Quantity	Model	Ages	Slope	Slope standard error	R2	Largest residual/range
	F-0.25GCA							
3rd_set	25%_1.0NRL-0.07TCNF-0.25GCA	Cross low-shear parameter	log_log	5	0.0966	0.024	0.844	0.219
4th_set	25%_1.0NRL-0.07TCNF-0.15GCA	Cross low-shear parameter	linear_in_age	5	633	106	0.923	0.144
4th_set	25%_1.0NRL-0.07TCNF-0.15GCA	Cross low-shear parameter	log_log	5	0.0796	0.0197	0.845	0.187
4th_set	25%_1.0NRL-0.07TCNF-0.15GNP	Cross low-shear parameter	linear_in_age	6	3.64e+03	1.15e+03	0.715	0.324
4th_set	25%_1.0NRL-0.07TCNF-0.15GNP	Cross low-shear parameter	log_log	6	0.0864	3.11e−3	0.995	0.0379
4th_set	25%_1.0NRL-0.07TCNF-0.15graphite	Cross low-shear parameter	linear_in_age	5	1.28e+03	381	0.791	0.263
4th_set	25%_1.0NRL-0.07TCNF-0.15graphite	Cross low-shear parameter	log_log	5	0.0894	0.0171	0.901	0.202
4th_set	25%_1.0NRL-0.07TCNF-0.15MWCNT-COOH	Cross low-shear parameter	linear_in_age	5	3.11e+03	719	0.861	0.216
4th_set	25%_1.0NRL-0.07TCNF-	Cross low-shear parameter	log_log	5	0.0415	0.0208	0.569	0.467

Set	Formulation	Quantity	Model	Ages	Slope	Slope standard error	R2	Largest residual/range
	0.15MWCNT-COOH							
4th_set	25%_1.0NRL-0.07TCNF-0.15MWCNT	Cross low-shear parameter	linear_in_age	6	1.37e4	3.22e+03	0.818	0.268
4th_set	25%_1.0NRL-0.07TCNF-0.15MWCNT	Cross low-shear parameter	log_log	6	0.141	0.0692	0.51	0.451
1st_set	20%_1.0NRL-0.05TCNF-0.1GCA	G′(1 Hz)	linear_in_age	4	5.43	1.54	0.862	0.195
1st_set	20%_1.0NRL-0.05TCNF-0.1GCA	G′(1 Hz)	log_log	4	0.192	0.0163	0.986	0.0705
1st_set	25%_1.0NRL-0.05TCNF-0.1GCA	G′(1 Hz)	linear_in_age	4	12.7	3.75	0.852	0.201
1st_set	25%_1.0NRL-0.05TCNF-0.1GCA	G′(1 Hz)	log_log	4	0.17	6.08e−3	0.997	0.0285
1st_set	30%_1.0NRL-0.05TCNF-0.1GCA	G′(1 Hz)	linear_in_age	5	18.1	5.59	0.778	0.264
1st_set	30%_1.0NRL-0.05TCNF-0.1GCA	G′(1 Hz)	log_log	5	0.139	4.57e−3	0.997	0.0325
1st_set	35%_1.0NRL-0.05TCNF-0.1GCA	G′(1 Hz)	linear_in_age	4	32.1	9.75	0.844	0.196

Set	Formulation	Quantity	Model	Ages	Slope	Slope standard error	R2	Largest residual/range
1st_set	35%_1.0 NRL-0.05TCNF-0.1GCA	G′(1 Hz)	log_log	4	0.0977	8.09e−3	0.986	0.0748
1st_set	40%_1.0 NRL-0.05TCNF-0.1GCA	G′(1 Hz)	linear_in_age	6	26.3	4.11	0.911	0.147
1st_set	40%_1.0 NRL-0.05TCNF-0.1GCA	G′(1 Hz)	log_log	6	0.0736	0.0148	0.86	0.236
2nd_set	25%_1.0 NRL-0.03TCNF-0.12GCA	G′(1 Hz)	linear_in_age	5	4.67	1.31	0.808	0.23
2nd_set	25%_1.0 NRL-0.03TCNF-0.12GCA	G′(1 Hz)	log_log	5	0.19	0.0147	0.982	0.067
2nd_set	25%_1.0 NRL-0.05TCNF-0.1GCA	G′(1 Hz)	linear_in_age	4	12.7	3.75	0.852	0.201
2nd_set	25%_1.0 NRL-0.05TCNF-0.1GCA	G′(1 Hz)	log_log	4	0.17	6.08e−3	0.997	0.0285
2nd_set	25%_1.0 NRL-0.07TCNF-0.08GCA	G′(1 Hz)	linear_in_age	5	15	4.8	0.764	0.27
2nd_set	25%_1.0 NRL-0.07TCNF-0.08GCA	G′(1 Hz)	log_log	5	0.112	6.88e−3	0.989	0.0636
2nd_set	25%_1.0 NRL-0.10TCN	G′(1 Hz)	linear_in_age	5	32.8	6.27	0.901	0.163

Set	Formulation	Quantity	Model	Ages	Slope	Slope standard error	R2	Largest residual/range
	F-0.05GCA							
2nd_set	25%_1.0NRL-0.10TCNF-0.05GCA	G′(1 Hz)	log_log	5	0.0995	0.0192	0.9	0.17
2nd_set	25%_1.0NRL-0.12TCNF-0.03GCA	G′(1 Hz)	linear_in_age	5	42.3	7.91	0.905	0.171
2nd_set	25%_1.0NRL-0.12TCNF-0.03GCA	G′(1 Hz)	log_log	5	0.113	0.016	0.943	0.126
2nd_set	25%_1.0NRL-0.15TCNF-0.0GCA	G′(1 Hz)	linear_in_age	5	44.9	13	0.798	0.254
2nd_set	25%_1.0NRL-0.15TCNF-0.0GCA	G′(1 Hz)	log_log	5	0.0831	5.01e−3	0.989	0.0558
3rd_set	25%_1.0NRL-0.07TCNF-0.08GCA	G′(1 Hz)	linear_in_age	5	15	4.8	0.764	0.27
3rd_set	25%_1.0NRL-0.07TCNF-0.08GCA	G′(1 Hz)	log_log	5	0.112	6.88e−3	0.989	0.0636
3rd_set	25%_1.0NRL-0.07TCNF-0.0GCA	G′(1 Hz)	linear_in_age	5	8.13	3.99	0.58	0.382
3rd_set	25%_1.0NRL-0.07TCNF-0.0GCA	G′(1 Hz)	log_log	5	0.13	7.09e−3	0.991	0.0466
3rd_set	25%_1.0NRL-	G′(1 Hz)	linear_in_age	5	6.7	2.24	0.749	0.289

Set	Formulation	Quantity	Model	Ages	Slope	Slope standard error	R2	Largest residual/range
	0.07TCNF-0.15GCA							
3rd_set	25%_1.0NRL-0.07TCNF-0.15GCA	G′(1 Hz)	log_log	5	0.122	5.37e−3	0.994	0.0354
3rd_set	25%_1.0NRL-0.07TCNF-0.1GCA	G′(1 Hz)	linear_in_age	5	5.51	2.8	0.563	0.357
3rd_set	25%_1.0NRL-0.07TCNF-0.1GCA	G′(1 Hz)	log_log	5	0.0957	0.0116	0.958	0.128
3rd_set	25%_1.0NRL-0.07TCNF-0.2GCA	G′(1 Hz)	linear_in_age	5	6.63	2.77	0.656	0.323
3rd_set	25%_1.0NRL-0.07TCNF-0.2GCA	G′(1 Hz)	log_log	5	0.135	7.89e−3	0.99	0.0587
3rd_set	25%_1.0NRL-0.07TCNF-0.25GCA	G′(1 Hz)	linear_in_age	5	5.93	1.94	0.756	0.277
3rd_set	25%_1.0NRL-0.07TCNF-0.25GCA	G′(1 Hz)	log_log	5	0.121	7.28e−3	0.989	0.0447
4th_set	25%_1.0NRL-0.07TCNF-0.15GCA	G′(1 Hz)	linear_in_age	5	6.7	2.24	0.749	0.289
4th_set	25%_1.0NRL-0.07TCNF-0.15GCA	G′(1 Hz)	log_log	5	0.122	5.37e−3	0.994	0.0354

Set	Formulation	Quantity	Model	Ages	Slope	Slope standard error	R2	Largest residual/range
4th_set	25%_1.0NRL-0.07TCNF-0.15GNP	G′(1 Hz)	linear_in_age	6	47	9.3	0.865	0.197
4th_set	25%_1.0NRL-0.07TCNF-0.15GNP	G′(1 Hz)	log_log	6	0.13	0.0145	0.952	0.11
4th_set	25%_1.0NRL-0.07TCNF-0.15graphite	G′(1 Hz)	linear_in_age	5	15.8	5.08	0.764	0.269
4th_set	25%_1.0NRL-0.07TCNF-0.15graphite	G′(1 Hz)	log_log	5	0.13	7.33e−3	0.991	0.0575
4th_set	25%_1.0NRL-0.07TCNF-0.15MWCNT-COOH	G′(1 Hz)	linear_in_age	5	45.2	11.9	0.828	0.223
4th_set	25%_1.0NRL-0.07TCNF-0.15MWCNT-COOH	G′(1 Hz)	log_log	5	0.101	8.28e−3	0.98	0.0959
4th_set	25%_1.0NRL-0.07TCNF-0.15MWCNT	G′(1 Hz)	linear_in_age	6	101	11.9	0.948	0.127
4th_set	25%_1.0NRL-0.07TCNF-0.15MWCNT	G′(1 Hz)	log_log	6	0.152	0.0476	0.719	0.313

Table S11. Component masses per 100 g of hydrogel.

Dry masses of the three components, followed by the masses of the as-received materials that supply them and the deionized water added beyond what those already contain. The latex is a 61 wt% dispersion and the TCNF is used as a 6 wt% stock, so both bring water with them. Batch masses as weighed were not recorded, so the table is given per 100 g of hydrogel, which is what a reader needs to reproduce a formulation. Sodium azide is present at 0.02 wt% of the aqueous phase and is not counted in the total solids.

Set	Formulation	Additive	Total solids (wt%)	NRL dry (g)	TCNF dry (g)	Additive (g)	NRL dispersion 61 wt% (g)	TCNF stock 6 wt% (g)	Water added (g)	Azide (mg)
1st_set	20%_1.0NRL-0.05TCNF-0.1GCA	GCA	20	17.4	0.87	1.74	28.5	14.5	55.3	16
1st_set	25%_1.0NRL-0.05TCNF-0.1GCA	GCA	25	21.7	1.09	2.17	35.6	18.1	44.1	15
1st_set	30%_1.0NRL-0.05TCNF-0.1GCA	GCA	30	26.1	1.3	2.61	42.8	21.7	32.9	14
1st_set	35%_1.0NRL-0.05TCNF-0.1GCA	GCA	35	30.4	1.52	3.04	49.9	25.4	21.7	13
1st_set	40%_1.0NRL-0.05TCNF-0.1GCA	GCA	40	34.8	1.74	3.48	57	29	10.5	12

Set	Formulation	Additive	Total solids (wt%)	NRL dry (g)	TCNF dry (g)	Additive (g)	NRL dispersion 61 wt% (g)	TCNF stock 6 wt% (g)	Water added (g)	Azide (mg)
2nd_set	25%_1.0NRL-0.03TCNF-0.12GCA	GCA	25	21.7	0.652	2.61	35.6	10.9	50.9	15
2nd_set	25%_1.0NRL-0.05TCNF-0.1GCA	GCA	25	21.7	1.09	2.17	35.6	18.1	44.1	15
2nd_set	25%_1.0NRL-0.07TCNF-0.08GCA	GCA	25	21.7	1.52	1.74	35.6	25.4	37.3	15
2nd_set	25%_1.0NRL-0.10TCNF-0.05GCA	GCA	25	21.7	2.17	1.09	35.6	36.2	27	15
2nd_set	25%_1.0NRL-0.12TCNF-0.03GCA	GCA	25	21.7	2.61	0.652	35.6	43.5	20.2	15
2nd_set	25%_1.0NRL-0.15TCNF-0.0GCA	none	25	21.7	3.26	0	35.6	54.3	10	15
3rd_set	25%_1.0NRL-0.07TCNF-0.08GCA	GCA	25	21.7	1.52	1.74	35.6	25.4	37.3	15
3rd_set	25%_1.0NRL-0.07TCNF-0.0GCA	none	25	23.4	1.64	0	38.3	27.3	34.4	15

Set	Formulation	Additive	Total solids (wt%)	NRL dry (g)	TCNF dry (g)	Additive (g)	NRL dispersion 61 wt% (g)	TCNF stock 6 wt% (g)	Water added (g)	Azide (mg)
3rd_set	25%_1.0NRL-0.07TCNF-0.1GCA	GCA	25	21.4	1.5	2.14	35	24.9	37.9	15
3rd_set	25%_1.0NRL-0.07TCNF-0.15GCA	GCA	25	20.5	1.43	3.07	33.6	23.9	39.4	15
3rd_set	25%_1.0NRL-0.07TCNF-0.2GCA	GCA	25	19.7	1.38	3.94	32.3	23	40.8	15
3rd_set	25%_1.0NRL-0.07TCNF-0.25GCA	GCA	25	18.9	1.33	4.73	31	22.1	42.1	15
4th_set	25%_1.0NRL-0.07TCNF-0.15GCA	GCA	25	20.5	1.43	3.07	33.6	23.9	39.4	15
4th_set	25%_1.0NRL-0.07TCNF-0.15GNP	GNP	25	20.5	1.43	3.07	33.6	23.9	39.4	15
4th_set	25%_1.0NRL-0.07TCNF-0.15graphite	graphite	25	20.5	1.43	3.07	33.6	23.9	39.4	15
4th_set	25%_1.0NRL-0.07TCNF-0.15M	MWCNT-COOH	25	20.5	1.43	3.07	33.6	23.9	39.4	15

Set	Formulation	Additive	Total solids (wt%)	NRL dry (g)	TCNF dry (g)	Additive (g)	NRL dispersion 61 wt% (g)	TCNF stock 6 wt% (g)	Water added (g)	Azide (mg)
4th_set	WCNT-COOH 25%_1.0NRL-0.07TCNF-0.15MWCNT	MWCNT	25	20.5	1.43	3.07	33.6	23.9	39.4	15

Table S12. Bootstrap interval for the low-shear Cross parameter.

The intervals of Table S1 are linearized symmetric intervals computed from the Jacobian at the optimum, and the shear-thinning exponent sits at its upper bound in 97 of 109 fits, where that construction is not obviously reliable. Each curve was therefore refitted 400 times on its own residuals resampled with replacement, under the same constraint. The resulting interval is mildly asymmetric, with the upper arm about 1.3 times the lower, and its width is on average 0.89 times the width of the symmetric interval, so the reported intervals are slightly conservative rather than optimistic. Both describe uncertainty in the fit only, not experimental or batch variability.

Set	Formulation	Age	Low-shear parameter (Pa·s)	Symmetric ±(%)	Bootstrap lower arm (%)	Bootstrap upper arm (%)	Bootstrap width/symmetric width	Exponent at bound
1st_set	20%_1.0NRL-0.05TCNF-0.1GCA	1 hour	4.77e+03	12	11.3	11.7	0.962	0
1st_set	20%_1.0NRL-0.05TCNF-0.1GCA	24 hours (1 day)	6.08e+03	12.9	10.5	11.9	0.87	0
1st_set	20%_1.0NRL-0.05TCNF-0.1GCA	4 days	6.76e+03	13.5	11.8	14.5	0.973	0

Set	Formulation	Age	Low-shear parameter (Pa·s)	Symmetric ±(%)	Bootstrap lower arm (%)	Bootstrap upper arm (%)	Bootstrap width/symmetric width	Exponent at bound
1st_set	20%_1.0 NRL-0.05TCNF-0.1GCA	11 days	8.9e+03	13.4	9.82	14.9	0.919	1
1st_set	25%_1.0 NRL-0.05TCNF-0.1GCA	1 hour	1.38e4	11.6	11.8	9.61	0.924	0
1st_set	25%_1.0 NRL-0.05TCNF-0.1GCA	24 hours (1 day)	1.81e4	11.8	8.59	10.6	0.816	0
1st_set	25%_1.0 NRL-0.05TCNF-0.1GCA	7 days	2.24e4	14.7	12.4	13.2	0.87	1
1st_set	25%_1.0 NRL-0.05TCNF-0.1GCA	11 days	2.55e4	17	11.9	16.8	0.841	1
1st_set	30%_1.0 NRL-0.05TCNF-0.1GCA	1 hour	3.33e4	13.6	10.9	13	0.879	0
1st_set	30%_1.0 NRL-0.05TCNF-0.1GCA	24 hours (1 day)	3.75e4	8.23	6.66	7.28	0.848	1
1st_set	30%_1.0 NRL-0.05TCNF-0.1GCA	4 days	4.73e4	13.8	10.8	13.9	0.899	1
1st_set	30%_1.0 NRL-0.05TCNF-0.1GCA	7 days	4.40e4	11.6	9.32	10.6	0.856	1
1st_set	30%_1.0 NRL-0.05TCN	14 days	5.84e4	15.2	10.8	14.2	0.819	1

Set	Formulation	Age	Low-shear parameter (Pa·s)	Symmetric ±(%)	Bootstrap lower arm (%)	Bootstrap upper arm (%)	Bootstrap width/symmetric width	Exponent at bound
	F-0.1GCA							
1st_set	35%_1.0NRL-0.05TCNF-0.1GCA	1 hour	7.34e4	11.3	7.54	11.8	0.855	1
1st_set	35%_1.0NRL-0.05TCNF-0.1GCA	24 hours (1 day)	7.84e4	8.54	6.38	9.07	0.904	1
1st_set	35%_1.0NRL-0.05TCNF-0.1GCA	7 days	9.69e4	10.4	8	10.2	0.874	1
1st_set	35%_1.0NRL-0.05TCNF-0.1GCA	11 days	9.87e4	12.7	8.58	13.2	0.86	1
1st_set	40%_1.0NRL-0.05TCNF-0.1GCA	1 hour	7.32e4	7.09	4.75	8.02	0.9	1
1st_set	40%_1.0NRL-0.05TCNF-0.1GCA	24 hours (1 day)	7.67e4	6.59	4.64	7.24	0.901	1
1st_set	40%_1.0NRL-0.05TCNF-0.1GCA	4 days	8.17e4	10.4	8.64	10.2	0.906	1
1st_set	40%_1.0NRL-0.05TCNF-0.1GCA	7 days	8.99e4	6.96	5.26	7.92	0.947	1
1st_set	40%_1.0NRL-0.05TCNF-0.1GCA	9 days	1.01e5	7.6	5.76	8.55	0.941	1

Set	Formulation	Age	Low-shear parameter (Pa·s)	Symmetric ±(%)	Bootstrap lower arm (%)	Bootstrap upper arm (%)	Bootstrap width/symmetric width	Exponent at bound
1st_set	40%_1.0NRL-0.05TCNF-0.1GCA	14 days	1.01e5	7.93	6.44	7.74	0.894	1
2nd_set	25%_1.0NRL-0.03TCNF-0.12GCA	1 hour	4.08e+03	8.58	7.4	8.55	0.93	0
2nd_set	25%_1.0NRL-0.03TCNF-0.12GCA	24 hours (1 day)	5.34e+03	11.5	8.91	11.7	0.9	0
2nd_set	25%_1.0NRL-0.03TCNF-0.12GCA	3 days	6.86e+03	12.1	9.72	11.8	0.889	0
2nd_set	25%_1.0NRL-0.03TCNF-0.12GCA	6 days	9.16e+03	14.8	11.9	14.5	0.893	1
2nd_set	25%_1.0NRL-0.03TCNF-0.12GCA	13 days	9.64e+03	11.4	8.71	10.5	0.839	1
2nd_set	25%_1.0NRL-0.05TCNF-0.1GCA	1 hour	1.38e4	11.6	9.91	10.7	0.889	0
2nd_set	25%_1.0NRL-0.05TCNF-0.1GCA	24 hours (1 day)	1.81e4	11.8	11.6	10.8	0.954	0
2nd_set	25%_1.0NRL-0.05TCNF-0.1GCA	7 days	2.24e4	14.7	13.7	14.3	0.952	1
2nd_set	25%_1.0NRL-0.05TCN	11 days	2.55e4	17	14.4	16.7	0.913	1

Set	Formulation	Age	Low-shear parameter (Pa·s)	Symmetric ±(%)	Bootstrap lower arm (%)	Bootstrap upper arm (%)	Bootstrap width/symmetric width	Exponent at bound
	F-0.1GCA							
2nd_set	25%_1.0 NRL-0.07TCNF-0.08GCA	1 hour	3.39e4	12.1	11.4	11.7	0.952	1
2nd_set	25%_1.0 NRL-0.07TCNF-0.08GCA	24 hours (1 day)	3.70e4	10.4	7.75	11.3	0.916	1
2nd_set	25%_1.0 NRL-0.07TCNF-0.08GCA	3 days	4.06e4	16.5	13.4	15.2	0.869	1
2nd_set	25%_1.0 NRL-0.07TCNF-0.08GCA	7 days	5.07e4	18.7	13.3	18	0.839	1
2nd_set	25%_1.0 NRL-0.07TCNF-0.08GCA	14 days	5.41e4	20.1	16.4	18.1	0.856	1
2nd_set	25%_1.0 NRL-0.10TCNF-0.05GCA	1 hour	7.50e4	14.9	10.8	13.3	0.806	1
2nd_set	25%_1.0 NRL-0.10TCNF-0.05GCA	24 hours (1 day)	7.72e4	12.1	8.39	12.4	0.857	1
2nd_set	25%_1.0 NRL-0.10TCNF-0.05GCA	3 days	7.61e4	11.4	8.48	10.8	0.843	1
2nd_set	25%_1.0 NRL-0.10TCNF-0.05GCA	6 days	8.10e4	11.7	8.32	10.9	0.824	1

Set	Formulation	Age	Low-shear parameter (Pa·s)	Symmetric ±(%)	Bootstrap lower arm (%)	Bootstrap upper arm (%)	Bootstrap width/symmetric width	Exponent at bound
2nd_set	25%_1.0NRL-0.10TCNF-0.05GCA	13 days	1.11e5	15.9	11.2	16.2	0.862	1
2nd_set	25%_1.0NRL-0.12TCNF-0.03GCA	1 hour	8.20e4	15.5	10.7	16.7	0.886	1
2nd_set	25%_1.0NRL-0.12TCNF-0.03GCA	24 hours (1 day)	8.87e4	12.4	8.46	12.2	0.834	1
2nd_set	25%_1.0NRL-0.12TCNF-0.03GCA	3 days	1.07e5	16.5	13.8	17.2	0.94	1
2nd_set	25%_1.0NRL-0.12TCNF-0.03GCA	7 days	1.20e5	12	9.19	11.7	0.868	1
2nd_set	25%_1.0NRL-0.12TCNF-0.03GCA	14 days	1.39e5	18.4	14.1	17.5	0.86	1
2nd_set	25%_1.0NRL-0.15TCNF-0.0GCA	1 hour	1.33e5	16.3	12.7	16.8	0.906	1
2nd_set	25%_1.0NRL-0.15TCNF-0.0GCA	24 hours (1 day)	1.43e5	18.2	12.9	18.8	0.869	1
2nd_set	25%_1.0NRL-0.15TCNF-0.0GCA	4 days	1.68e5	16.9	14.3	16.2	0.902	1
2nd_set	25%_1.0NRL-0.15TCN	7 days	1.77e5	15	11.5	15.9	0.909	1

Set	Formulation	Age	Low-shear parameter (Pa·s)	Symmetric ±(%)	Bootstrap lower arm (%)	Bootstrap upper arm (%)	Bootstrap width/symmetric width	Exponent at bound
	F-0.0GCA							
2nd_set	25%_1.0NRL-0.15TCNF-0.0GCA	14 days	1.98e5	19.3	15.5	18.3	0.877	1
3rd_set	25%_1.0NRL-0.07TCNF-0.08GCA	1 hour	3.39e4	12.1	9.63	12.7	0.92	1
3rd_set	25%_1.0NRL-0.07TCNF-0.08GCA	24 hours (1 day)	3.70e4	10.4	7.26	9.32	0.797	1
3rd_set	25%_1.0NRL-0.07TCNF-0.08GCA	3 days	4.06e4	16.5	11.4	15	0.799	1
3rd_set	25%_1.0NRL-0.07TCNF-0.08GCA	7 days	5.07e4	18.7	13.5	16.8	0.812	1
3rd_set	25%_1.0NRL-0.07TCNF-0.08GCA	14 days	5.41e4	20.1	16.2	22.4	0.96	1
3rd_set	25%_1.0NRL-0.07TCNF-0.0GCA	1 hour	3.48e4	11.3	10.4	9.2	0.869	0
3rd_set	25%_1.0NRL-0.07TCNF-0.0GCA	24 hours (1 day)	4.25e4	10.5	7.55	9.06	0.79	1
3rd_set	25%_1.0NRL-0.07TCNF-0.0GCA	3 days	4.65e4	14.2	12.5	12.6	0.888	1

Set	Formulation	Age	Low-shear parameter (Pa·s)	Symmetric ±(%)	Bootstrap lower arm (%)	Bootstrap upper arm (%)	Bootstrap width/symmetric width	Exponent at bound
3rd_set	25%_1.0NRL-0.07TCNF-0.0GCA	7 days	5.79e4	16.2	12	15.8	0.857	1
3rd_set	25%_1.0NRL-0.07TCNF-0.0GCA	29 days	6.76e4	20	14.6	19.1	0.843	1
3rd_set	25%_1.0NRL-0.07TCNF-0.1GCA	1 hour	3.11e4	12.5	10.9	11	0.871	1
3rd_set	25%_1.0NRL-0.07TCNF-0.1GCA	24 hours	3.87e4	11.9	10.3	11.9	0.93	1
3rd_set	25%_1.0NRL-0.07TCNF-0.1GCA	3 days	4.12e4	14	10.4	15.2	0.916	1
3rd_set	25%_1.0NRL-0.07TCNF-0.1GCA	7 days	4.42e4	14.8	12.8	14.1	0.909	1
3rd_set	25%_1.0NRL-0.07TCNF-0.1GCA	29 days	4.86e4	15.2	11.7	14.7	0.867	1
3rd_set	25%_1.0NRL-0.07TCNF-0.15GCA	1 hour	3.29e4	15.5	12.1	14.1	0.845	1
3rd_set	25%_1.0NRL-0.07TCNF-0.15GCA	24 hours (1 day)	3.62e4	14.8	12.4	15.5	0.939	1
3rd_set	25%_1.0NRL-0.07TCN	3 days	4.01e4	19.2	16.1	18.9	0.913	1

Set	Formulation	Age	Low-shear parameter (Pa·s)	Symmetric ±(%)	Bootstrap lower arm (%)	Bootstrap upper arm (%)	Bootstrap width/symmetric width	Exponent at bound
	F-0.15GCA							
3rd_set	25%_1.0 NRL-0.07TCNF-0.15GCA	6 days	4.37e4	20.9	18.5	22.6	0.982	1
3rd_set	25%_1.0 NRL-0.07TCNF-0.15GCA	35 days	5.79e4	24.5	18.9	25.5	0.908	1
3rd_set	25%_1.0 NRL-0.07TCNF-0.2GCA	1 hour	3.28e4	14.9	11.9	14.6	0.888	1
3rd_set	25%_1.0 NRL-0.07TCNF-0.2GCA	24 hours (1 day)	3.49e4	16.6	12.7	16.8	0.892	1
3rd_set	25%_1.0 NRL-0.07TCNF-0.2GCA	3 days	3.81e4	22.4	18.8	22.1	0.912	1
3rd_set	25%_1.0 NRL-0.07TCNF-0.2GCA	6 days	4.47e4	23.2	19.3	21.6	0.882	1
3rd_set	25%_1.0 NRL-0.07TCNF-0.2GCA	35 days	5.88e4	30.8	21.4	36	0.933	1
3rd_set	25%_1.0 NRL-0.07TCNF-0.25GCA	1 hour	2.66e4	13.5	11.1	13.7	0.922	1
3rd_set	25%_1.0 NRL-0.07TCNF-0.25GCA	24 hours (1 day)	2.84e4	16.7	13.7	15.7	0.881	1

Set	Formulation	Age	Low-shear parameter (Pa·s)	Symmetric ±(%)	Bootstrap lower arm (%)	Bootstrap upper arm (%)	Bootstrap width/symmetric width	Exponent at bound
3rd_set	25%_1.0NRL-0.07TCNF-0.25GCA	3 days	3.48e4	23.1	18.3	22.9	0.893	1
3rd_set	25%_1.0NRL-0.07TCNF-0.25GCA	6 days	3.87e4	26.2	18.5	31.3	0.952	1
3rd_set	25%_1.0NRL-0.07TCNF-0.25GCA	35 days	5.14e4	33.4	24.3	34.9	0.886	1
4th_set	25%_1.0NRL-0.07TCNF-0.15GCA	1 hour	3.29e4	15.5	11.9	13.5	0.824	1
4th_set	25%_1.0NRL-0.07TCNF-0.15GCA	24 hours (1 day)	3.62e4	14.8	11.2	14.1	0.851	1
4th_set	25%_1.0NRL-0.07TCNF-0.15GCA	3 days	4.01e4	19.2	14.2	17.7	0.831	1
4th_set	25%_1.0NRL-0.07TCNF-0.15GCA	6 days	4.37e4	20.9	16.3	24	0.966	1
4th_set	25%_1.0NRL-0.07TCNF-0.15GCA	35 days	5.79e4	24.5	19	24.3	0.885	1
4th_set	25%_1.0NRL-0.07TCNF-0.15GNP	1 hour	1.02e5	39	23.8	59.7	1.07	1
4th_set	25%_1.0NRL-0.07TCN	24 hours (1 day)	1.32e5	38.4	24.8	45.5	0.914	1

Set	Formulation	Age	Low-shear parameter (Pa·s)	Symmetric ±(%)	Bootstrap lower arm (%)	Bootstrap upper arm (%)	Bootstrap width/symmetric width	Exponent at bound
	F-0.15GNP							
4th_set	25%_1.0 NRL-0.07TCNF-0.15GNP	3 days	1.50e5	28.7	19.5	35.9	0.966	1
4th_set	25%_1.0 NRL-0.07TCNF-0.15GNP	6 days	1.53e5	27	21.1	27.8	0.906	1
4th_set	25%_1.0 NRL-0.07TCNF-0.15GNP	10 days	1.63e5	25.7	20	28	0.937	1
4th_set	25%_1.0 NRL-0.07TCNF-0.15GNP	15 days	1.70e5	29.1	20.2	36.2	0.97	1
4th_set	25%_1.0 NRL-0.07TCNF-0.15Grpht	1 hour	2.87e4	14.1	12.2	12.7	0.884	1
4th_set	25%_1.0 NRL-0.07TCNF-0.15Grpht	24 hours (1 day)	3.30e4	14.2	11.2	14.8	0.918	1
4th_set	25%_1.0 NRL-0.07TCNF-0.15Grpht	4 days	4.01e4	20.4	17.7	19	0.901	1
4th_set	25%_1.0 NRL-0.07TCNF-0.15Grpht	8 days	4.74e4	18.7	12.8	18.6	0.84	1
4th_set	25%_1.0 NRL-0.07TCNF-0.15Grpht	14 days	4.67e4	23.4	17.6	23.9	0.886	1

Set	Formulation	Age	Low-shear parameter (Pa·s)	Symmetric ±(%)	Bootstrap lower arm (%)	Bootstrap upper arm (%)	Bootstrap width/symmetric width	Exponent at bound
4th_set	25%_1.0NRL-0.07TCNF-0.15MWNT-CO	1 hour	1.29e5	25.4	17.4	29.6	0.924	1
4th_set	25%_1.0NRL-0.07TCNF-0.15MWNT-CO	24 hours (1 day)	1.21e5	17.6	12.6	18.1	0.876	1
4th_set	25%_1.0NRL-0.07TCNF-0.15MWNT-CO	4 days	1.44e5	17	13.5	18.4	0.934	1
4th_set	25%_1.0NRL-0.07TCNF-0.15MWNT-CO	8 days	1.58e5	16.9	12.8	18.7	0.931	1
4th_set	25%_1.0NRL-0.07TCNF-0.15MWNT-CO	13 days	1.62e5	18.4	13.1	19.2	0.879	1
4th_set	25%_1.0NRL-0.07TCNF-0.15MWNT	1 hour	1.05e5	14.5	9.62	15.6	0.873	1
4th_set	25%_1.0NRL-0.07TCNF-0.15MWNT	24 hours (1 day)	1.06e5	12.2	9.11	13.5	0.927	1
4th_set	25%_1.0NRL-0.07TCNF-0.15MWNT	3 days	1.37e5	13.1	10.5	13.4	0.912	1

Set	Formulation	Age	Low-shear parameter (Pa·s)	Symmetric ±(%)	Bootstrap lower arm (%)	Bootstrap upper arm (%)	Bootstrap width/symmetric width	Exponent at bound
4th_set	25%_1.0NRL-0.07TCNF-0.15MWNT	6 days	1.49e5	13.1	9.13	14	0.882	1
4th_set	25%_1.0NRL-0.07TCNF-0.15MWNT	10 days	1.60e5	16.7	12.6	13.5	0.781	1
4th_set	25%_1.0NRL-0.07TCNF-0.15MWNT	15 days	3.38e5	38.3	24.7	46.5	0.93	1

Table S13. The additive comparison at matched storage times.

Each additive was measured at five or six storage times. The two properties were interpolated to 6 d and to 13 d, both of which lie inside the measured window of every formulation, so no value is extrapolated. The bracketing columns give the two measured ages between which each value falls, and a bracket such as 6-6 means the age was measured directly. The linear columns repeat the interpolation on a linear scale instead of a logarithmic one, as a check that the comparison does not depend on that choice.

Additive	G′ late (Pa)	Late age (d)	G′ at 6 d (Pa)	G′ at 6 d, linear	Brackets	G′ at 13 d (Pa)	G′ at 13 d, linear	Brackets	Low-shear late (Pa·s)	Low-shear at 6 d	Brackets	Low-shear at 13 d	Brackets
GCA	552	35	420	420	6-6	473	452	6-35	5.79e4	4.37e4	6-6	4.94e4	6-35
GNP	1.49e+03	15	1.16e+03	1.16e+03	6-6	1.41e+03	1.41e+03	10-15	1.70e5	1.53e5	6-6	1.68e5	10-15
MWCNT	2.51e+03	15	1.33e+03	1.33e+03	6-6	2.16e+03	2.16e+03	10-15	3.38e5	1.49e5	6-6	2.60e5	10-15

Additive	G′ late (Pa)	Late age (d)	G′ at 6 d (Pa)	G′ at 6 d, linear	Brackets	G′ at 13 d (Pa)	G′ at 13 d, linear	Brackets	Low-shear late (Pa·s)	Low-shear at 6 d	Brackets	Low-shear at 13 d	Brackets
MWCNT-COOH	1.49e+03	13	1.35e+03	1.34e+03	4-8	1.49e+03	1.49e+03	13-13	1.62e5	1.52e5	4-8	1.62e5	13-13
graphite	446	13	417	414	4-8	446	446	13-13	4.67e4	4.42e4	4-8	4.68e4	8-14

Figure S3. Set 4 against storage time.

a) Storage modulus at 1 Hz and b) the low-shear Cross parameter, for the five carbon additives, against storage time on logarithmic axes. The dotted vertical lines mark the two ages at which the additives are compared after matching, 6 d and 13 d, both of which lie inside the measured window of every formulation. The figure shows why the comparison has to be matched: the late-storage measurement of the MWCNT system falls at 15 d and that of the GCA system at 35 d, so an unmatched comparison reads a difference in storage time as a difference between additives.

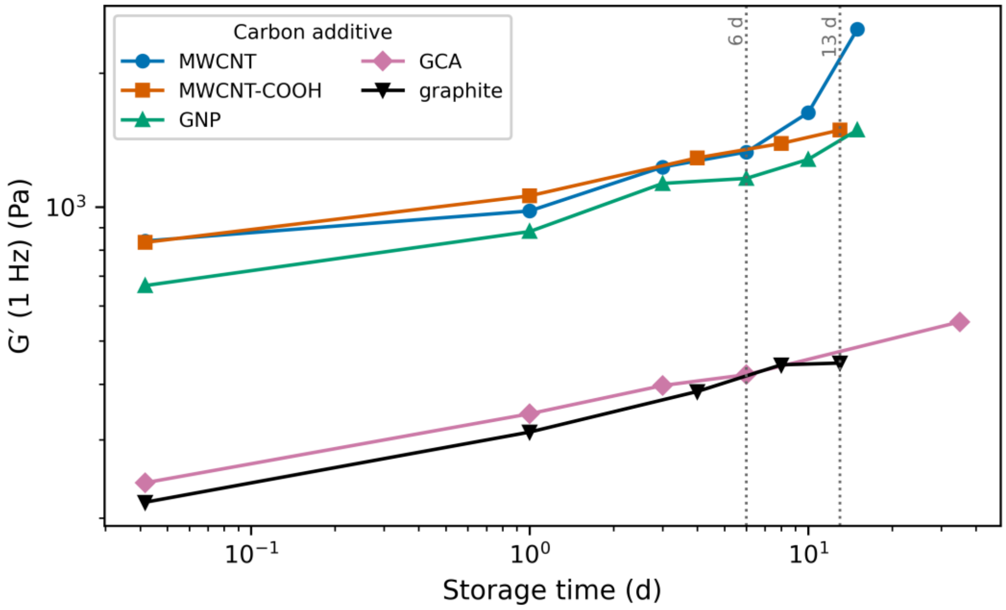

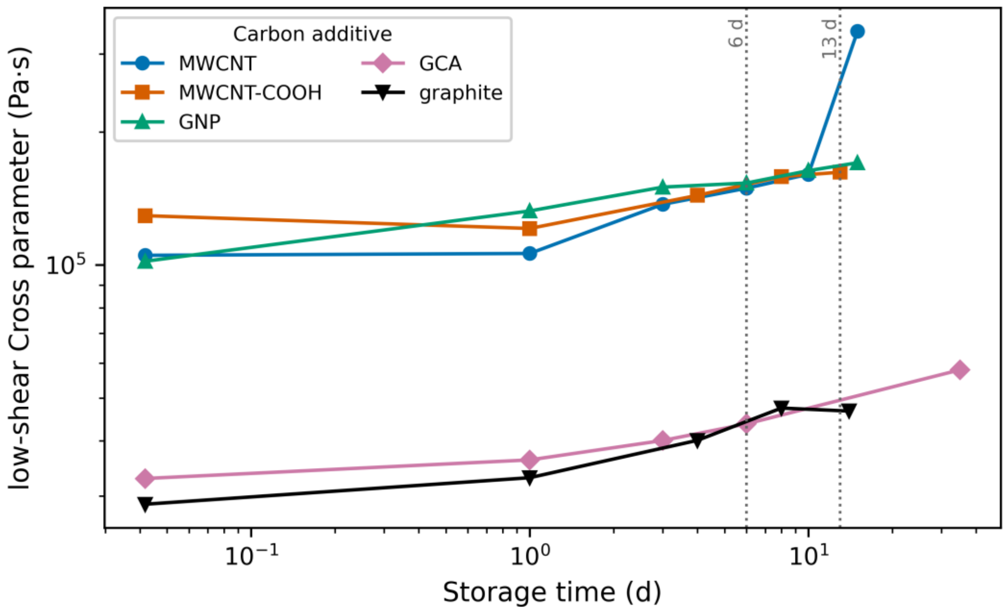

Table S14. All measured and fitted quantities, per formulation and storage time.

The oscillatory quantities are read at the measured point nearest 1 Hz, and the flow quantities are the parameters of the bounded Cross fit with their linearized 95 % intervals, which describe uncertainty in the fit only (Table S12 and Section 2.5). Where the exponent sits at its upper bound no interval is given for it. One of the 109 flow states, graphite at 14 d, has no oscillatory measurement at the same storage time, since the oscillatory series for that formulation ends at 13 d, and its oscillatory columns are therefore empty.

Set	Formulation	Age	Age (d)	G′(1 Hz) (Pa)	G″(1 Hz) (Pa)	tan δ (1 Hz)	η0 (Pa·s)	η0 ±CI 95	λ (s)	λ ±CI 95	n	n ±CI 95	n at bound	R2(log10 η)
1st_set	20%_1.0NRL-0.05TCNF-0.1GCA	1 hour	0.0417	34	3.88	0.114	4.77e+03	571	211	51	0.963	0.025	0	0.999

Set	Formulation	Age	Age (d)	G′(1 Hz) (Pa)	G″(1 Hz) (Pa)	tan δ (1 Hz)	η0 (Pa·s)	η0 ±CI95	λ (s)	λ ±CI95	n	n ±CI95	n at bound	R2(log10 η)
1st_set	20%_1.0NRL-0.05TCNF-0.1GCA	11 days	11	104	8.96	0.0865	8.9e+03	1.19e+03	198	52.2	1	nan	1	0.998
1st_set	20%_1.0NRL-0.05TCNF-0.1GCA	24 hours (1 day)	1	63.6	5.93	0.0932	6.08e+03	785	215	55.4	0.975	0.027	0	0.998
1st_set	20%_1.0NRL-0.05TCNF-0.1GCA	4 days	4	75.1	6.81	0.0907	6.76e+03	915	195	52.6	0.99	0.0297	0	0.998
1st_set	25%_1.0NRL-0.05TCNF-0.1GCA	1 hour	0.0417	99.2	9.4	0.0948	1.38e4	1.6e+03	304	66.7	0.988	0.0219	0	0.999
1st_set	25%_1.0NRL-0.05TCNF-0.1GCA	11 days	11	261	21.4	0.0819	2.55e4	4.34e+03	336	102	1	nan	1	0.998
1st_set	25%_1.0NRL-0.05TCN	24 hours (1 day)	1	175	14.9	0.085	1.81e4	2.13e+03	303	66.6	0.997	0.0223	0	0.999

Set	Formulation	Age	Age (d)	G′(1 Hz) (Pa)	G″(1 Hz) (Pa)	tan δ (1 Hz)	η0 (Pa·s)	η0 ±CI95	λ (s)	λ ±CI95	n	n ±CI95	n at bound	R2(log10 η)
	F-0.1GCA													
1st_set	25%_1.0NRL-0.05TCNF-0.1GCA	7 days	7	232	19.3	0.0832	2.24e4	3.3e+03	310	84.9	1	nan	1	0.998
1st_set	30%_1.0NRL-0.05TCNF-0.1GCA	1 hour	0.0417	235	21.6	0.0918	3.33e4	4.54e+03	503	119	0.992	0.0207	0	0.999
1st_set	30%_1.0NRL-0.05TCNF-0.1GCA	14 days	14	539	45.7	0.0849	5.84e4	8.91e+03	507	131	1	nan	1	0.999
1st_set	30%_1.0NRL-0.05TCNF-0.1GCA	24 hours (1 day)	1	364	32.9	0.0904	3.75e4	3.09e+03	443	63	1	nan	1	1
1st_set	30%_1.0NRL-0.05TCNF-0.1GCA	4 days	4	442	38.9	0.0879	4.73e4	6.52e+03	496	117	1	nan	1	0.999
1st_set	30%_1.0NRL	7 days	7	465	40.4	0.0869	4.40e4	5.12e+03	428	86.2	1	nan	1	0.999

Set	Formulation	Age	Age (d)	G′(1 Hz) (Pa)	G″(1 Hz) (Pa)	tan δ (1 Hz)	η0 (Pa·s)	η0 ±CI95	λ (s)	λ ±CI95	n	n ±CI95	n at bound	R2(log10 η)
	-0.05TCNF-0.1GCA													
1st_set	35%_1.0NRL-0.05TCNF-0.1GCA	1 hour	0.0417	544	51.6	0.0948	7.34e4	8.3e+03	659	123	1	nan	1	0.999
1st_set	35%_1.0NRL-0.05TCNF-0.1GCA	11 days	11	922	86.1	0.0935	9.87e4	1.25e4	667	139	1	nan	1	0.999
1st_set	35%_1.0NRL-0.05TCNF-0.1GCA	24 hours (1 day)	1	703	68.3	0.0972	7.84e4	6.7e+03	613	87.2	1	nan	1	1
1st_set	35%_1.0NRL-0.05TCNF-0.1GCA	7 days	7	909	85.4	0.0939	9.69e4	1.01e4	638	110	1	nan	1	1
1st_set	40%_1.0NRL-0.05TCNF-0.1GCA	1 hour	0.0417	657	74.9	0.114	7.32e4	5.19e+03	764	89	1	nan	1	1

Set	Formulation	Age	Age (d)	G′(1 Hz) (Pa)	G″(1 Hz) (Pa)	tan δ (1 Hz)	η0 (Pa·s)	η0 ±CI95	λ (s)	λ ±CI95	n	n ±CI95	n at bound	R2(log10 η)
1st_set	40%_1.0NRL-0.05TCNF-0.1GCA	14 days	14	1.05e+03	117	0.112	1.01e5	8.01e+03	751	97.3	1	nan	1	1
1st_set	40%_1.0NRL-0.05TCNF-0.1GCA	24 hours (1 day)	1	793	92	0.116	7.67e4	5.05e+03	728	79.1	1	nan	1	1
1st_set	40%_1.0NRL-0.05TCNF-0.1GCA	4 days	4	799	91.9	0.115	8.17e4	8.5e+03	768	130	1	nan	1	1
1st_set	40%_1.0NRL-0.05TCNF-0.1GCA	7 days	7	913	104	0.114	8.99e4	6.26e+03	744	84.8	1	nan	1	1
1st_set	40%_1.0NRL-0.05TCNF-0.1GCA	9 days	9	990	111	0.112	1.01e5	7.66e+03	802	99	1	nan	1	1
2nd_set	25%_1.0NRL-0.03TCN	1 hour	0.0417	35.1	4.43	0.126	4.08e+03	350	251	42.7	0.972	0.0177	0	0.999

Set	Formulation	Age	Age (d)	G′(1 Hz) (Pa)	G″(1 Hz) (Pa)	tan δ (1 Hz)	η_0 (Pa·s)	η_0 ±CI95	λ (s)	λ ±CI95	n	n ±CI95	n at bound	R^2($\log_{10}$ η)
	F-0.12GCA													
2nd_set	25%_1.0NRL-0.03TCNF-0.12GCA	13 days	13	103	10.6	0.103	9.64e+03	1.1e+03	291	62.9	1	nan	1	0.999
2nd_set	25%_1.0NRL-0.03TCNF-0.12GCA	24 hours (1 day)	1	57.5	6.27	0.109	5.34e+03	612	256	57.4	0.983	0.0237	0	0.999
2nd_set	25%_1.0NRL-0.03TCNF-0.12GCA	3 days	3	72.5	7.81	0.108	6.86e+03	831	266	62.3	0.989	0.0246	0	0.999
2nd_set	25%_1.0NRL-0.03TCNF-0.12GCA	6 days	6	92.1	9.12	0.0991	9.16e+03	1.36e+03	290	81.4	1	nan	1	0.998
2nd_set	25%_1.0NRL-0.05TCNF-	1 hour	0.0417	99.2	9.4	0.0948	1.38e4	1.6e+03	304	66.7	0.988	0.0219	0	0.999

Set	Formulation	Age	Age (d)	G′(1 Hz) (Pa)	G″(1 Hz) (Pa)	tan δ (1 Hz)	η0 (Pa·s)	η0 ±CI95	λ (s)	λ ±CI95	n	n ±CI95	n at bound	R2(log10 η)
	0.1GCA													
2nd_set	25%_1.0NRL-0.05TCNF-0.1GCA	11 days	11	261	21.4	0.0819	2.55e4	4.34e+03	336	102	1	nan	1	0.998
2nd_set	25%_1.0NRL-0.05TCNF-0.1GCA	24 hours (1 day)	1	175	14.9	0.085	1.81e4	2.13e+03	303	66.6	0.997	0.0223	0	0.999
2nd_set	25%_1.0NRL-0.05TCNF-0.1GCA	7 days	7	232	19.3	0.0832	2.24e4	3.3e+03	310	84.9	1	nan	1	0.998
2nd_set	25%_1.0NRL-0.07TCNF-0.08GCA	1 hour	0.0417	269	23.9	0.0888	3.39e4	4.11e+03	360	77.3	1	nan	1	0.999
2nd_set	25%_1.0NRL-0.07TCNF-0.08GCA	14 days	14	513	42.7	0.0832	5.41e4	1.09e4	445	155	1	nan	1	0.998
2nd_set	25%_1.0	24 hour	1	362	30.9	0.0855	3.70e4	3.85e+03	345	63.7	1	nan	1	0.999

Set	Formulation	Age	Age (d)	G′(1 Hz) (Pa)	G″(1 Hz) (Pa)	tan δ (1 Hz)	η0 (Pa·s)	η0 ±CI95	λ (s)	λ ±CI95	n	n ±CI95	n at bound	R2(log10 η)
	NRL-0.07TCNF-0.08GCA	s (1 day)												
2nd_set	25%_1.0NRL-0.07TCNF-0.08GCA	3 days	3	421	35.6	0.0845	4.06e4	6.69e+03	351	103	1	nan	1	0.998
2nd_set	25%_1.0NRL-0.07TCNF-0.08GCA	7 days	7	481	40	0.0832	5.07e4	9.47e+03	409	133	1	nan	1	0.998
2nd_set	25%_1.0NRL-0.10TCNF-0.05GCA	1 hour	0.0417	540	47	0.0871	7.50e4	1.12e4	504	128	1	nan	1	0.999
2nd_set	25%_1.0NRL-0.10TCNF-0.05GCA	13 days	13	1.04e+03	90.5	0.0871	1.11e5	1.77e4	558	149	1	nan	1	0.999
2nd_set	25%_1.0NRL	24 hour	1	716	61.7	0.0862	7.72e4	9.37e+03	462	96.1	1	nan	1	0.999

Set	Formulation	Age	Age (d)	G′(1 Hz) (Pa)	G″(1 Hz) (Pa)	tan δ (1 Hz)	η0 (Pa·s)	η0 ±CI95	λ (s)	λ ±CI95	n	n ±CI95	n at bound	R2(log10 η)
	-0.10TCNF-0.05GCA	s (1 day)												
2nd_set	25%_1.0NRL-0.10TCNF-0.05GCA	3 days	3	761	66.5	0.0874	7.61e4	8.7e+03	434	85.7	1	nan	1	0.999
2nd_set	25%_1.0NRL-0.10TCNF-0.05GCA	6 days	6	798	71.6	0.0896	8.10e4	9.45e+03	456	91.4	1	nan	1	0.999
2nd_set	25%_1.0NRL-0.12TCNF-0.03GCA	1 hour	0.0417	658	68	0.103	8.20e4	1.27e4	596	154	1	nan	1	0.999
2nd_set	25%_1.0NRL-0.12TCNF-0.03GCA	14 days	14	1.33e+03	141	0.106	1.39e5	2.55e4	641	195	1	nan	1	0.999
2nd_set	25%_1.0NRL-	24 hours (1 day)	1	846	91.5	0.108	8.87e4	1.10e4	547	114	1	nan	1	0.999

Set	Formulation	Age	Age (d)	G′(1 Hz) (Pa)	G″(1 Hz) (Pa)	tan δ (1 Hz)	η0 (Pa·s)	η0 ±CI95	λ (s)	λ ±CI95	n	n ±CI95	n at bound	R2(log10 η)
	0.12TCNF-0.03GCA													
2nd_set	25%_1.0NRL-0.12TCNF-0.03GCA	3 days	3	990	104	0.105	1.07e5	1.77e4	623	170	1	nan	1	0.999
2nd_set	25%_1.0NRL-0.12TCNF-0.03GCA	7 days	7	1.09e+03	117	0.108	1.20e5	1.44e4	578	117	1	nan	1	0.999
2nd_set	25%_1.0NRL-0.15TCNF-0.0GCA	1 hour	0.0417	1.14e+03	138	0.121	1.33e5	2.17e4	723	192	1	nan	1	0.999
2nd_set	25%_1.0NRL-0.15TCNF-0.0GCA	14 days	14	1.88e+03	246	0.131	1.98e5	3.82e4	831	257	1	nan	1	0.999
2nd_set	25%_1.0NRL-0.15TCNF-	24 hours (1 day)	1	1.45e+03	181	0.124	1.43e5	2.61e4	714	213	1	nan	1	0.999

Set	Formulation	Age	Age (d)	G′(1 Hz) (Pa)	G″(1 Hz) (Pa)	tan δ (1 Hz)	η0 (Pa·s)	η0 ±CI95	λ (s)	λ ±CI95	n	n ±CI95	n at bound	R2(log10 η)
	0.0GCA													
2nd_set	25%_1.0NRL-0.15TCNF-0.0GCA	4 days	4	1.61e+03	200	0.124	1.68e5	2.85e4	734	202	1	nan	1	0.999
2nd_set	25%_1.0NRL-0.15TCNF-0.0GCA	7 days	7	1.72e+03	229	0.133	1.77e5	2.65e4	722	177	1	nan	1	0.999
3rd_set	25%_1.0NRL-0.07TCNF-0.08GCA	1 hour	0.0417	269	23.9	0.0888	3.39e4	4.11e+03	360	77.3	1	nan	1	0.999
3rd_set	25%_1.0NRL-0.07TCNF-0.08GCA	14 days	14	513	42.7	0.0832	5.41e4	1.09e4	445	155	1	nan	1	0.998
3rd_set	25%_1.0NRL-0.07TCNF-0.08GCA	24 hours (1 day)	1	362	30.9	0.0855	3.70e4	3.85e+03	345	63.7	1	nan	1	0.999

Set	Formulation	Age	Age (d)	G′(1 Hz) (Pa)	G″(1 Hz) (Pa)	tan δ (1 Hz)	η0 (Pa·s)	η0 ±CI95	λ (s)	λ ±CI95	n	n ±CI95	n at bound	R2(log10 η)
3rd_set	25%_1.0NRL-0.07TCNF-0.08GCA	3 days	3	421	35.6	0.0845	4.06e4	6.69e+03	351	103	1	nan	1	0.998
3rd_set	25%_1.0NRL-0.07TCNF-0.08GCA	7 days	7	481	40	0.0832	5.07e4	9.47e+03	409	133	1	nan	1	0.998
3rd_set	25%_1.0NRL-0.07TCNF-0.0GCA	1 hour	0.0417	259	22.4	0.0867	3.48e4	3.91e+03	397	78.8	0.993	0.0181	0	0.999
3rd_set	25%_1.0NRL-0.07TCNF-0.0GCA	24 hours (1 day)	1	414	33.4	0.0807	4.25e4	4.47e+03	395	72.6	1	nan	1	0.999
3rd_set	25%_1.0NRL-0.07TCNF-0.0GCA	29 days	29	598	47.1	0.0788	6.76e4	1.35e4	532	181	1	nan	1	0.998
3rd_set	25%_1.0NRL-	3 days	3	468	37.9	0.0811	4.65e4	6.59e+03	418	103	1	nan	1	0.999

Set	Formulation	Age	Age (d)	G′(1 Hz) (Pa)	G″(1 Hz) (Pa)	tan δ (1 Hz)	η0 (Pa·s)	η0 ±CI95	λ (s)	λ ±CI95	n	n ±CI95	n at bound	R2(log10 η)
	0.07TCNF-0.0GCA													
3rd_set	25%_1.0NRL-0.07TCNF-0.0GCA	7 days	7	528	42.8	0.0811	5.79e4	9.4e+03	508	141	1	nan	1	0.999
3rd_set	25%_1.0NRL-0.07TCNF-0.1GCA	1 hour	0.0417	269	23.5	0.0873	3.11e4	3.9e+03	366	80.7	1	nan	1	0.999
3rd_set	25%_1.0NRL-0.07TCNF-0.1GCA	24 hours	1	333	28.4	0.0852	3.87e4	4.61e+03	365	76.7	1	nan	1	0.999
3rd_set	25%_1.0NRL-0.07TCNF-0.1GCA	29 days	29	487	42.1	0.0865	4.86e4	7.38e+03	416	110	1	nan	1	0.999
3rd_set	25%_1.0NRL-0.07TCNF-0.1GCA	3 days	3	421	37.4	0.0889	4.12e4	5.76e+03	367	90.2	1	nan	1	0.999

Set	Formulation	Age	Age (d)	G′(1 Hz) (Pa)	G″(1 Hz) (Pa)	tan δ (1 Hz)	η0 (Pa·s)	η0 ±CI95	λ (s)	λ ±CI95	n	n ±CI95	n at bound	R2(log10 η)
3rd_set	25%_1.0NRL-0.07TCNF-0.1GCA	7 days	7	444	39.3	0.0883	4.42e4	6.54e+03	353	92.4	1	nan	1	0.999
3rd_set	25%_1.0NRL-0.07TCNF-0.15GCA	1 hour	0.0417	240	19.6	0.0815	3.29e4	5.08e+03	363	99	1	nan	1	0.999
3rd_set	25%_1.0NRL-0.07TCNF-0.15GCA	24 hours (1 day)	1	343	28.4	0.0828	3.62e4	5.37e+03	341	89.7	1	nan	1	0.999
3rd_set	25%_1.0NRL-0.07TCNF-0.15GCA	3 days	3	397	31.9	0.0804	4.01e4	7.69e+03	349	119	1	nan	1	0.998
3rd_set	25%_1.0NRL-0.07TCNF-0.15GCA	35 days	35	552	43.6	0.079	5.79e4	1.42e4	404	172	1	nan	1	0.997
3rd_set	25%_1.0	6 days	6	420	34.6	0.0823	4.37e4	9.13e+03	371	136	1	nan	1	0.997

Set	Formulation	Age	Age (d)	G′(1 Hz) (Pa)	G″(1 Hz) (Pa)	tan δ (1 Hz)	η0 (Pa·s)	η0 ±CI95	λ (s)	λ ±CI95	n	n ±CI95	n at bound	R2(log10 η)
	NRL-0.07TCNF-0.15GCA													
3rd_set	25%_1.0NRL-0.07TCNF-0.2GCA	1 hour	0.0417	222	19	0.0857	3.28e4	4.88e+03	373	97.7	1	nan	1	0.999
3rd_set	25%_1.0NRL-0.07TCNF-0.2GCA	24 hours (1 day)	1	324	26.3	0.0811	3.49e4	5.78e+03	335	98.5	1	nan	1	0.998
3rd_set	25%_1.0NRL-0.07TCNF-0.2GCA	3 days	3	383	30.7	0.0803	3.81e4	8.55e+03	335	134	1	nan	1	0.997
3rd_set	25%_1.0NRL-0.07TCNF-0.2GCA	35 days	35	538	42.4	0.0787	5.88e4	1.81e4	416	222	1	nan	1	0.995
3rd_set	25%_1.0NRL-0.07TCNF-	6 days	6	451	35.4	0.0785	4.47e4	1.04e4	353	145	1	nan	1	0.997

Set	Formulation	Age	Age (d)	G′(1 Hz) (Pa)	G″(1 Hz) (Pa)	tan δ (1 Hz)	η0 (Pa·s)	η0 ±CI95	λ (s)	λ ±CI95	n	n ±CI95	n at bound	R2(log10 η)
	0.2GCA													
3rd_set	25%_1.0NRL-0.07TCNF-0.25GCA	1 hour	0.0417	212	19.5	0.0918	2.66e4	3.59e+03	331	79.4	1	nan	1	0.999
3rd_set	25%_1.0NRL-0.07TCNF-0.25GCA	24 hours (1 day)	1	292	25.7	0.0881	2.84e4	4.74e+03	298	89.4	1	nan	1	0.998
3rd_set	25%_1.0NRL-0.07TCNF-0.25GCA	3 days	3	337	29	0.0863	3.48e4	8.05e+03	348	142	1	nan	1	0.997
3rd_set	25%_1.0NRL-0.07TCNF-0.25GCA	35 days	35	482	39.9	0.0827	5.14e4	1.72e4	386	227	1	nan	1	0.994
3rd_set	25%_1.0NRL-0.07TCNF-0.25	6 days	6	377	32.7	0.0866	3.87e4	1.01e4	355	164	1	nan	1	0.996

Set	Formulation	Age	Age (d)	G′(1 Hz) (Pa)	G″(1 Hz) (Pa)	tan δ (1 Hz)	η0 (Pa·s)	η0 ±CI95	λ (s)	λ ±CI95	n	n ±CI95	n at bound	R2(log10 η)
	GCA													
4th_set	25%_1.0NRL-0.07TCNF-0.15GCA	1 hour	0.0417	240	19.6	0.0815	3.29e4	5.08e+03	363	99	1	nan	1	0.999
4th_set	25%_1.0NRL-0.07TCNF-0.15GCA	24 hours (1 day)	1	343	28.4	0.0828	3.62e4	5.37e+03	341	89.7	1	nan	1	0.999
4th_set	25%_1.0NRL-0.07TCNF-0.15GCA	3 days	3	397	31.9	0.0804	4.01e4	7.69e+03	349	119	1	nan	1	0.998
4th_set	25%_1.0NRL-0.07TCNF-0.15GCA	35 days	35	552	43.6	0.079	5.79e4	1.42e4	404	172	1	nan	1	0.997
4th_set	25%_1.0NRL-0.07TCNF-0.15	6 days	6	420	34.6	0.0823	4.37e4	9.13e+03	371	136	1	nan	1	0.997

Set	Formulation	Age	Age (d)	G′(1 Hz) (Pa)	G″(1 Hz) (Pa)	tan δ (1 Hz)	η0 (Pa·s)	η0 ±CI95	λ (s)	λ ±CI95	n	n ±CI95	n at bound	R2(log10 η)
	GCA													
4th_set	25%_1.0NRL-0.07TCNF-0.15GNP	1 hour	0.0417	666	51.5	0.0772	1.02e5	3.97e4	607	394	1	nan	1	0.993
4th_set	25%_1.0NRL-0.07TCNF-0.15GNP	10 days	10	1.28e+03	94.3	0.0738	1.63e5	4.19e4	542	234	1	nan	1	0.997
4th_set	25%_1.0NRL-0.07TCNF-0.15GNP	15 days	15	1.49e+03	113	0.0753	1.70e5	4.96e4	391	203	1	nan	1	0.995
4th_set	25%_1.0NRL-0.07TCNF-0.15GNP	24 hours (1 day)	1	881	63.3	0.0719	1.32e5	5.09e4	663	420	1	nan	1	0.994
4th_set	25%_1.0NRL-0.07TCNF-0.15GNP	3 days	3	1.13e+03	89	0.0788	1.50e5	4.30e4	570	274	1	nan	1	0.996
4th_set	25%_1.0NRL-	6 days	6	1.16e+03	97.1	0.0834	1.53e5	4.14e4	546	248	1	nan	1	0.997

Set	Formulation	Age	Age (d)	G′(1 Hz) (Pa)	G″(1 Hz) (Pa)	tan δ (1 Hz)	η0 (Pa·s)	η0 ±CI95	λ (s)	λ ±CI95	n	n ±CI95	n at bound	R2(log10 η)
	0.07TCNF-0.15GNP													
4th_set	25%_1.0NRL-0.07TCNF-0.15graphite	1 hour	0.0417	217	21.1	0.0973	2.87e4	4.05e+03	329	82.7	1	nan	1	0.999
4th_set	25%_1.0NRL-0.07TCNF-0.15graphite	14 days	14	nan	nan	nan	4.67e4	1.09e4	414	169	1	nan	1	0.997
4th_set	25%_1.0NRL-0.07TCNF-0.15graphite	24 hours (1 day)	1	312	28.4	0.0911	3.30e4	4.69e+03	335	84.5	1	nan	1	0.999
4th_set	25%_1.0NRL-0.07TCNF-0.15graphite	4 days	4	385	34.3	0.0891	4.01e4	8.17e+03	376	135	1	nan	1	0.997
4th_set	25%_1.0NRL-0.07TCN	8 days	8	442	38.9	0.088	4.74e4	8.86e+03	396	129	1	nan	1	0.998

Set	Formulation	Age	Age (d)	G′(1 Hz) (Pa)	G″(1 Hz) (Pa)	tan δ (1 Hz)	η0 (Pa·s)	η0 ±CI95	λ (s)	λ ±CI95	n	n ±CI95	n at bound	R2(log10 η)
	F-0.15graphite													
4th_set	25%_1.0NRL-0.07TCNF-0.15MWCNT-COOH	1 hour	0.0417	833	79.4	0.0953	1.29e5	3.29e4	683	285	1	nan	1	0.997
4th_set	25%_1.0NRL-0.07TCNF-0.15MWCNT-COOH	13 days	13	1.49e+03	151	0.101	1.62e5	2.98e4	586	180	1	nan	1	0.998
4th_set	25%_1.0NRL-0.07TCNF-0.15MWCNT-COOH	24 hours (1 day)	1	1.06e+03	107	0.101	1.21e5	2.12e4	562	166	1	nan	1	0.999
4th_set	25%_1.0NRL-0.07TCNF-0.15	4 days	4	1.29e+03	138	0.107	1.44e5	2.45e4	570	163	1	nan	1	0.999

Set	Formulation	Age	Age (d)	G′(1 Hz) (Pa)	G″(1 Hz) (Pa)	tan δ (1 Hz)	η_0 (Pa·s)	η_0 ±CI95	λ (s)	λ ±CI95	n	n ±CI95	n at bound	R^2($\log_{10}$ η)
	MWCNT-COOH													
4th_set	25%_1.0NRL-0.07TCNF-0.15MWCNT-COOH	8 days	8	1.39e+03	153	0.11	1.58e5	2.67e4	561	159	1	nan	1	0.999
4th_set	25%_1.0NRL-0.07TCNF-0.15MWCNT	1 hour	0.0417	839	89.6	0.107	1.05e5	1.52e4	622	150	1	nan	1	0.999
4th_set	25%_1.0NRL-0.07TCNF-0.15MWCNT	10 days	10	1.63e+03	178	0.11	1.60e5	2.68e4	489	139	1	nan	1	0.999
4th_set	25%_1.0NRL-0.07TCNF-0.15MWCNT	15 days	15	2.51e+03	254	0.101	3.38e5	1.29e5	1.07e+03	637	1	nan	1	0.996
4th_set	25%_1.0NRL	24 hour	1	980	124	0.127	1.06e5	1.30e4	517	108	1	nan	1	0.999

Set	Formulation	Age	Age (d)	G′(1 Hz) (Pa)	G″(1 Hz) (Pa)	tan δ (1 Hz)	η0 (Pa·s)	η0 ±CI95	λ (s)	λ ±CI95	n	n ±CI95	n at bound	R2(log10 η)
	-0.07TCNF-0.15MWCNT	s (1 day)												
4th_set	25%_1.0NRL-0.07TCNF-0.15MWCNT	3 days	3	1.23e+03	150	0.122	1.37e5	1.79e4	582	127	1	nan	1	0.999
4th_set	25%_1.0NRL-0.07TCNF-0.15MWCNT	6 days	6	1.33e+03	150	0.113	1.49e5	1.95e4	570	126	1	nan	1	0.999

Figure S4. Residuals of the Cross fits.

All 109 fits, as a) the residual of log10 η and b) the same residual expressed as a percentage of the fitted viscosity, against shear rate. Grey lines are individual curves and the colored line is their median. The residuals are structured rather than random, which is what the high coefficient of determination alone does not reveal: the median reaches +0.10 in log10 η near 0.04 s-1 and -0.11 near 2.5 s-1, that is +26 % and -23 % in viscosity. Below 1e-3 s-1, the region from which the low-shear parameter is taken, the median residual stays within 5 %.

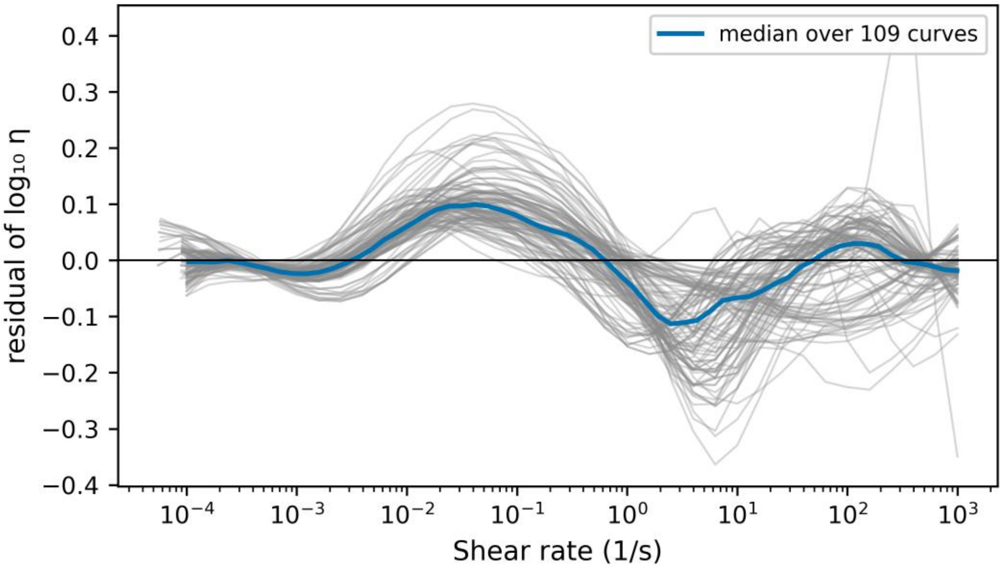

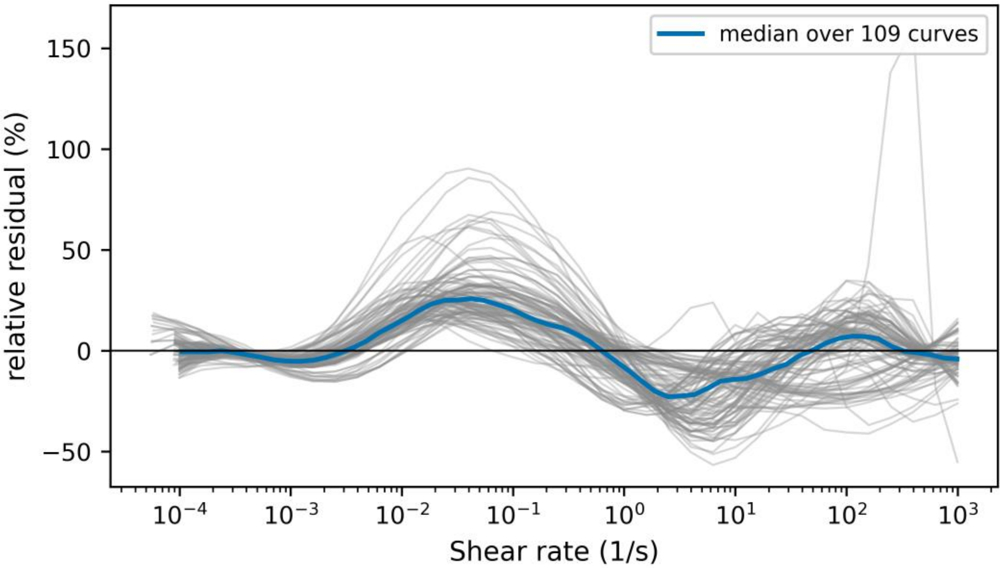

Figure S5. Residuals of the generalized Maxwell fits on two scales.

Residuals of the storage modulus for all 22 formulations at their late-storage state, as a) a percentage of the measured value and b) an absolute difference in pascals, against frequency. Grey lines are individual formulations and the colored line is their median. The shaded band marks the region below 0.1 Hz where the deviation is systematic: the fit underpredicts the storage modulus

by a median of 9 % at 0.01 Hz, the deviation changes sign near 0.09 Hz, and the pattern is nearly the same in every formulation. This is a further reason to treat the fitted spectrum as a smooth representation and not to read individual modes as physical.

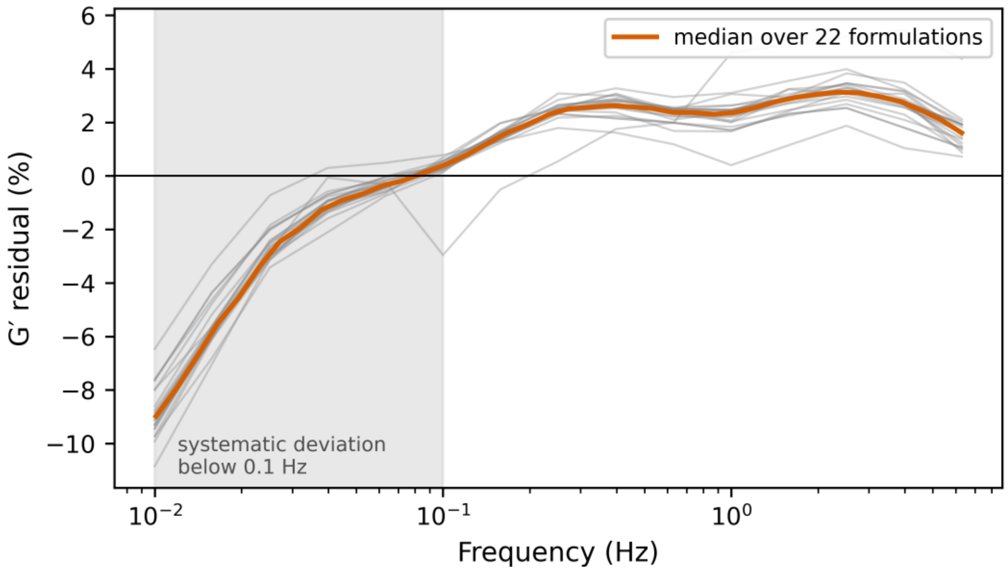

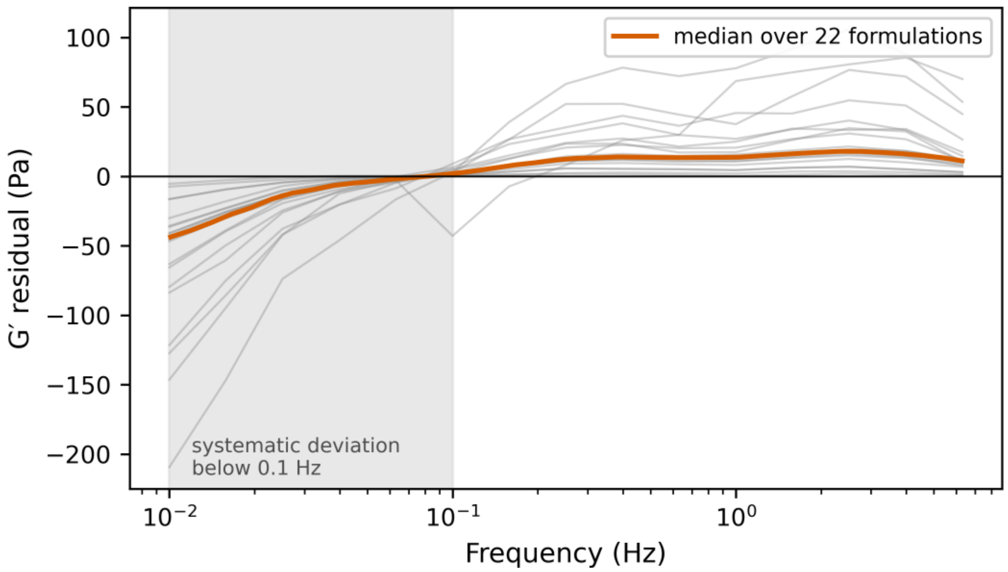

Figure S6. What the Cox–Merz deviation tracks.

Mean ratio of complex to steady-shear viscosity against the low-shear Cross parameter at the late-storage state, for the 19 distinct preparations, with the four sets marked. The deviation follows the strength of the network rather than the composition variable that produced it, at a Spearman coefficient of 0.74 against the low-shear parameter and 0.77 against G′(1 Hz). The overlapping range of the comparison is identical for every formulation (Table S8), so the accessible window cannot account for the trend.

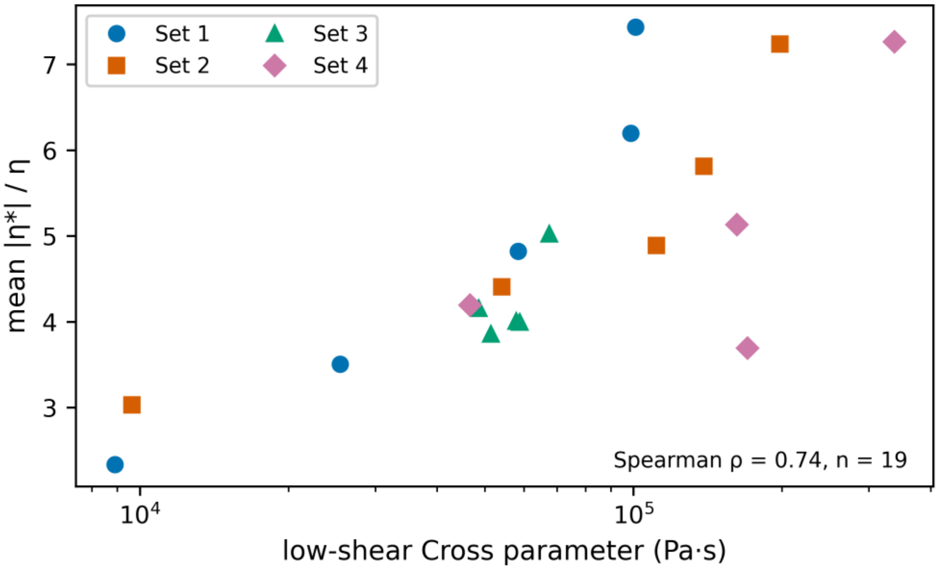

Supplement: Supplementary file 1 [file gels-12-00877-s001.zip › gels-4547171-supplementary.pdf]
